# Supplementary material for: Full-length transcript sequencing of human and mouse cerebral cortex identifies widespread isoform diversity and alternative splicing
Source: Cell Rep. 2021 Nov 16;37(7):110022. doi: 10.1016/j.celrep.2021.110022 (PMC8609283; doi:10.1016/j.celrep.2021.110022)
Supplement: Document S1. Figures S1–S16 and Tables S1, S3, S6, S8, S12, S17, S19, and S22 [file mmc1.pdf]

Cell Reports, Volume *37*

Supplemental information

Full-length transcript sequencing of human and mouse cerebral cortex identifies widespread isoform diversity and alternative splicing

Szi Kay Leung, Aaron R. Jeffries, Isabel Castanho, Ben T. Jordan, Karen Moore, Jonathan P. Davies, Emma L. Dempster, Nicholas J. Bray, Paul O'Neill, Elizabeth Tseng, Zeshan Ahmed, David A. Collier, Erin D. Jeffery, Shyam Prabhakar, Leonard Schalkwyk, Connor Jops, Michael J. Gandal, Gloria M. Sheynkman, Eilis Hannon, and Jonathan Mill

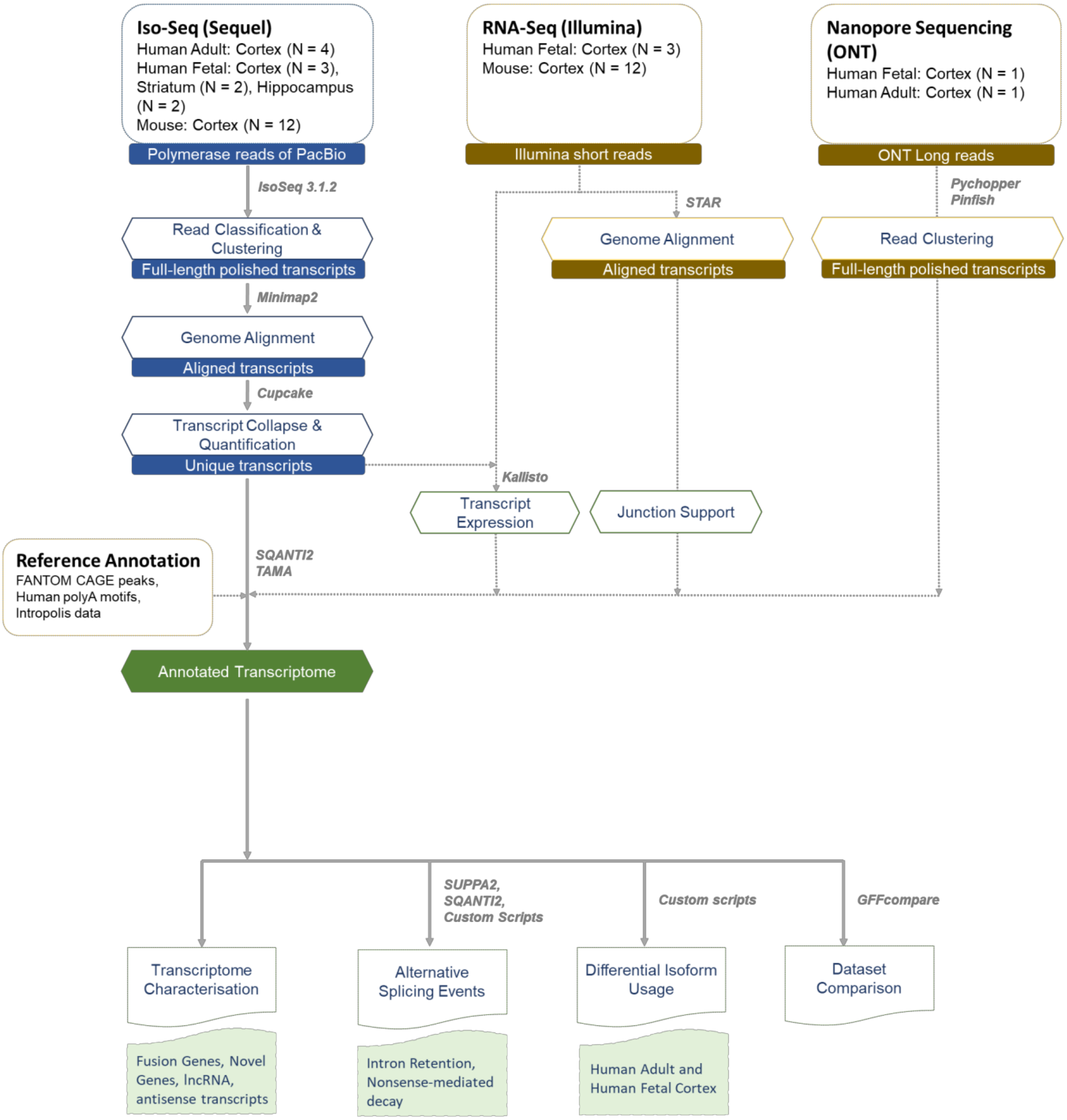

Figure S1: An overview of the analysis pipeline used to generate full-length transcript annotations in human and mouse cerebral cortex samples, related to STAR Methods. Briefly, polymerase reads from PacBio Sequel for each dataset were processed using *Iso-Seq 3.1.2* and *Cupcake* scripts to generate high quality, full-length isoforms. *SQANTI2* was used to fully annotate individual isoforms, with comparison to short-read RNA-Seq, ONT nanopore sequencing, and reference annotations. PacBio – Pacific Biosciences, ONT – Oxford Nanopore Technology

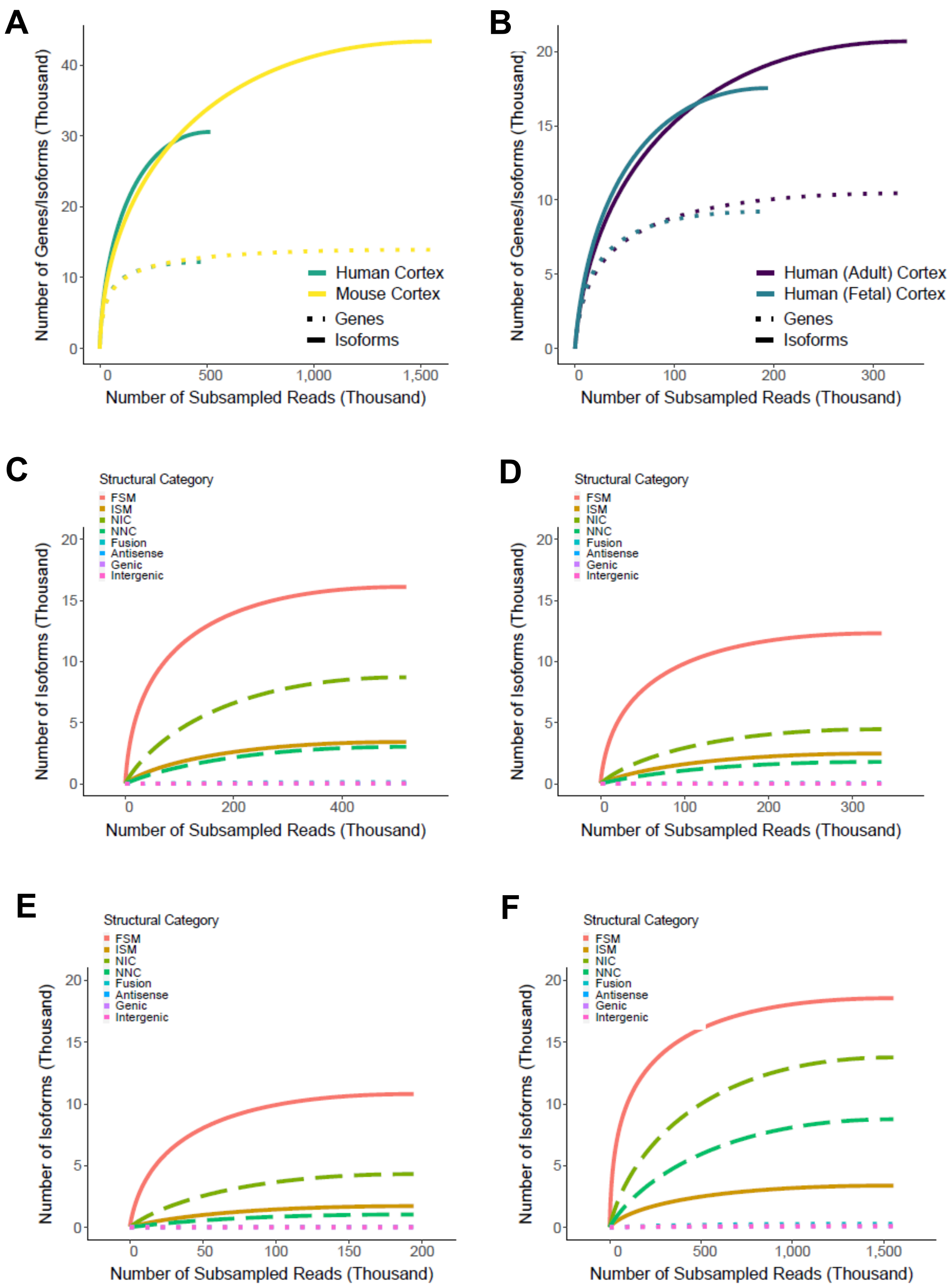

Figure S2: Saturation is reached across all Iso-Seq datasets at the gene and isoform level, related to STAR Methods and Table 1. We subsampled reads to generate rarefaction curves, using *cDNA Cupcake* scripts, at the gene and isoform level. Shown are comparisons between **A)** human (n = 7 biologically independent samples) and mouse cortex (n = 12 biologically independent samples) and **B)** human adult (n = 4 biologically independent samples) and human fetal cortex (n = 3 biologically independent samples). Also shown are rarefaction curves for each *SQANTI2* isoform category in **C)** human cortex, **D)** human adult cortex, **E)** human fetal cortex and **F)** mouse cortex. FSM – Full splice match, ISM – Incomplete Splice Match, NIC – Novel In Catalogue, NNC – Novel Not in Catalogue.

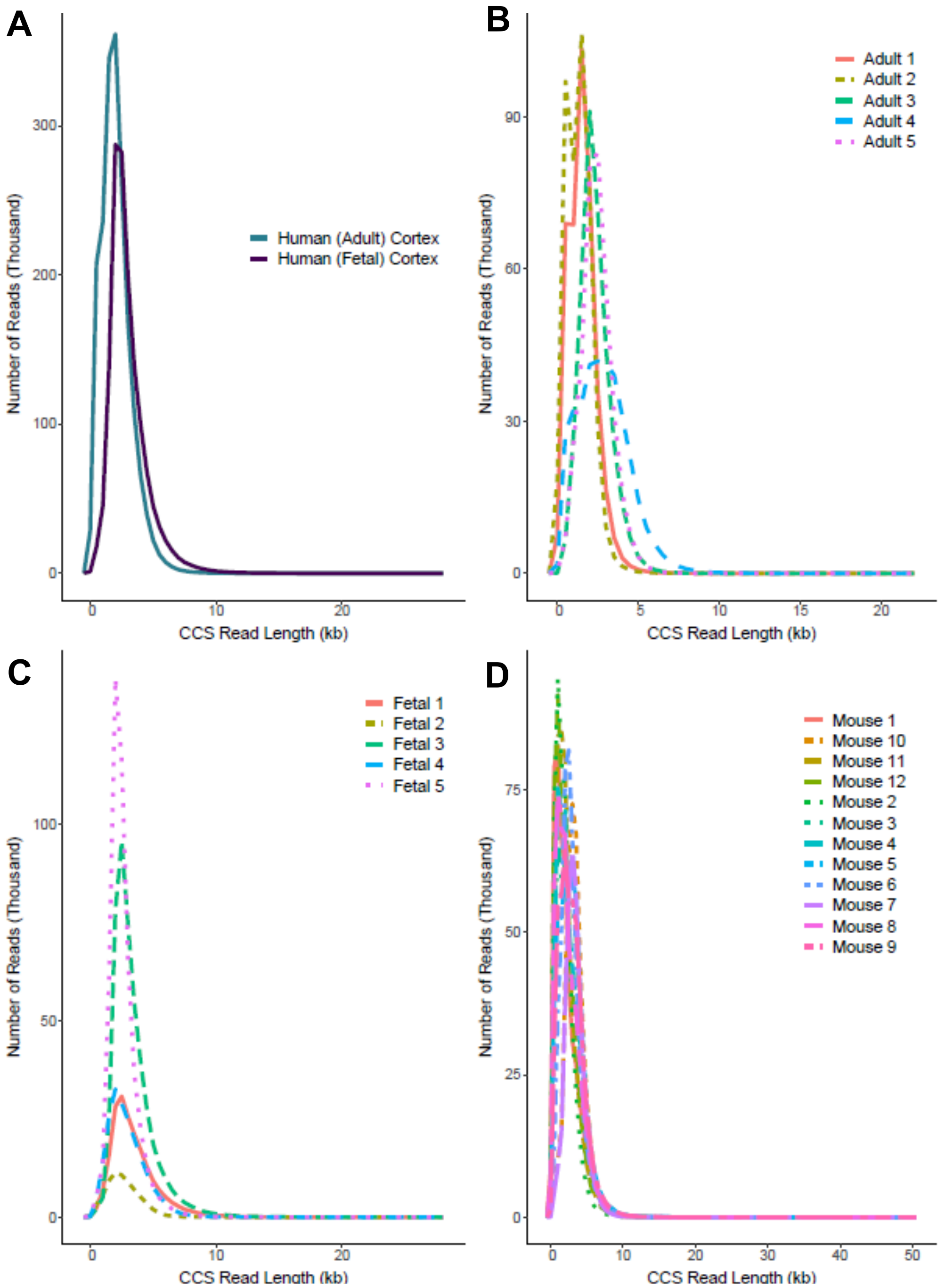

Figure S3: Consensus distribution of CCS read lengths across all cortical samples, related to Figure 1. Shown is data for CCS reads generated from **A)** merging human and adult fetal cortex, and for each sample in **B)** human adult cortex (n = 4 biologically independent samples, n = 5 SMRT cells), **C)** human fetal cortex (n = 3 biologically independent samples, n = 5 SMRT cells) and **D)** mouse cortex (n = 12 biologically independent samples, n = 12 SMRT cells). Several of the human adult and human fetal cortex samples were sequenced more than once to maximize coverage (**Table S1**). Number of CCS reads generated per SMRT cell can be found in **Table S3**. Distribution of CCS read lengths in human and mouse cortex can be found in **Figure 1A**. CCS – Circular consensus sequence. SMRT – Single-molecule real-time

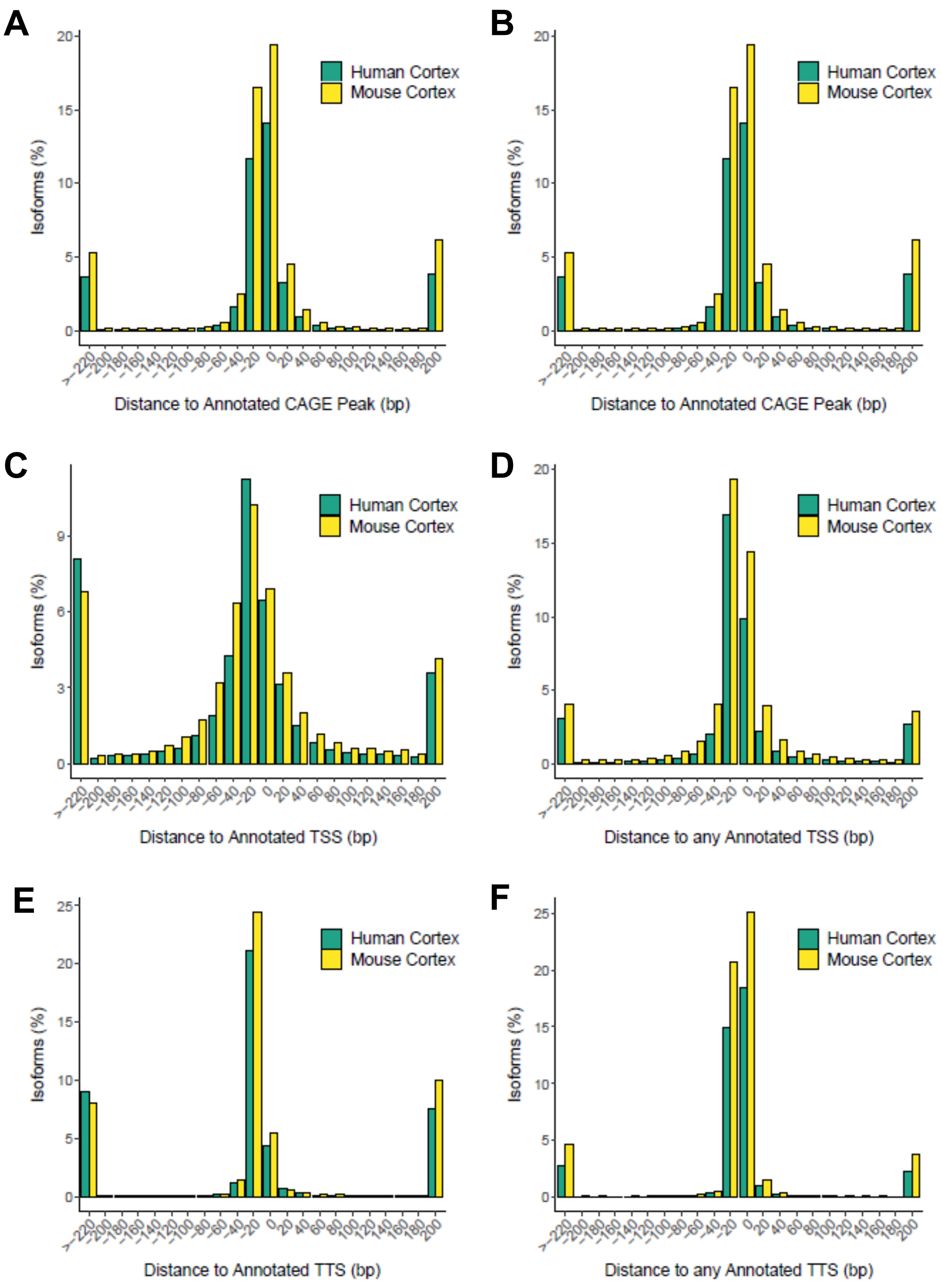

Figure S4: Transcripts identified by Iso-Seq are enriched near CAGE peaks, annotated transcription start sites (TSS) and transcript termination sites (TTS), related to Figure 1. Shown is the distance of transcripts to annotated CAGE peaks for **A)** all and **B)** novel transcripts. Shown also is the distance between the 5' end of each transcript to reference TSSs for **C)** all transcripts and **D)** novel transcripts. Finally, shown also is the distance between the 3' end of each transcript to reference TTSs for **E)** all transcripts and **F)** novel transcripts. A negative value for distance to TSS refers to a query start site downstream of reference, and a negative value for distance to TTS refers to end site upstream of reference. Novel transcripts are classified as NIC, NNC, antisense, genic/genomic, and fusion. TSS – Transcription Start Site, TTS – Transcription Termination Site.

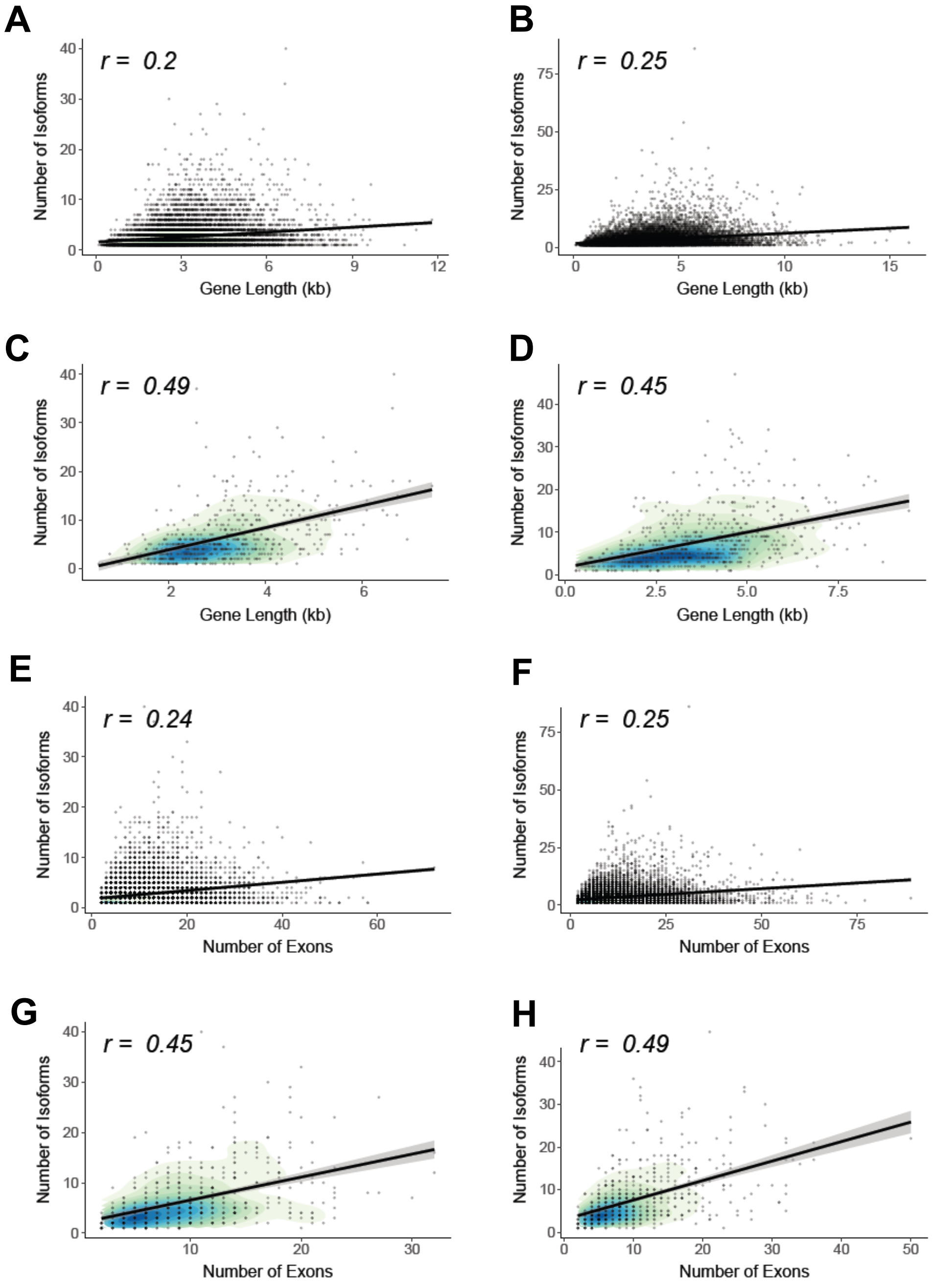

Figure S5: Longer genes and those with more exons tend to have a higher number of discrete isoforms, related to Figure 1. The number of detected multi-exonic isoforms in **A)** human cortex and **B)** mouse cortex is correlated with gene length (human cortex: Pearson's correlation = 0.19, P = 1.51 x 10^{-106}; mouse cortex: Pearson's correlation = 0.25, P = 1.33 x 10^{-197}). A stronger relationship was observed among 'highly-expressed' genes (>2.5 Log_{10} TPM) in both **C)** human cortex (Pearson's correlation = 0.49, P = 1.39 x 10^{-33}) and **D)** mouse cortex (Pearson's correlation = 0.45, P = 3.56 x 10^{-31}). The number of detected isoforms was also correlated with the number of exons in **E)** human cortex (Pearson's correlation = 0.24, P = 7.97 x 10^{-155}) and **F)** mouse cortex (Pearson's correlation = 0.24, P = 4.02 x 10^{-193}). A stronger relationship was observed among 'highly-expressed' genes (>2.5 Log_{10} TPM) in both **G)** human cortex (Pearson's correlation = 0.45, P = 7.42 x 10^{-28}) and **H)** mouse cortex (Pearson's correlation = 0.49, P = 2.16 x 10^{-38}). Gene length corresponds to the longest isoform, and density of genes is represented in increasing scale from light green to dark blue. TPM – Transcripts per Million.

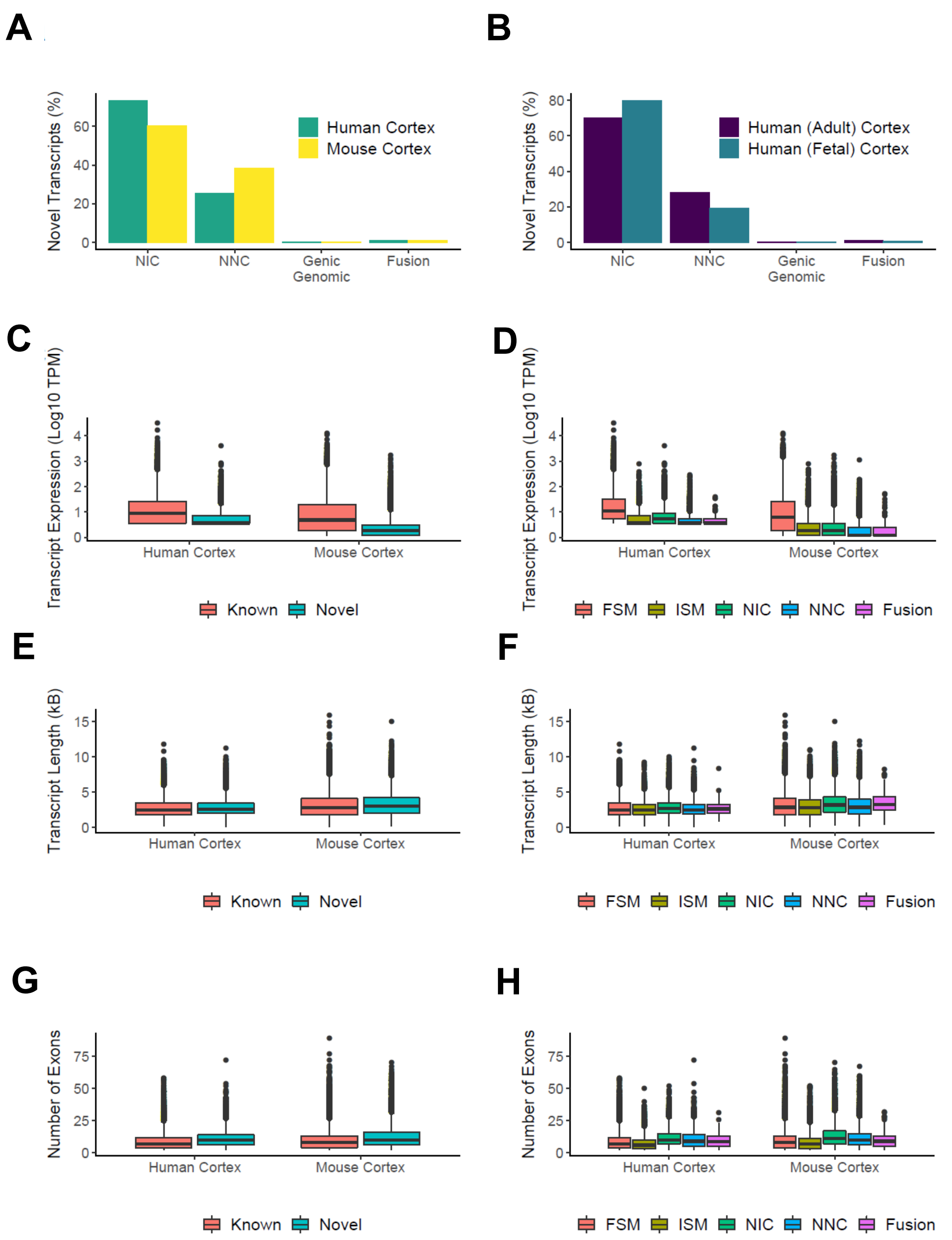

Figure S6: Differences in the abundance and size of novel and known transcripts, related to Figure 2. Shown is the proportion of novel transcripts comparing **A)** human cortex with mouse cortex and **B)** human adult cortex with human fetal cortex. Definitions of the different isoform classifications are shown in **Figure 2A**. **C)** Overall Iso-Seq transcript expression of novel and known transcripts and **D)** different RNA isoform categories. Known transcripts were more highly expressed than novel transcripts in both human (Mann-Whitney-Wilcoxon test, $W = 1.62x\ 10^{8}$, $P < 2.23 \times 10^{-308}$) and mouse cortex (Mann-Whitney-Wilcoxon test, $W = 3.66 \times 10^{8}$, $P < 2.23 \times 10^{-308}$). **E, F)** Transcript length and **G, H)** number of exons for novel and known transcripts of annotated genes, further stratified by RNA isoform category. Novel transcripts were longer (human cortex: Mann-Whitney-Wilcoxon test, $W = 1.10 \times 10^{8}$, $P = 4.04 \times 10^{-25}$; mouse cortex: Mann-Whitney-Wilcoxon test $W = 2.37x\ 10^{8}$, $P = 2.13 \times 10^{-42}$) and had more exons (human cortex: $W = 8.83 \times 10^{7}$, $P < 2.23 \times 10^{-308}$; mouse cortex: $W = 1.94 \times 10^{8}$, $P < 2.23 \times 10^{-308}$) than known transcripts.

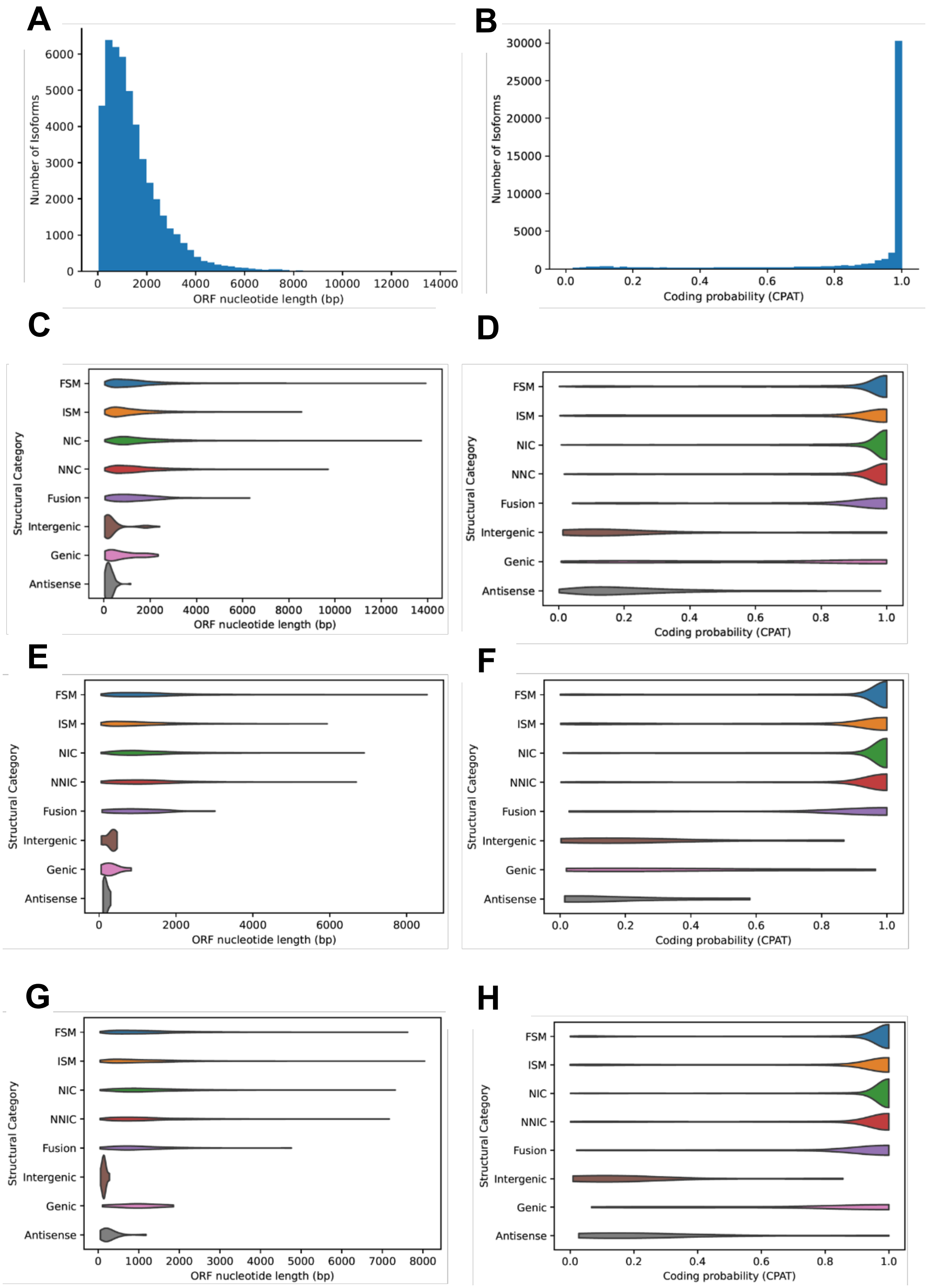

Figure S7: Characterizing open reading frames highlighted a high level of coding potential amongst identified transcripts, related to Figures 1 and 2. Shown is **A)** the distribution of ORF nucleotide length and **B)** CPAT coding probability for all isoforms in mouse cortex (n = 12 biologically independent samples). Also shown is **C)** the distribution of ORF nucleotide length and **D)** CPAT coding probability in mouse cortex stratified by transcript category. The equivalent plots of the distribution of ORF nucleotide length and coding probability are shown for human fetal cortex (**E, F**), human adult cortex (**G, H**) and for human cortex (see **Figure 1C, Figure 1D, Figure 2C, and Figure 2D**).

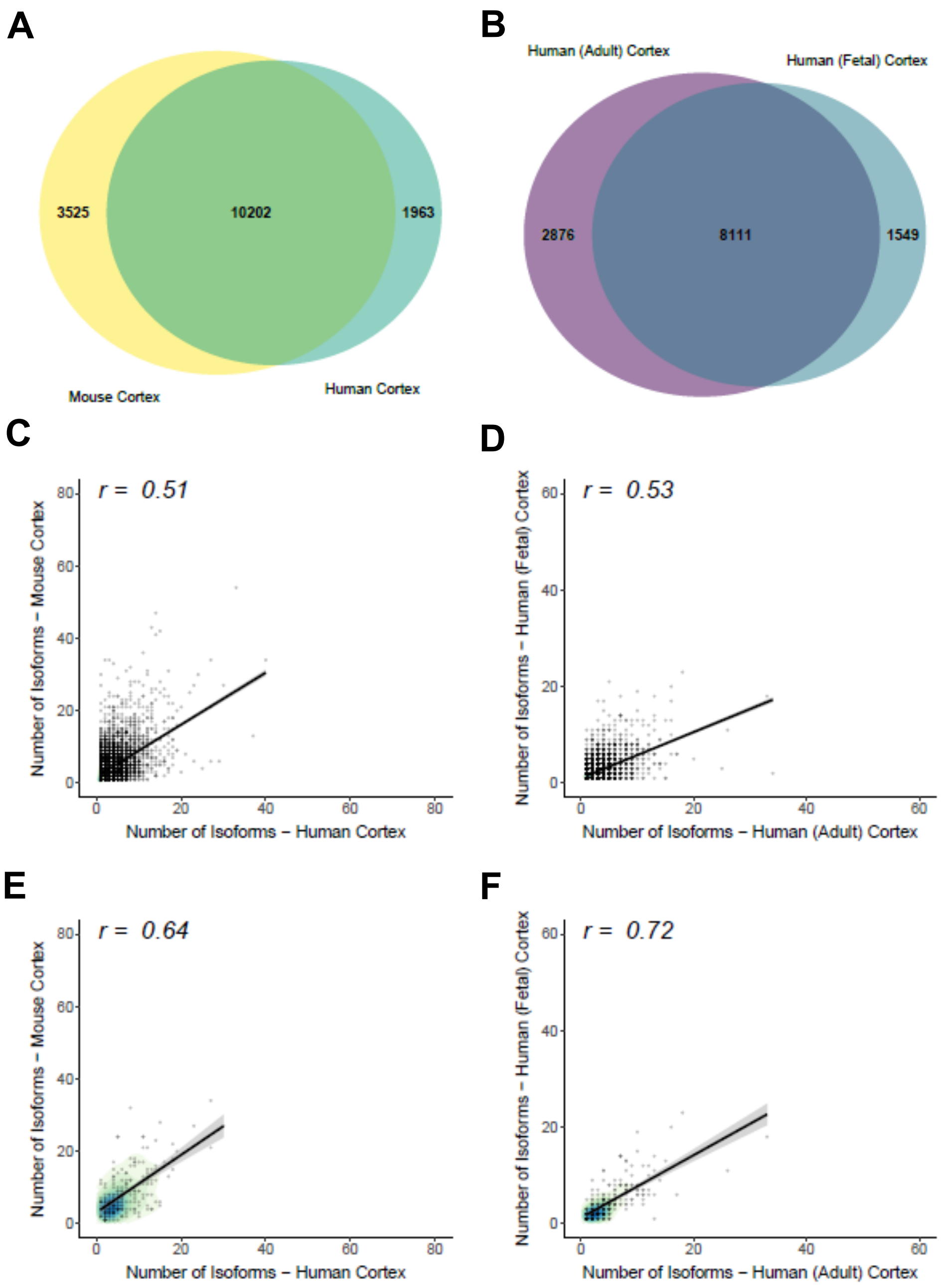

Figure S8: The number of unique isoforms for commonly expressed genes in human and mouse cortex is correlated between species, related to Figure 3. Shown is the number of common and unique annotated genes from GENCODE (human: hg38, mouse: mm10) identified in **A)** a comparison of human and mouse cortex and **B)** a comparison of human adult and human fetal cortex. Of the genes commonly identified, a strong relationship was observed between the number of multi-exonic isoforms in human and mouse cortex across **C)** all transcripts and **D**) in highly-expressed genes (>2.5 Log_{10} TPM). Similarly, a strong relationship was observed between the number of multi-exonic isoforms in human adult and human fetal cortex across **E)** all transcripts and **F)** in highly-expressed genes (>2.5 Log_{10} TPM).

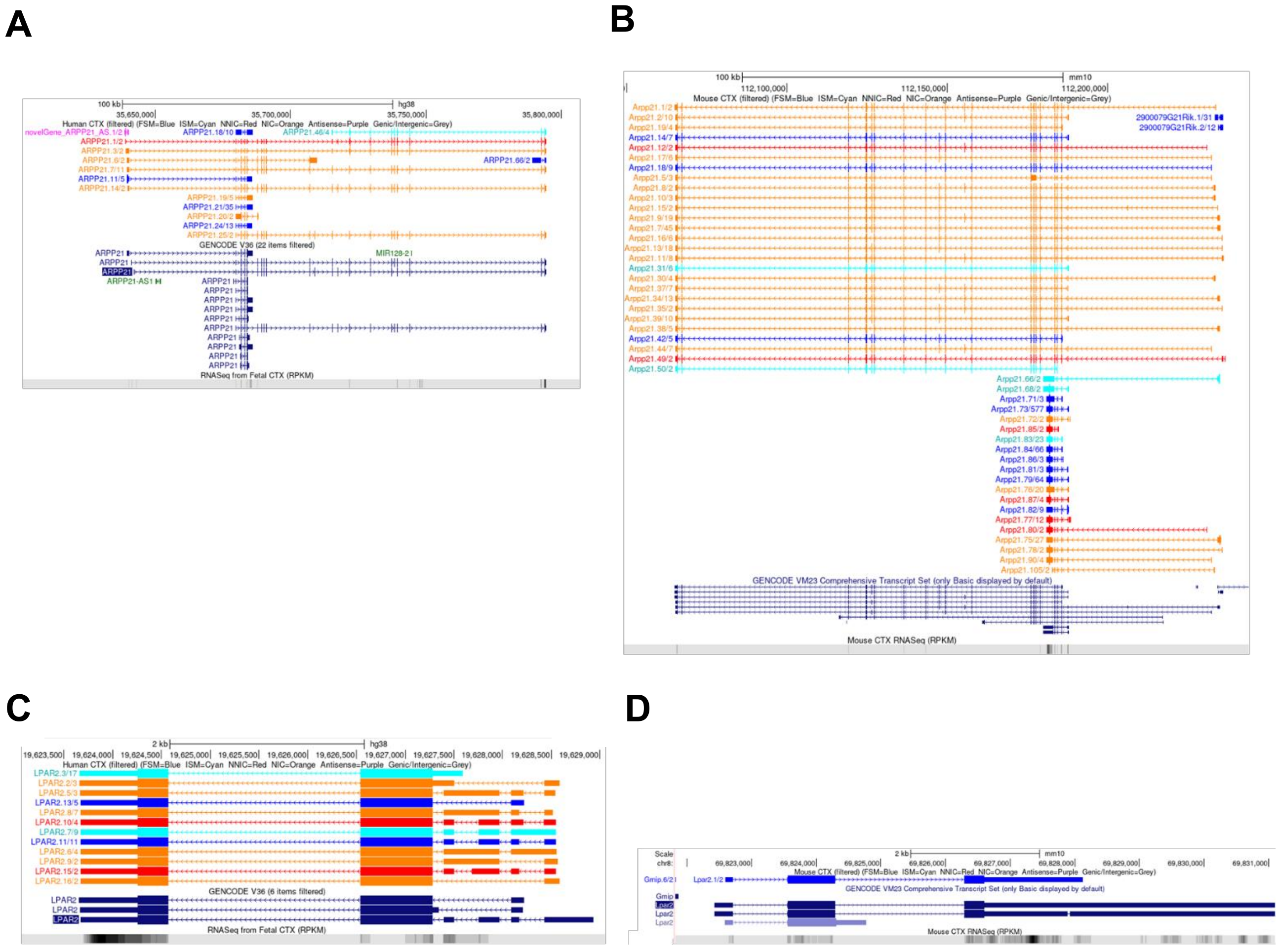

Figure S9: Notable difference in isoform number in *ARPP21 /Arpp21* and *LPAR2 /Lpar2* gene between the human and mouse cortex, related to Figure 3. Shown is a UCSC genome browser track of **A)** the *ARPP21* gene in human cortex (n = 14 multi-exonic isoforms) and **B)** the *Arpp21* gene in mouse cortex (n = 47 multi-exonic isoform). Also shown is a UCSC genome browser track of **C)** the *LPAR2* gene in human cortex (n = 12 multi-exonic isoforms) and **D)** the *Lpar2* gene in mouse cortex (n = 1 multi-exonic isoform). Isoforms are coloured based on *SQANTI2* classification categories (blue = FSM, cyan = ISM, red = NNC, orange = NIC). FSM – Full Splice Match, ISM – Incomplete Splice Match, NIC – Novel In Catalogue, NNC – Novel Not in Catalogue

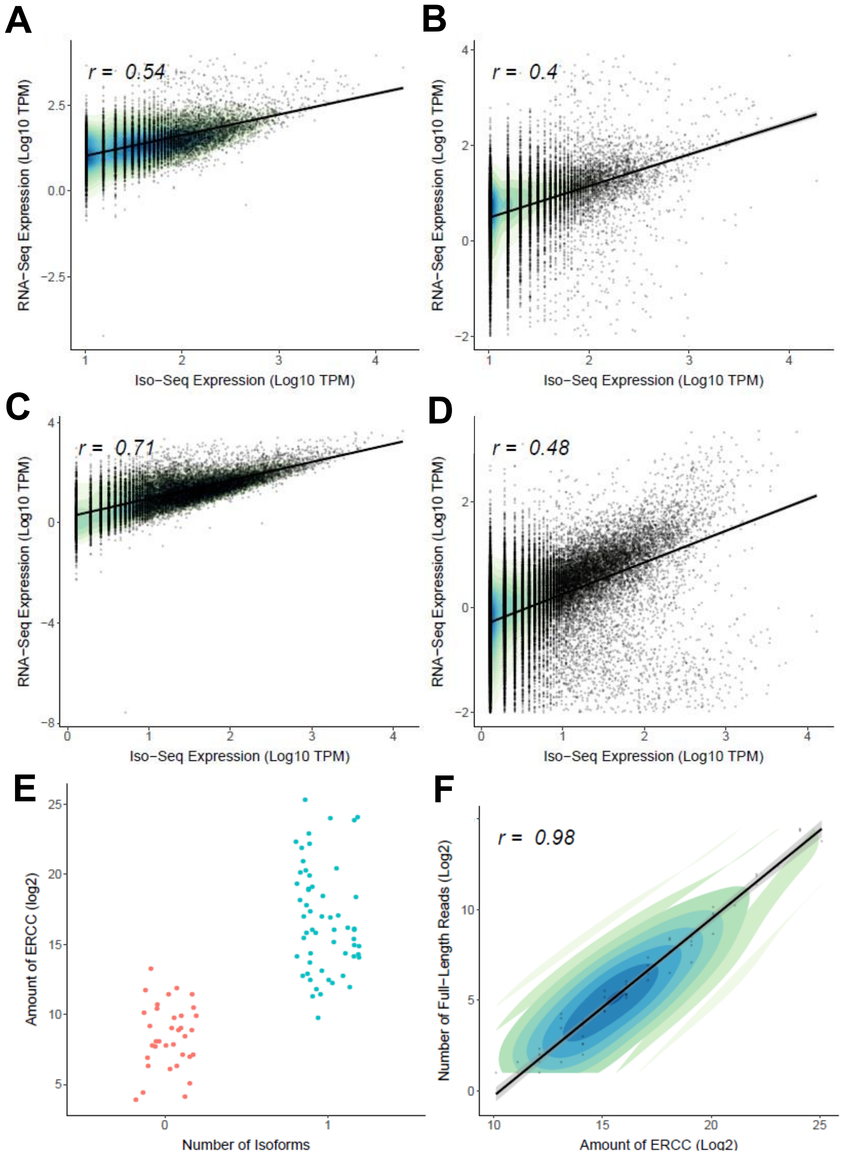

Figure S10: Long-read Iso-Seq data can be used to accurately quantify levels of gene expression in the human cortex, related to STAR Methods. Shown is the relationship between expression estimated using RNA-Seq and Iso-Seq at the **A)** gene level (n = 9,223 genes, Pearson's correlation = 0.58, $P < 2.23 \times 10^{-308}$) and **B)** transcript level (n = 17,583 transcripts, corr = 0.40, $P < 2.23 \times 10^{-308}$) in the human cortex (data derived from three biologically independent fetal samples). Also shown is the same relationship at the **C)** gene level (n = 13,923 genes, corr = 0.71; $P < 2.23 \times 10^{-308}$) and **D)** transcript level (n = 41,488 transcripts, corr = 0.48, $P < 2.23 \times 10^{-308}$) for mouse cortex (n = 12 biologically independent samples). RNA-Seq gene expression was determined after aligning short-read RNA-Seq to the Iso-Seq transcriptome. Iso-Seq gene expression was determined from the sum of full-length, multi-exonic transcript reads associated for each gene, with TPM values calculated by dividing the number of full length reads per gene by total full-length reads, multiplied by a million. The density of values is represented in increasing scale from light green to dark blue. **E)** The number of ERCC spike-in fragments detected compared to the amount used in our mouse cortex Iso-Seq libraries and **F)** the relationship between the amount of ERCC used and the number of full-length reads identified (Pearson's correlation = 0.98, $P = 1.42 \times 10^{-41}$). There is a near perfect correlation between full-length reads associated with ERCC spike-in fragments and the actual amount of control used.

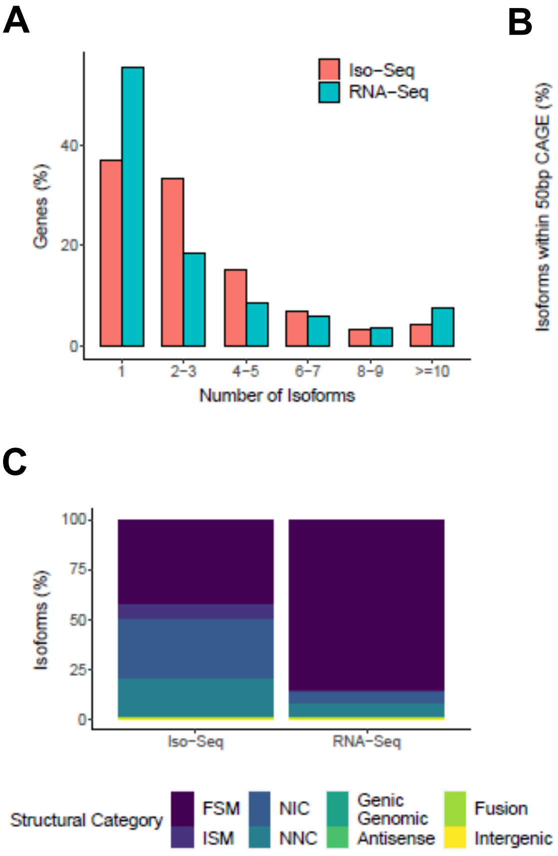

Figure S11: Transcripts from an RNA-Seq defined transcriptome were less supported by CAGE peaks, and likely represent incomplete fragments of transcripts identified using Iso-Seq reads, related to STAR Methods. Shown is a comparison of the **A)** distribution of number of isoforms associated per gene, **B)** the proportion of isoforms annotated within 50bp of a CAGE peak, and **C)** classification of the isoforms using *SQANTI* categories, between Iso-Seq defined and RNA-Seq defined transcriptomes generated on the mouse cortex. RNA-Seq defined transcriptome is generated using a reference-guided assembly of RNA-Seq reads (n = 12 biologically independent samples) using *Stringtie.*

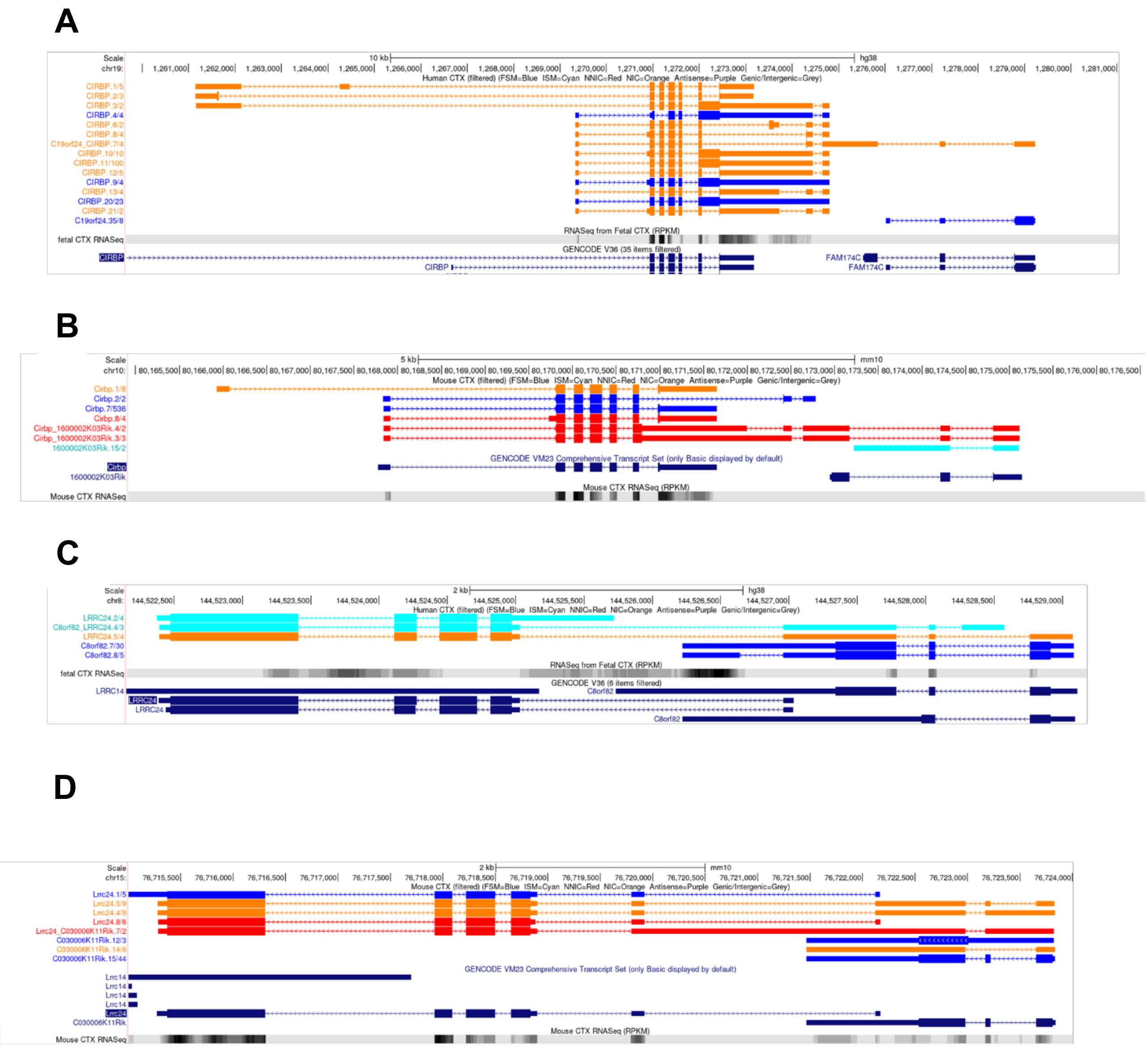

Figure S12: Additional examples of similar fusion transcripts observed in both human and mouse cortex, related to Figure 4. Shown are UCSC tracks of **A)** the *CIRBP-FAM174C* gene in the human cortex and **B)** the *Cirbp-1600002K03RIK* gene in the mouse cortex. Also shown are UCSC tracks of **C)** the *C8orf82-LRCC24* gene in the human cortex and **D)** the *Lrcc24-C030006K11Rik* gene in the mouse cortex. Isoforms are coloured based on *SQANTI2* classification categories (blue = FSM, cyan = ISM, red = NNC, orange = NIC). FSM – Full Splice Match, ISM – Incomplete Splice Match, NIC – Novel In Catalogue, NNC – Novel Not in Catalogue

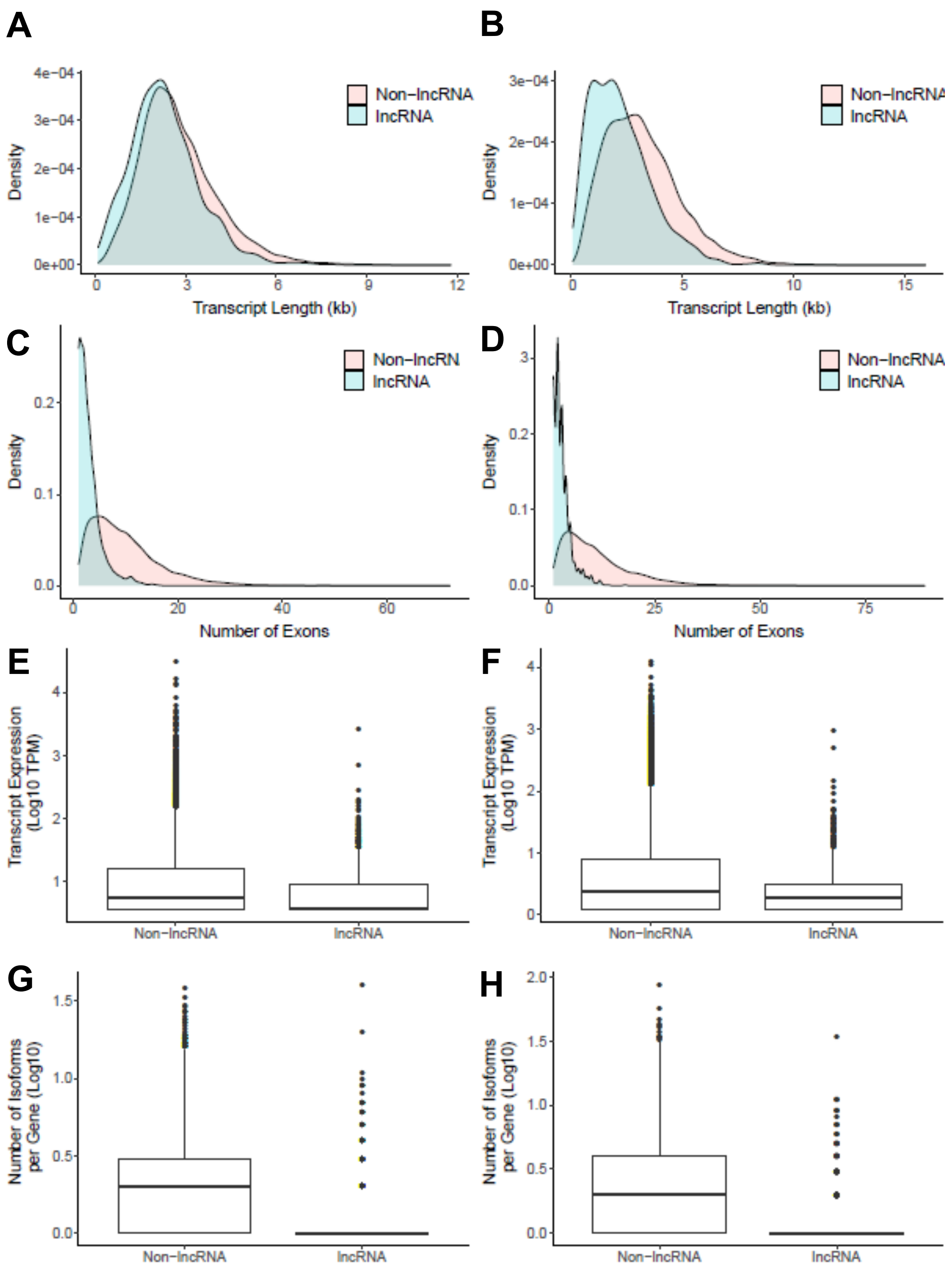

Figure S13: lncRNA transcripts are typically shorter, contain fewer exons, are less abundant and have fewer isoforms than non-lncRNA transcripts, related to Table 1. LncRNA transcripts were found to be shorter in both **A)** human cortex (Mann-Whitney-Wilcoxon, W = 2.28 x 10^{7}, P = 3.22 x 10^{-34}) and **B)** mouse cortex (Mann-Whitney-Wilcoxon test, 3.52 x 10^{7}, P = 8.24 x 10^{-98}). They also contained fewer exons in both **C)** human cortex (Mann-Whitney-Wilcoxon test, W = 3.31 x 10^{7}, P < 2.23 x 10^{-308}) and **D)** mouse cortex (Mann-Whitney-Wilcoxon test, W = W = 4.56 x 10^{7}, P < 2.23 x 10^{-308}). They were also characterized by lower transcript expression than non-lncRNA transcripts in **E)** human (Mann-Whitney-Wilcoxon test, W = 2.27 x 10^{7}, P = 9.44 x 10^{-35}) and **F)** mouse cortex (Mann-Whitney-Wilcoxon test, W = 3.16 x 10^{7}, P = 5.67 x 10^{-40}). Finally, they showed lower isoform diversity in **G)** human (Mann-Whitney-Wilcoxon test, W = 6.63 x 10^{6}, P = 1.21 x 10^{-80}) and **H)** mouse cortex (Mann-Whitney-Wilcoxon test, W = 7.40 x 10^{6}, P = 5.76 x 10^{-107}). Lnc-RNA – long non-coding RNA.

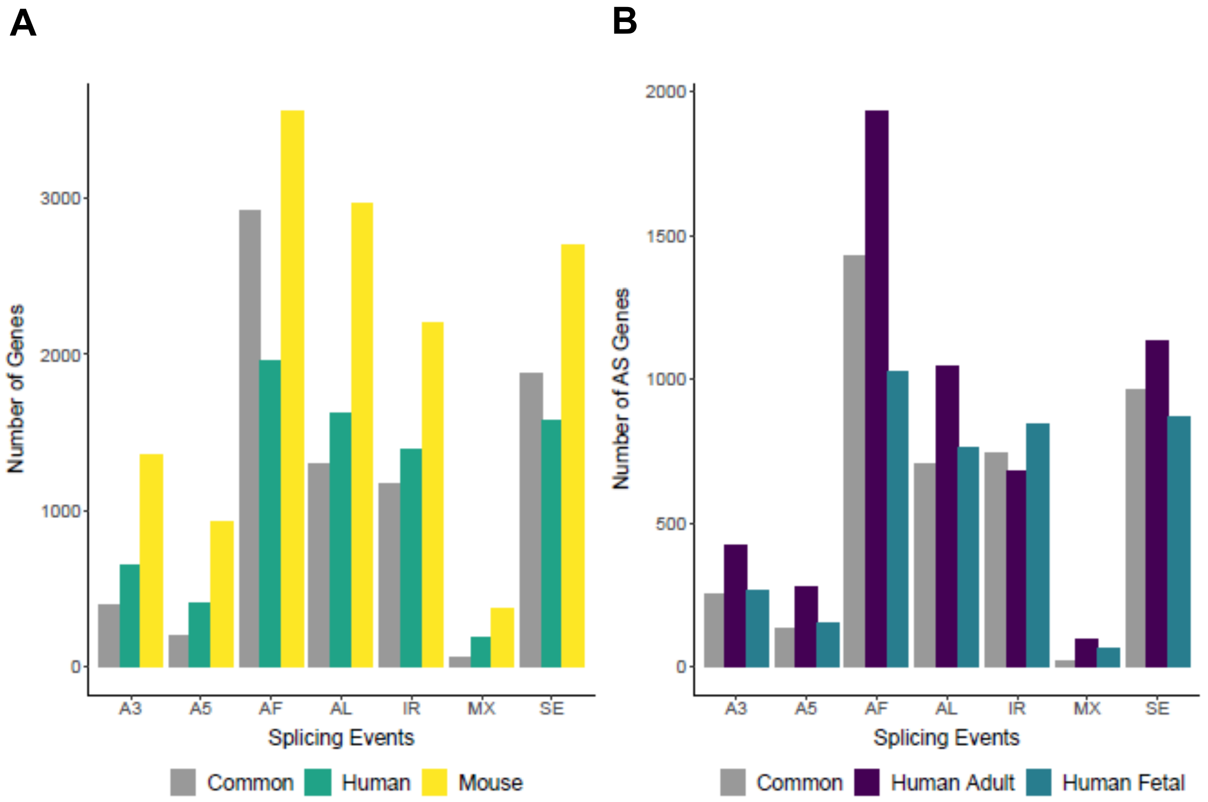

Figure S14: Alternative first exon and alternative last exon are the most prominent AS events in human and mouse cortex, related to Figure 5. There was also a large overlap of genes influenced by specific AS events in human and mouse cortex, as shown by number of genes commonly observed with the different AS events (A3, A5, AF, AL, IR, MX, and SE) between **A)** human and mouse cortex, and between **B)** human adult and human fetal cortex. MX and SE events were determined using *SUPPA2*, IR with *SQANTI2* and A3', A5', AF and AL with custom scripts. AF – Alternative First Exon, AL – Alternative Last Exon, A5' – Alternative 5' prime, A3' – Alternative 3' prime, IR – Intron Retention, MX – Mutually Exclusive, SE – Skipped Exon. Of note, genes observed with individual AS events are not mutually exclusive.

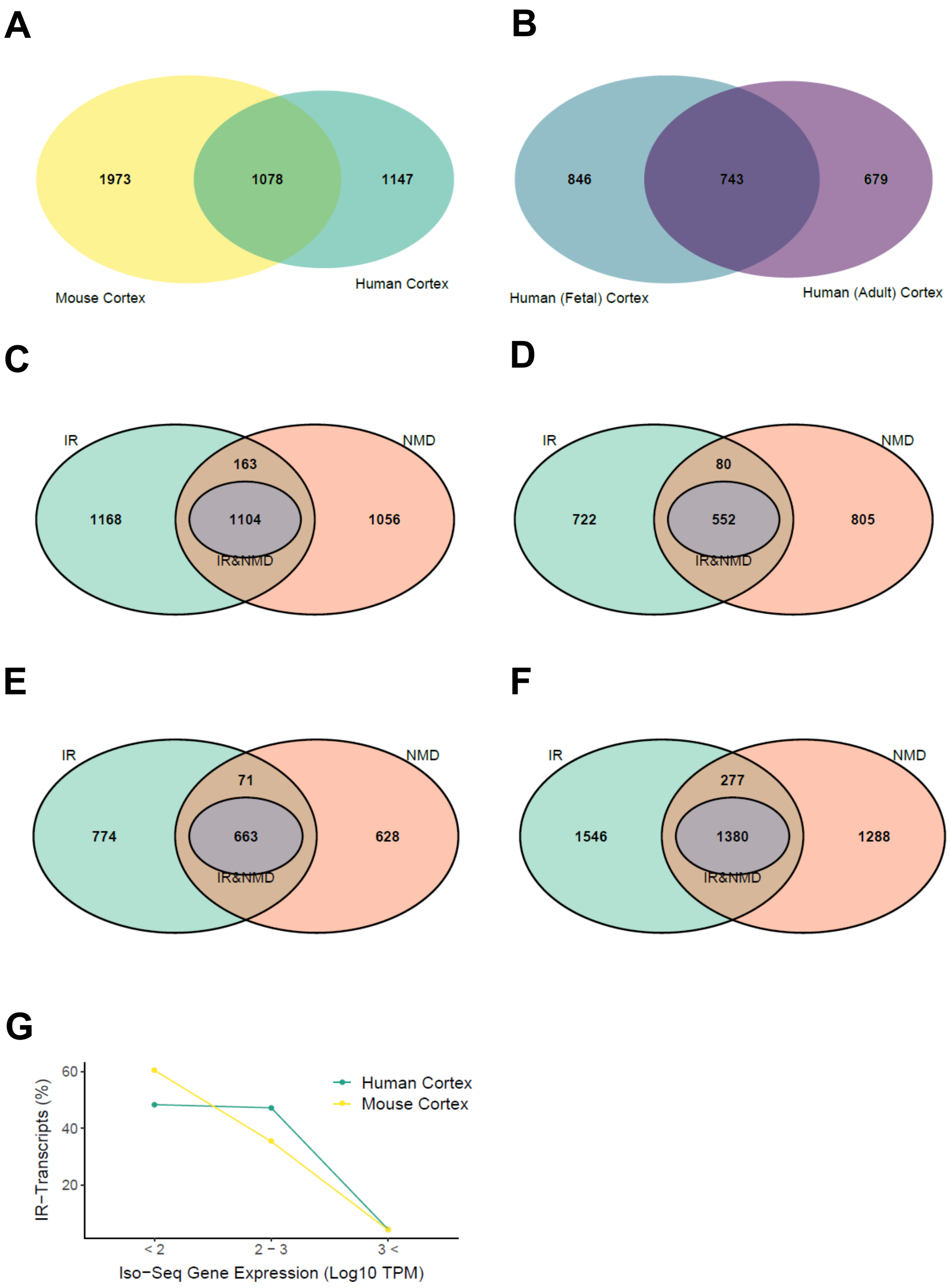

Figure S15: Intron retention and NMD in the human and mouse cortex, related to Figure 5. Shown is the number of genes with intron-retained transcripts comparing A**)** human and mouse cortex and **B)** human adult and human fetal cortex. NMD is particularly enriched amongst transcripts with intron retention**,** as shown by the overlap of genes with IR-transcripts, NMD- transcripts, and transcripts with both IR and NMD in **C)** human cortex, **D)** human adult cortex, **E)** human fetal cortex and **F)** mouse cortex. Genes containing both IR and NMD transcripts were further classified into genes that contain transcripts that were *both* IR and NMD (purple) and genes that contain transcripts where IR and NMD were mutually exclusive (dark orange). **G)** A larger proportion of lowly expressed genes showed evidence for IR than highly expressed genes in both human (< 2.5 Log_{10} TPM, n = 2,269 (88.4%) genes; > 2.5 Log_{10} TPM, n = 297 (11.6%) genes) and mouse (< 2.5 Log_{10} TPM, n = 3,039 (90.04%) genes; > 2.5 Log_{10} TPM, n = 336 (9.96%) genes. IR – Intron retention. NMD – Nonsense-mediated mRNA decay. TPM – Transcripts per Million

A

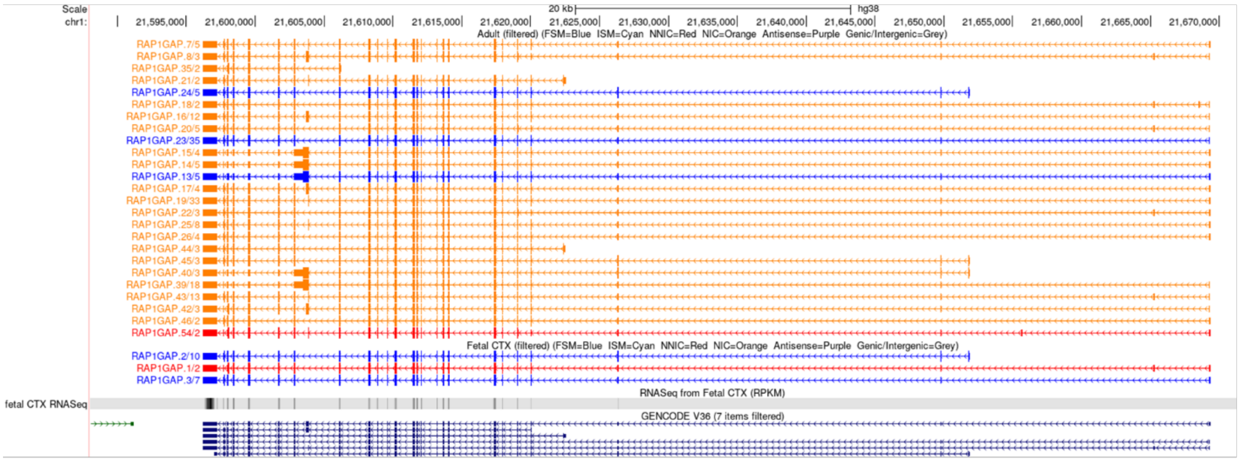

B

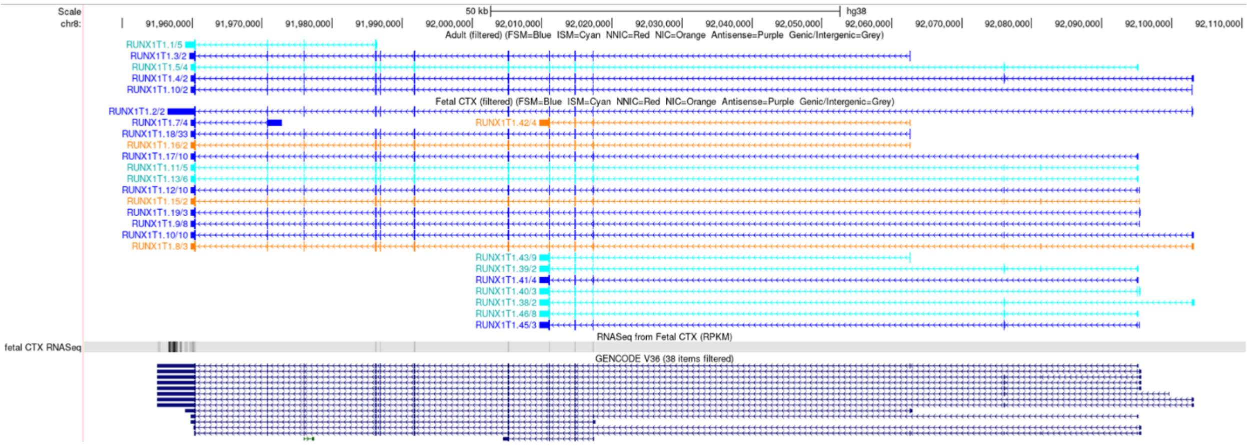

Figure S16: Notable differences in isoform number among transcripts of *RAP1GAP* and *RUNX1T1* between human adult and fetal cortex, related to Figure 3. Shown is a UCSC genome browser track of **A)** *RAP1GAP* and **B)** *RUNX1T1* in human adult and human fetal cortex. These genes are characterized by a large absolute difference in isoform numbers detected between human fetal and human adult cortex: *RAP1GAP* (adult cortex: n = 25 isoforms; fetal cortex, n = 3 isoforms) and *RUNX1T1* (adult cortex: n = 5 isoforms; fetal cortex: n = 21 isoforms). Isoforms are coloured based on *SQANTI2* classification categories (blue = FSM, cyan = ISM, red = NNC, orange = NIC). FSM – Full Splice Match, ISM – Incomplete Splice Match, NIC – Novel In Catalogue, NNC – Novel Not in Catalogue

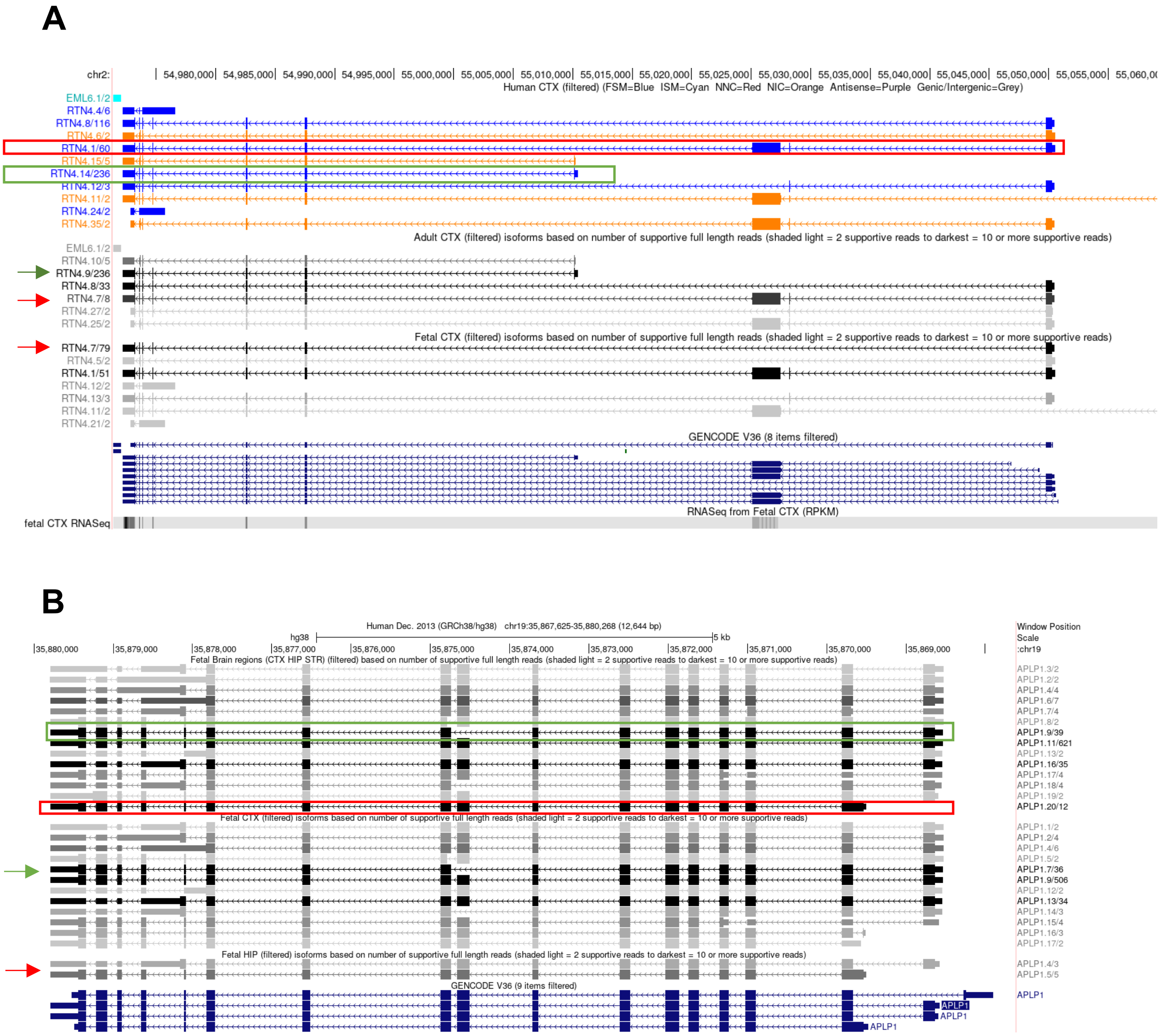

Figure S17: Differential transcript usage between human adult and fetal cortex for *RTN4* and *APLP1,* related to Figures 3 and 5. Shown are UCSC genome browser tracks for **A)** *RTN4* transcripts in human cortex, coloured by transcript classification, and **B)** *APLP1* transcripts in human fetal brain regions, shaded by the number of full-length reads. Differential transcript usage was observed in *RTN4*, with one isoform (boxed in red – RTN4.1, red arrow) strongly expressed in fetal cortex while downregulated in adult cortex, and another isoform (boxed in green – RTN4.14, green arrow) strongly expressed only in adult cortex. Differential transcript usage was observed in *APLP1* with one transcript (boxed in red – APLP1.20, ENST00000586861.5) strongly expressed in fetal hippocampus (red arrow) while not detected in fetal cortex, and another novel isoform (boxed in green – APLP1.9) strongly expressed in fetal cortex (green arrow).

Sample ID	Number of SMRT Cells	Species	Tissue	Sex	Age	RIN	RNA-Seq (Illumina)	Nanopore sequencing (ONT)
Adult A	1	Human	Cortex	F	89years	4.1	No	No
Adult B	1	Human	Cortex	F	89years	6.3	No	No
Adult C	2	Human	Cortex	M	24years	8	No	Yes
Adult D	1	Human	Cortex	M	45years	7	No	No
Fetal A	1	Human	Cortex	M	17wpc	8.3	Yes	Yes
Fetal B	2	Human	Cortex	F	17wpc	7.2	Yes	No
Fetal C	2	Human	Cortex	F	14wpc	6.1	Yes	No
Fetal D	1	Human	Hippocampus	F	17wpc	7.5	No	No
Fetal E	1	Human	Hippocampus	F	14wpc	7.1	No	No
Fetal F	1	Human	Striatum	F	17wpc	8.1	No	No
Fetal G	1	Human	Striatum	F	14wpc	6.9	No	No
Mouse A	1	Mouse	Cortex	F	2months	9.2	Yes	No
Mouse B	1	Mouse	Cortex	F	2months	8.8	Yes	No
Mouse C	1	Mouse	Cortex	F	8months	9.1	Yes	No
Mouse D	1	Mouse	Cortex	F	8months	9.2	Yes	No
Mouse E	1	Mouse	Cortex	F	8months	8.7	Yes	No
Mouse F	1	Mouse	Cortex	F	2months	9.2	Yes	No
Mouse G	1	Mouse	Cortex	F	2months	8.9	Yes	No
Mouse H	1	Mouse	Cortex	F	8months	9	Yes	No
Mouse I	1	Mouse	Cortex	F	8months	8.6	Yes	No
Mouse J	1	Mouse	Cortex	F	2months	9.2	Yes	No
Mouse K	1	Mouse	Cortex	F	2months	8.9	Yes	No
Mouse L	1	Mouse	Cortex	F	8months	9.1	Yes	No

Table S1: Description of the individual samples included in this study, related to STAR Methods. Of note fetal hippocampus and striatum samples were derived from the same donor as the fetal cortex samples. SMRT – Single-molecule real-time, ONT – Oxford Nanopore Technology, RIN – RNA Integrity Number, wpc – weeks post-conception

Run ID	Sample ID	Species	Tissue	Number of CCS generated
Adult 1	Adult A	Human	Cortex	388960
Adult 2	Adult B	Human	Cortex	420057
Adult 3	Adult C	Human	Cortex	352900
Adult 4	Adult C	Human	Cortex	351182
Adult 5	Adult D	Human	Cortex	356361
Fetal 1	Fetal A	Human	Cortex	196123
Fetal 2	Fetal B	Human	Cortex	69247
Fetal 3	Fetal B	Human	Cortex	471115
Fetal 4	Fetal C	Human	Cortex	199170
Fetal 5	Fetal C	Human	Cortex	493077
Fetal 6	Fetal D	Human	Hippocampus	214602
Fetal 7	Fetal E	Human	Hippocampus	206790
Fetal 8	Fetal F	Human	Striatum	268602
Fetal 9	Fetal G	Human	Striatum	202211
Mouse 1	Mouse A	Mouse	Cortex	470079
Mouse 2	Mouse B	Mouse	Cortex	464504
Mouse 3	Mouse C	Mouse	Cortex	503595
Mouse 4	Mouse D	Mouse	Cortex	509175
Mouse 5	Mouse E	Mouse	Cortex	511698
Mouse 6	Mouse F	Mouse	Cortex	461026
Mouse 7	Mouse G	Mouse	Cortex	353481
Mouse 8	Mouse H	Mouse	Cortex	493532
Mouse 9	Mouse I	Mouse	Cortex	498732
Mouse 10	Mouse J	Mouse	Cortex	409403
Mouse 11	Mouse K	Mouse	Cortex	486693
Mouse 12	Mouse L	Mouse	Cortex	495762

Table S3: Number of successful CCS reads generated across samples included in this study, related to Figure 1. Distribution of CCS read lengths across all cortical samples can be found in **Figures S3A-S3D.** CCS - Circular consensus sequence

		Human Cortex	Human Cortex		Mouse Cortex
			Adult	Fetal	
	Unique Genes	12964	11021	9679	14684
	Annotated Genes	12910 (99.58%)	10987 (99.69%)	9660 (99.8%)	14482 (98.62%)
	Novel Genes	54 (0.42%)	34 (0.31%)	19 (0.2%)	202 (1.38%)
Annotated Genes	Transcripts	32745	22013	18592	46403
	Protein-coding Transcripts	30388(92.8%)	20525(93.24%)	17456(93.89%)	43450(93.64%)
	Genes associated with coding transcripts	12087	10315	9198	13710
	Non-lncRNA Transcripts	31548	21293	18054	45262
	lncRNA Transcripts	1197	720	538	1141
	Mono-exonic non lncRNA	583(1.85%)	440(2.07%)	365(2.02%)	914(2.02%)
	Mono-exonic lncRNA Transcripts	348(29.07%)	232(32.22%)	174(32.34%)	273(23.93%)
	Genes associated with lncRNA Transcripts	792	535	365	734
	Protein-coding lncRNA Transcripts	521(43.53%)	310(43.06%)	233(43.31%)	448(39.26%)
	Annotated Transcripts (FSM, ISM)	20832 (63.62%)	15659 (71.14%)	13177 (70.87%)	23530 (50.71%)
	Novel Transcripts	11913 (36.38%)	6354 (28.86%)	5415 (29.13%)	22873 (49.29%)
	FSM	17080 (52.16%)	13007 (59.09%)	11346 (61.03%)	19803 (42.68%)
	ISM	3752 (11.46%)	2652 (12.05%)	1831 (9.85%)	3727 (8.03%)
	NIC	8721 (26.63%)	4464 (20.28%)	4315 (23.21%)	13763 (29.66%)
	NNC	3021 (9.23%)	1796 (8.16%)	1041 (5.6%)	8751 (18.86%)
	Genic Genomic	35 (0.11%)	20 (0.09%)	8 (0.04%)	62 (0.13%)
	Antisense	0 (0%)	0 (0%)	0 (0%)	0 (0%)
	Fusion	136 (0.42%)	74 (0.34%)	51 (0.27%)	297 (0.64%)
	Intergenic	0 (0%)	0 (0%)	0 (0%)	0 (0%)
	Genic Intron	0 (0%)	0 (0%)	0 (0%)	0 (0%)
	Genes associated with Novel Transcripts	5327 (41.26%)	3468 (31.56%)	3027 (31.34%)	8054 (55.61%)
	Genes associated with Annotated Transcripts	12137 (94.01%)	10350 (94.2%)	9079 (93.99%)	13551 (93.57%)
	NMD Transcripts	4370 (13.35%)	2337 (10.62%)	2222 (11.95%)	6014 (12.96%)
	Genes with NMD transcripts	2323 (17.99%)	1437 (13.08%)	1362 (14.1%)	2945 (20.34%)

Fusion Genes	108 (0.84%)	45 (0.41%)	58 (0.6%)	218 (1.51%)
Transcripts of Fusion Genes	136 (0.42%)	51 (0.23%)	74 (0.4%)	297 (0.64%)
Fusion genes with more than one transcript	22 (20.37%)	5 (11.11%)	10 (17.24%)	53 (24.31%)
Transcripts with Intron Retention	5231 (15.95%)	2383 (10.81%)	2783 (14.95%)	6803 (14.59%)
Genes with Intron Retention	2566 (19.79%)	1422 (12.9%)	1589 (16.42%)	3375 (22.98%)
Protein-coding, IR-transcripts	4983 (95.26%)	2281 (95.72%)	2649 (95.19%)	6453 (94.86%)
IR-transcripts with canonical splice junctions	5231 (100%)	2383 (100%)	2760 (99.17%)	6766 (99.46%)

Table S6: Overview of the human and mouse cortical transcriptomes characterized using Iso-Seq, related to Table 1.
lncRNA – long non-coding RNA, FSM – Full Splice Match, ISM – Incomplete Splice Match, NIC – Novel In Catalogue, NNC – Novel Not in Catalogue, NMD – Nonsense-mediated mRNA decay

Gene	Iso-Seq Dataset	Peptide	PB.ID	Event
RGS11	Adult Cortex	GAELAGIGVGLR	PB.7368.23	Intron Retention
ARHGEF11	Adult Cortex	SSSQSIKPGNVR	PB.1416.6	Exon Skipping
VTI1A	Fetal Cortex	NELLGDDGNSSENQ LIK	PB.2147.4	Novel Exon Inclusion
RELCH	Fetal Cortex	VAEHEVPLQER	PB.7004.2	Exon Skipping
EPB41L1	Fetal Cortex	SLSPTVK	PB.9417.2	Exon Skipping

Table S8: Examples of proteomic support for novel transcripts identified using Iso-Seq, related to Figures 2 and 5. We identified five novel peptides, each mapping uniquely to a single novel transcript, providing evidence for the stable translation of novel isoforms in the human cortex. PB.ID refers to the specific Iso-Seq transcript supported. Peptide support for novel exon inclusion in *VTI1A* is illustrated in **Figure 2E**, intron retention in *RGS11* in **Figure 5F**, and novel exon skipping in *RELCH* in **Figure 5G**.

A)

Splicing event	Number and proportion of splicing events			
	Human Cortex	Human (Adult) Cortex	Human (Fetal) Cortex	Mouse Cortex
A3	1707 (6.49%)	1057 (7.15%)	737 (6.16%)	3217 (7.99%)
A5	915 (3.48%)	561 (3.79%)	380 (3.18%)	1883 (4.68%)
AF	8456 (32.16%)	5364 (36.26%)	3504 (29.31%)	12853 (31.93%)
AL	3883 (14.77%)	2163 (14.62%)	1800 (15.06%)	6182 (15.36%)
IR	5231 (19.89%)	2383 (16.11%)	2783 (23.28%)	6803 (16.9%)
MX	328 (1.25%)	142 (0.96%)	109 (0.91%)	625 (1.55%)
SE	5776 (21.97%)	3123 (21.11%)	2642 (22.1%)	8686 (21.58%)

B)

Splicing event	Number and proportion of genes with splicing events			
	Human Cortex	Human (Adult) Cortex	Human (Fetal) Cortex	Mouse Cortex
A3	1045 (8.06%)	677 (6.14%)	521 (5.38%)	1754 (11.94%)
A5	604 (4.66%)	408 (3.7%)	281 (2.9%)	1134 (7.72%)
AF	4879 (37.63%)	3357 (30.46%)	2451 (25.32%)	6476 (44.1%)
AL	2920 (22.52%)	1749 (15.87%)	1469 (15.18%)	4262 (29.02%)
IR	2566 (19.79%)	1422 (12.9%)	1589 (16.42%)	3375 (22.98%)
MX	256 (1.97%)	116 (1.05%)	88 (0.91%)	434 (2.96%)
SE	3446 (26.58%)	2093 (18.99%)	1830 (18.91%)	4570 (31.12%)

Table S12: Alternative splicing events observed in human and mouse cortex, related to Figure 5. Tabulated are the **A)** number of splicing events and B**)** number of genes observed with those splicing event, in human cortex (n = 7 biologically independent samples), human adult cortex (n = 4 biologically independent samples), human fetal cortex (n = 3 biologically independent samples), and mouse cortex (n = 12 biologically independent samples). Of note, a single gene can be characterised by multiple splicing events and can thus appear more than once in **B)**. The percentage refer to the proportion of total number of detected genes. A combination of the *SUPPA2* package and custom analysis scripts were used to identify transcripts associated with i) exon skipping (SE), ii) mutually exclusive exon use (MX), iii) alternative first (AF) and last (AL) exons, iv) alternative 3' and 5' splice sites, and v) intron retention (IR).

Description	Fetal Hippocampus	Fetal Striatum
Unique Genes	5568	5974
Annotated Genes	5566 (99.96%)	5969 (99.92%)
Novel Genes	2 (0.04%)	5 (0.08%)
Transcripts	7253	8150
Annotated Transcripts	6250 (86.17%)	6873 (84.33%)
Novel Transcripts	1003 (13.83%)	1277 (15.67%)
FSM	5774 (79.61%)	6226 (76.39%)
ISM	476 (6.56%)	647 (7.94%)
NIC	839 (11.57%)	1040 (12.76%)
NNC	155 (2.14%)	218 (2.67%)
Genic Genomic	0 (0%)	6 (0.07%)
Antisense	1 (0.01%)	2 (0.02%)
Fusion	7 (0.1%)	8 (0.1%)
Intergenic	1 (0.01%)	3 (0.04%)
Genic Intron	5568	5974

Table S17: Summary of the fetal hippocampus and fetal striatum Iso-Seq datasets, related to Table 1. FSM – Full Splice Match, ISM – Incomplete Splice Match, NIC – Novel In Catalogue, NNC – Novel Not in Catalogue

Description	Human Cortex			Mouse Cortex		
	AD	SZ	Autism	AD	SZ	Autism
Disease-associated genes	62	339	393	62	339	393
Detected disease-associated genes ("Detected")	33	288	317	41	318	342
Total Number of Transcripts	128	967	1042	201	1462	1782
Number and % of Annotated Transcripts	72 (56.25%)	558 (57.7%)	669 (64.2%)	93 (46.27%)	641 (43.84%)	763 (42.82%)
Number and % of Novel Transcripts	56 (43.75%)	409 (42.3%)	373 (35.8%)	108 (53.73%)	821 (56.16%)	1019 (57.18%)
FSM	50	424	412	71	492	485
ISM	22	134	257	22	149	278
NIC	43	313	288	61	523	670
NNC	13	96	85	47	298	349
Genic Genomic	0	0	0	0	0	0
Antisense	0	0	0	0	0	0
Fusion	0	0	0	0	0	0
Intergenic	0	0	0	0	0	0
Genic Intron	0	0	0	0	0	0
IR Genes (% of all IR Genes)	9(0.35%)	75(2.92%)	62(2.42%)	9(0.27%)	103(3.05%)	98(2.9%)
IR Genes (% of Detected)	9(27.27%)	75(26.04%)	62(19.56%)	9(21.95%)	103(32.39%)	98(28.65%)
NMD Genes (% of Detected)	8(24.24%)	58(20.14%)	47(14.83%)	9(21.95%)	78(24.53%)	75(21.93%)
IR and NMD genes (% of Detected)	4(12.12%)	29(10.07%)	24(7.57%)	4(9.76%)	44(13.84%)	36(10.53%)
Fusion Genes	1	5	8	1	10	10
Number of Detected Genes with >1 isoform	23(69.7%)	194(67.36%)	221(69.72%)	34(82.93%)	251(78.93%)	281(82.16%)

Table S19: Summary of transcripts mapping to disease-associated genes in human and mouse cortex, related to Figure 4. Isoform diversity was assessed in genes robustly associated with autism (393 genes nominated as being category 1 (high confidence) and category 2 (strong candidate) from the SFARI Gene database https://gene.sfari.org/), Alzheimer's disease (three familial AD genes and 59 genes nominated from the most recent GWAS meta-analysis) and schizophrenia (SZ) (339 genes nominated from the most recent GWAS meta-analysis). AD – Alzheimer's disease, SZ – Schizophrenia. IR – Intron retention, NMD – Nonsense-mediated mRNA decay, FSM – Full Splice Match, ISM – Incomplete Splice Match, NIC – Novel In Catalogue, NNC – Novel Not in Catalogue

Iso-Seq Gene Expression threshold ($Log_{10}TPM$)	Human-Mouse correlation of number of isoforms	P-value	Number of genes surpassing expression threshold
0	0.506	0	20404
0.5	0.502	0	19531
1	0.495	0	15023
1.5	0.509	0	9382
2	0.53	7.50×10^{-296}	4094
**2.5**	**0.543**	$\mathbf{4.91 \times 10^{-87}}$	**1123**
3	0.635	2.51×10^{-25}	212
3.5	0.751	1.33×10^{-07}	36
4	-0.091	NA	4

Table S22: Determining a common high gene expression threshold between human and mouse, related to STAR Methods. A high gene expression threshold was applied to a few analyses to further understand the relationship between isoform number and gene length (**Figure S5**), isoform number and gene exon number (**Figure S5C,S5D,S5G and S5H**), and to investigate whether there was a difference in intron retention rate between highly-expressed and lowly-expressed genes (**Figure S15G**). A gene expression cut-off was sequentially applied to both human and mouse cortex Iso-Seq dataset, and the number of isoforms of the filtered genes were then correlated. Subsequently, the gene expression threshold was determined by the gene expression at which number of isoforms for commonly expressed genes was most correlated between human and mouse with still a significant number surpassing the threshold – in this case, 2.5 $Log_{10}TPM$. Of note, the genes filtered could have an expression surpassing threshold in mouse but not in human, and vice versa. TPM – Transcripts per Million
