# Supplementary material for: Full-length transcript sequencing of human and mouse cerebral cortex identifies widespread isoform diversity and alternative splicing
Source: Cell Rep. 2021 Nov 16;37(7):110022. doi: 10.1016/j.celrep.2021.110022 (PMC8609283; doi:10.1016/j.celrep.2021.110022)
Supplement: Document S2. Article plus supplemental information [file mmc16.pdf]

Cell Reports

Resource

Full-length transcript sequencing of human and mouse cerebral cortex identifies widespread isoform diversity and alternative splicing

Graphical abstract

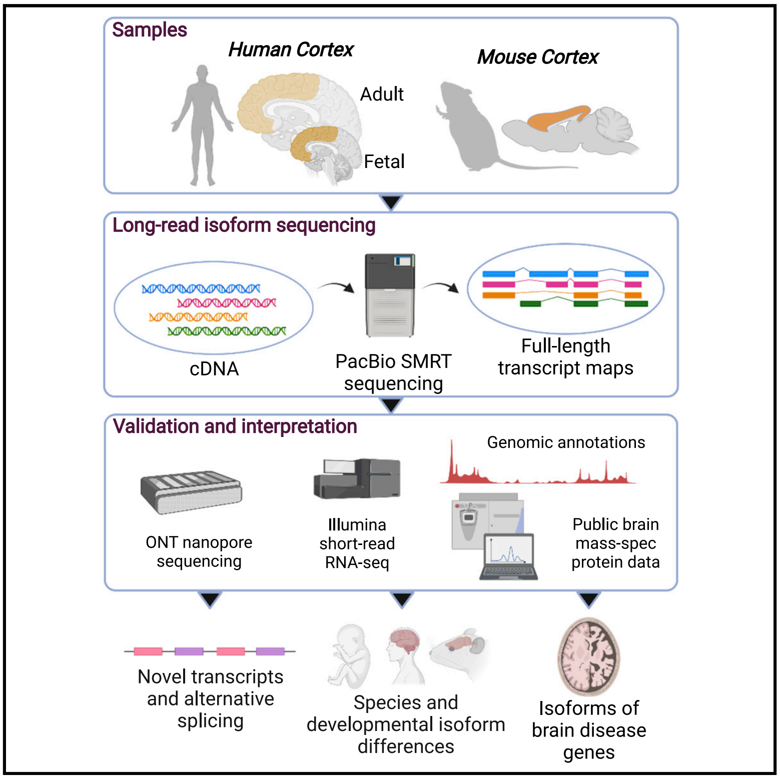

Highlights

- There is widespread transcript diversity in the cortex and many novel transcripts
- Some genes display big differences in isoform number between human and mouse cortex
- There is evidence of differential transcript usage between human fetal and adult cortex
- There are many novel isoforms of genes associated with human brain disease

Authors

Szi Kay Leung, Aaron R. Jeffries, Isabel Castanho, ..., Gloria M. Sheynkman, Eilis Hannon, Jonathan Mill

Correspondence

J.mill@exeter.ac.uk

In brief

Leung et al. use long-read sequencing to annotate RNA isoforms in the human and mouse cortex. They identify novel transcripts and evidence for differential transcript usage between the fetal and adult cortex. Their data confirm the importance of alternative splicing as a mechanism underpinning gene regulation in the brain.

Leung et al., 2021, Cell Reports *37*, 110022
November 16, 2021 © 2021 The Author(s).
https://doi.org/10.1016/j.celrep.2021.110022

CellPress

Cell Reports

CellPress
OPEN ACCESS

Resource

Full-length transcript sequencing of human and mouse cerebral cortex identifies widespread isoform diversity and alternative splicing

Szi Kay Leung,[1,13] Aaron R. Jeffries,[1,13] Isabel Castanho,[1,2,3] Ben T. Jordan,[4] Karen Moore,[1] Jonathan P. Davies,[1] Emma L. Dempster,[1] Nicholas J. Bray,[5] Paul O'Neill,[1] Elizabeth Tseng,[6] Zeshan Ahmed,[7] David A. Collier,[7] Erin D. Jeffery,[4] Shyam Prabhakar,[8] Leonard Schalkwyk,[9] Connor Jops,[10] Michael J. Gandal,[10] Gloria M. Sheynkman,[4,11,12] Eilis Hannon,[1] and Jonathan Mill[1,14,*]

[1]University of Exeter, Exeter, UK
[2]Department of Pathology, Beth Israel Deaconess Medical Center, Boston, MA, USA
[3]Harvard Medical School, Boston, MA, USA
[4]Department of Molecular Physiology and Biological Physics, University of Virginia, Charlottesville, VA, USA
[5]School of Medicine, Cardiff University, Cardiff, UK
[6]Pacific Biosciences, Menlo Park, CA, USA
[7]Eli Lilly & Co., Windlesham, UK
[8]Genome Institute of Singapore, Agency for Science, Technology and Research (A*STAR), Singapore, Singapore
[9]School of Life Sciences, University of Essex, Colchester, UK
[10]Department of Psychiatry and Biobehavioral Sciences, Semel Institute for Neuroscience and Human Behavior, University of California Los Angeles, Los Angeles, CA, USA
[11]Department of Human Genetics, David Geffen School of Medicine, University of California Los Angeles, Los Angeles, CA, USA
[12]UVA Cancer Center, University of Virginia, Charlottesville, VA, USA
[13]These authors contributed equally
[14]Lead contact
*Correspondence: J.mill@exeter.ac.uk
https://doi.org/10.1016/j.celrep.2021.110022

SUMMARY

Alternative splicing is a post-transcriptional regulatory mechanism producing distinct mRNA molecules from a single pre-mRNA with a prominent role in the development and function of the central nervous system. We used long-read isoform sequencing to generate full-length transcript sequences in the human and mouse cortex. We identify novel transcripts not present in existing genome annotations, including transcripts mapping to putative novel (unannotated) genes and fusion transcripts incorporating exons from multiple genes. Global patterns of transcript diversity are similar between human and mouse cortex, although certain genes are characterized by striking differences between species. We also identify developmental changes in alternative splicing, with differential transcript usage between human fetal and adult cortex. Our data confirm the importance of alternative splicing in the cortex, dramatically increasing transcriptional diversity and representing an important mechanism underpinning gene regulation in the brain. We provide transcript-level data for human and mouse cortex as a resource to the scientific community.

INTRODUCTION

Alternative splicing (AS) is a post-transcriptional regulatory mechanism producing multiple RNA isoforms from a single mRNA precursor. In eukaryotes, AS dramatically increases transcriptomic and proteomic diversity from the coding genome and is an important mechanism in the developmental control of gene expression. The mechanisms involved in AS include the use of alternative first (AF) and last (AL) exons, exon skipping (SE), alternative 5′ (A5′) and A3′ splice sites, mutually exclusive exons (MX), and intron retention (IR) (Wang et al., 2008). These phenomena are common, influencing the transcription of >95% of human genes (Pan et al., 2009). Because alternatively spliced transcripts from a single gene can produce proteins with different functions (Eksi et al., 2013; Yang et al., 2016), there is increasing interest in their role in human disease (Wang and Cooper, 2007). Of note, the correction of AS deficits has been shown to have therapeutic benefit in several disorders including spinal muscular atrophy (Wan and Dreyfuss, 2017). AS appears to be particularly important and prevalent in the central nervous system (CNS) (GTEx Consortium, 2015), where it impacts neurodevelopment (Mazin et al., 2013), aging (Tollervey et al., 2011), and key neural functions (Raj and Blencowe, 2015). AS is a common feature of many neuropsychiatric and neurodegenerative diseases (Mills and Janitz, 2012), with recent studies highlighting splicing differences associated with autism (Parikshak et al., 2016),

This is an open access article under the CC BY license (http://creativecommons.org/licenses/by/4.0/).

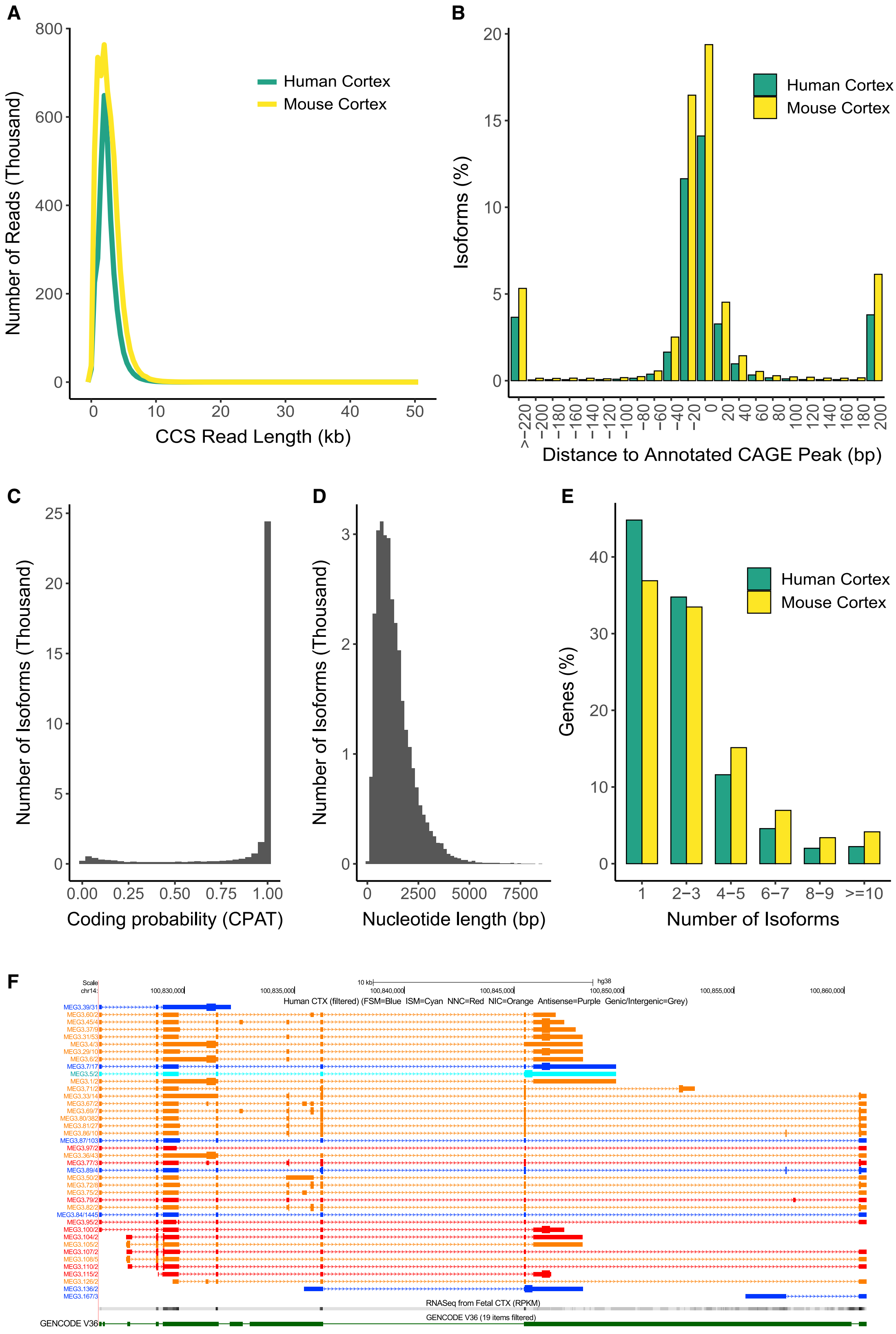

(legend on next page)

schizophrenia (SZ) (Takata, Matsumoto and Kato, 2017), and Alzheimer's disease (AD) (Raj et al., 2018).

Characterizing the full complement of isoforms across tissues and development is important for understanding transcriptional variation in health and disease. For example, transcript-level annotation can be used to improve the understanding of the functional consequences of rare genetic variants (Cummings et al., 2020). However, efforts to fully characterize RNA isoform diversity are constrained by standard RNA sequencing (RNA-seq) approaches, which generate short reads that cannot span full-length transcripts (Steijger et al., 2013). Recent advances in long-read sequencing have addressed these challenges; Pacific Biosciences (PacBio) single-molecule real-time (SMRT) sequencing and Oxford Nanopore Technologies (ONT) nanopore sequencing can generate reads >10 Kb, enabling direct assessment of alternatively spliced transcripts (Amarasinghe et al., 2020).

In this study, we systematically characterize RNA isoform diversity in the cerebral cortex, a key region of the brain involved in perception, cognition, and consciousness. We first use the PacBio isoform sequencing (Iso-Seq) approach (Gordon et al., 2015) to generate full-length cDNA sequences from the human and mouse cortex. We identify widespread transcript diversity with the detection of novel transcripts not previously described in existing genomic annotations, including in genes associated with neuropsychiatric and neurodegenerative disease. We subsequently use ONT nanopore sequencing and short-read RNA-seq to validate and complement our Iso-Seq data. We find widespread evidence of different AS events and examples of fusion genes representing read-through transcription between adjacent genes. A comparison of human and mouse cortex identified species-specific transcript diversity, and a comparison of fetal and adult human cortex highlighted developmental changes in AS and transcript expression. Our data confirm the importance of AS in the cortex, dramatically increasing annotated transcriptional diversity and representing an important mechanism underpinning gene regulation in the brain. Our transcript annotations and sequencing data are available as a resource to the research community via browsable tracks and a searchable transcript visualization database.

RESULTS

Methodological overview

An overview of the methods and datasets used in our study is given in Figure S1. PacBio Iso-Seq data were generated on RNA isolated from human cortex tissue (n = 7) dissected from fetal (n = 3, mean age = 16 weeks post-conception [WPC], range = 14–17 WPC) and adult (n = 4, mean age = 61.8 years, range = 24–89 years) donors (Table S1). Raw reads were processed using the *Iso-Seq* pipeline (Gordon et al., 2015), mapped to the genome, and clustered using *cDNA Cupcake*, followed by *SQANTI2* (Tardaguila et al., 2018) annotation (Table S2). In parallel, we generated a mouse cortex Iso-Seq dataset (n = 12, mean age = 5 months, range = 2–8 months; Table S1) and also profiled tissue from two additional human brain regions (hippocampus and striatum). Rarefaction curves confirmed that our coverage of RNA isoform diversity is representative of the population of transcripts present (Figures S2A–S2F). All downstream analyses and statistics reported in our manuscript are based on the subset of *SQANTI2*-filtered transcripts unless otherwise indicated, although the extended (unfiltered) datasets are available as genome browser tracks as a resource. To validate the transcripts identified using Iso-Seq, we generated short-read RNA-seq data (human: n = 3; mouse: n = 12) and additional full-length transcriptome data using nanopore sequencing (ONT) in a subset of samples (human: n = 2; Table S1). Taken together, our analysis represents the most comprehensive characterization yet undertaken of full-length transcripts and transcript diversity in the human and mouse cortex.

Iso-Seq identifies widespread transcript diversity in the human cortex

We obtained a total of 3.30 M (million) circular consensus sequence (CCS) reads from the human cortex samples (Table S3), with the majority of reads 2 to 3 kb in length (mean length = 2.46 kb; Figure 1A; Figures S3A–S3C), corresponding to the mean length of mRNA in the human genome (Piovesan et al., 2019). Following stringent quality control (QC), these reads mapped to 12,910 "annotated" genes (Table 1) with expression patterns reflecting those expected for the cortex; using the Human Gene Atlas database (Kuleshov et al., 2016), the 500 most abundantly expressed genes were most enriched for "prefrontal cortex" (odds ratio = 5.99, adjusted $p = 9.18 \times 10^{-24}$) (Table S4). In total, we identified 32,802 unique transcripts (mean length = 2.77 kb, SD = 1.29 kb, range = 0.104–11.8 kb) in the human cortex (Table 1); as expected, these were enriched near Cap Analysis Gene Expression (CAGE) peaks from the FANTOM5 dataset (Lizio et al., 2019) (median distance from a CAGE peak = −1 bp with 25,762 [78.5%] of transcripts located within 50 bp of a CAGE peak) (Figure 1B) and were also located proximally to annotated transcription start sites and transcription termination sites (Figures S4B and S4C). Using the Coding-Potential Assessment Tool (CPAT) (Wang et al., 2013) to characterize open reading frames (ORFs) among detected transcripts, we identified a high level of coding potential: 29,998 (91.5%) of the

Figure 1. Generation of high-quality long-read transcriptome datasets for human and mouse cerebral cortex

(A) The distribution of CCS read lengths in our human (n = 7 biologically independent samples) and mouse (n = 12 biologically independent samples) cortex Iso-Seq datasets. The distribution of CCS read lengths for individual samples can be found in Figure S3.

(B) Distance between transcription start site (TSS) and closest annotated CAGE peak. A negative value refers to a CAGE peak located upstream of a TSS.

(C) The distribution of coding potential scores for all transcripts detected in the human cortex.

(D) The ORF lengths for transcripts predicted to be protein-coding. Equivalent plots for mouse cortex can be found in Figures S7A and S7B.

(E) The number of isoforms identified per gene detected in the human and mouse cortex.

(F) UCSC genome browser track of transcripts annotated to *MEG3* in the human cortex. Transcripts are colored based on *SQANTI2* classification categories (blue = FSM; cyan = ISM; red = NIC; orange = NNC).

Table 1. An overview of the whole-transcriptome Iso-Seq datasets generated on human and mouse cerebral cortex

	Human cortex	Mouse cortex	Adult cortex	Fetal cortex
Unique genes	12964	14684	11021	9679
Annotated genes (%)	12910 (99.58)	14482 (98.62)	10987 (99.69)	9660 (99.8)
Novel genes (%)	54 (0.42)	202 (1.38)	34 (0.31)	19 (0.2)
Isoforms	32802	46626	22048	18612
Genes with >1 isoform (%)	7155 (55.19)	9266 (63.1)	5003 (45.4)	4200 (43.39)
Genes with >10 isoforms (%)	205 (1.58)	466 (3.17)	66 (0.6)	50 (0.52)
Protein-coding transcripts (%)	30411 (92.71)	43530 (93.36)	20537 (93.15)	17464 (93.83)
Non-protein-coding transcripts (%)	2391 (7.29)	3096 (6.64)	1511 (6.85)	1148 (6.17)
Known transcripts (FSM, ISM) (%)	20832 (63.51)	23530 (50.47)	15659 (71.02)	13177 (70.8)
Novel transcripts (%)	11970 (36.49)	23096 (49.53)	6389 (28.98)	5435 (29.2)
FSM (%)	17080 (52.07)	19803 (42.47)	13007 (58.99)	11346 (60.96)
ISM (%)	3752 (11.44)	3727 (7.99)	2652 (12.03)	1831 (9.84)
NIC (%)	8721 (26.59)	13763 (29.52)	4464 (20.25)	4315 (23.18)
NNC (%)	3021 (9.21)	8751 (18.77)	1796 (8.15)	1041 (5.59)
Genic genomic (%)	35 (0.11)	62 (0.13)	20 (0.09)	8 (0.04)
Antisense (%)	31 (0.09)	119 (0.26)	22 (0.1)	7 (0.04)
Fusion (%)	136 (0.41)	297 (0.64)	74 (0.34)	51 (0.27)
Intergenic (%)	26 (0.08)	104 (0.22)	13 (0.06)	13 (0.07)
Genic intron (%)	0 (0)	0 (0)	0 (0)	0 (0)

FSM = full splice match; ISM = incomplete splice match; NIC = novel in catalogue; NNC = novel not in catalogue.

detected transcripts were predicted to be protein-coding (Figure 1C) with an average ORF length of 1,327 nucleotides (Figure 1D). A wide range in the number of multi-exonic RNA isoforms was identified per gene (n = 1–40; Table S5), with over half of all detected genes (n = 7,155 [55.2%]) characterized by more than one isoform, and a notable proportion characterized by more than ten isoforms (n = 205 [1.58%]) (Figure 1E). *MEG3,* a maternally expressed imprinted long non-coding RNA (lncRNA) gene involved in synaptic plasticity (Tan et al., 2017), displayed the greatest isoform diversity in human cortex (40 isoforms; Figure 1F). Gene Ontology (GO) analysis showed that the most enriched molecular function among the 100 most isoformic genes in human cortex was "pre-mRNA binding" (human cortex: odds ratio = 31.8, adjusted $p = 2.39 \times 10^{-3}$) (Table S4), an interesting observation given the role that RNA-binding proteins (RBPs) themselves play in regulating tissue-specific patterns of AS (Fu and Ares, 2014). The number of detected isoforms was correlated with both gene length (corr = 0.19, $p = 1.52 \times 10^{-106}$; Figure S5A) and the number of exons (corr = 0.24, $p = 7.97 \times 10^{-155}$; Figure S5E), with these relationships being stronger among "highly expressed" (> 2.5 Log_{10} transcripts per million [TPM]) genes (correlation between isoform number and gene length = 0.49; $p = 1.39 \times 10^{-33}$; correlation between isoform number and number of exons = 0.45, $p = 7.42 \times 10^{-28}$; Figures S5C and S5G), reflecting the additional sensitivity for detecting transcripts of highly expressed genes.

Novel transcripts were detected for a large proportion of expressed genes in the human cortex

Among full-length transcripts annotated to known genes (n = 32,745 transcripts) in the human cortex, the majority were characterized either as a complete full splice match (FSM: n = 17,080 [52.2%]) or incomplete splice match (ISM: n = 3,752 [11.4%]) to existing annotations in GENCODE (hg38) (Figure 2B; Table S6). A significant proportion of transcripts, however, represented "novel" transcripts not present in existing annotation databases (Table S7): 11,913 transcripts (36.4%) associated with 5,327 (41.5%) genes were classified as "novel" (mean size = 2.84 kb, SD = 1.2 kb, range = 0.104–11.2 kb, mean number of exons = 11.1) (Figure 2B; Figure S6A). Most of these novel transcripts contained a combination of known donor and acceptor splice sites and were classified as "novel in catalog" (NIC: n = 8,721, 73.2% of all novel transcripts of known genes). The remaining novel transcripts were predominantly classified as "novel not in catalog" (NNC), with at least one novel donor or acceptor site (n = 3,021, 25.4% of all novel transcripts of known genes). Novel transcripts were generally less abundant than annotated transcripts (Mann-Whitney-Wilcoxon test: $W = 1.62 \times 10^{8}$, $p < 2.23 \times 10^{-308}$; Figures S6C and S6D) and presumably harder to detect using standard RNA-seq approaches (Conesa et al., 2016). Novel transcripts were also longer ($W = 1.10 \times 10^{8}$, $p = 4.04 \times 10^{-25}$) and had more exons ($W = 8.84 \times 10^{7}$, $p < 2.23 \times 10^{-308}$) (Figures S6E and S6F). Finally, the majority of novel transcripts (n = 9,538 transcripts, 80% of novel transcripts) were within 50 bp of an annotated CAGE peak from the FANTOM5 database (Figure S4B).

NIC, NNC, and ISM transcripts were characterized by a similar distribution of predicted ORF lengths and CPAT coding probability scores to FSM transcripts, although the protein coding potential of NIC and NNC transcripts was marginally lower (Figures 2C and 2D). We used public mass spectrometry (MS)-based human cortex proteomics data to look for evidence of translation of NIC

CellPress
OPEN ACCESS

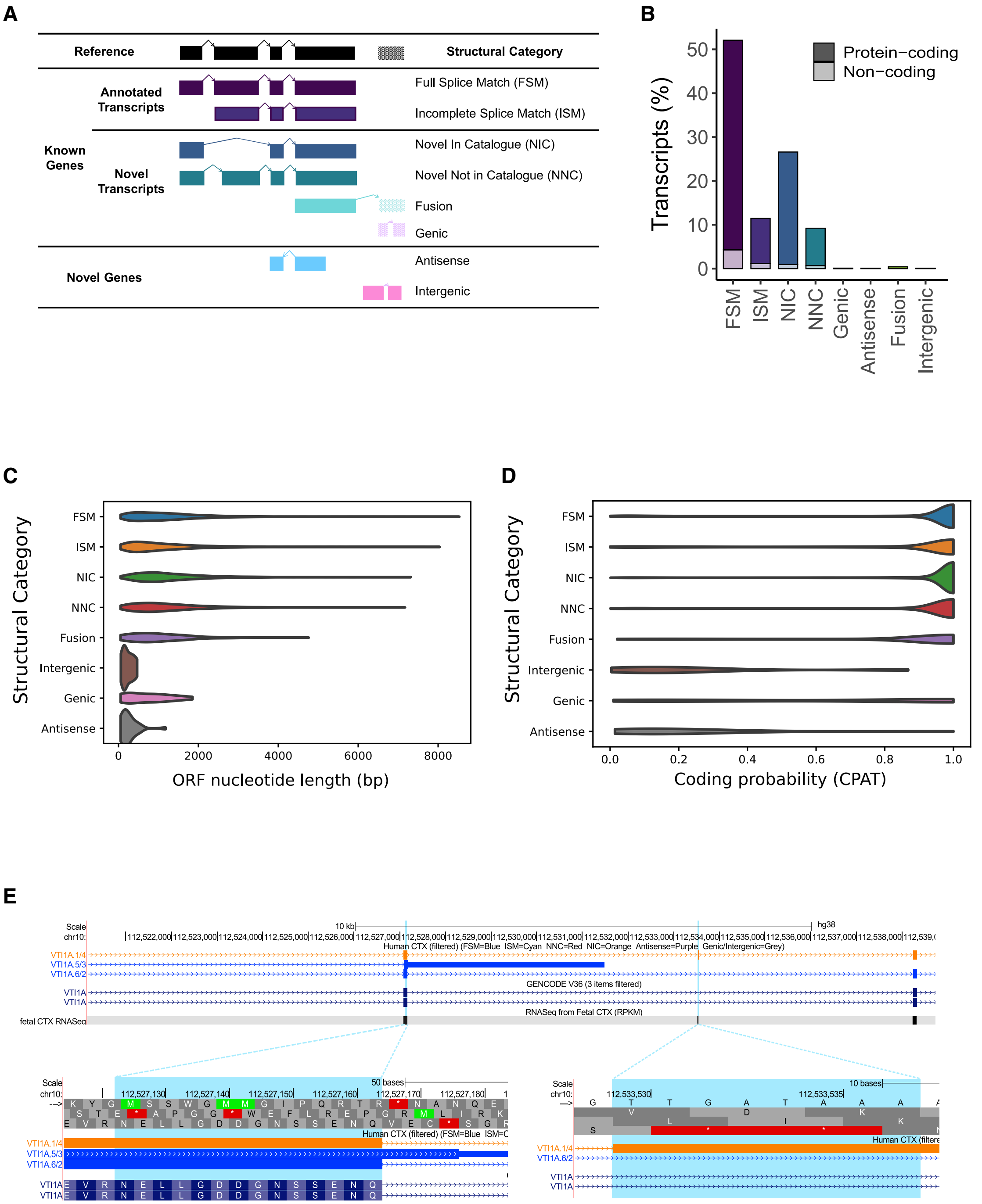

Figure 2. A large proportion of cortical transcripts are not described in existing annotations

(A) A transcript was classified as "FSM" if it aligned with the reference genome with the same splice junctions and contained the same number of exons; "ISM" if it contained fewer 5′ exons than the reference genome; "NIC" if it represented a novel transcript containing a combination of known donor or acceptor sites; and "NNC" if it represented a novel transcript with at least one novel donor or acceptor site.

(B) Approximately half of all transcripts identified in the human cortex were FSM, with a large proportion of transcripts assigned as being novel (NIC, NNC).

(C and D) Distribution of (C) ORF length and (D) coding probability of transcripts by category. A similar ORF length and CPAT probability score profile was observed for FSM, NIC, and NNC transcripts. Equivalent plots for mouse cortex can be found in Figures S7C and S7D.

(E) Shown is a UCSC genome browser track of *VTI1A* in the human cortex. Interrogation of human protein data identified a peptide (NELLGDDGNSSENQLIK, highlighted blue) that confirmed inclusion of a novel exon.

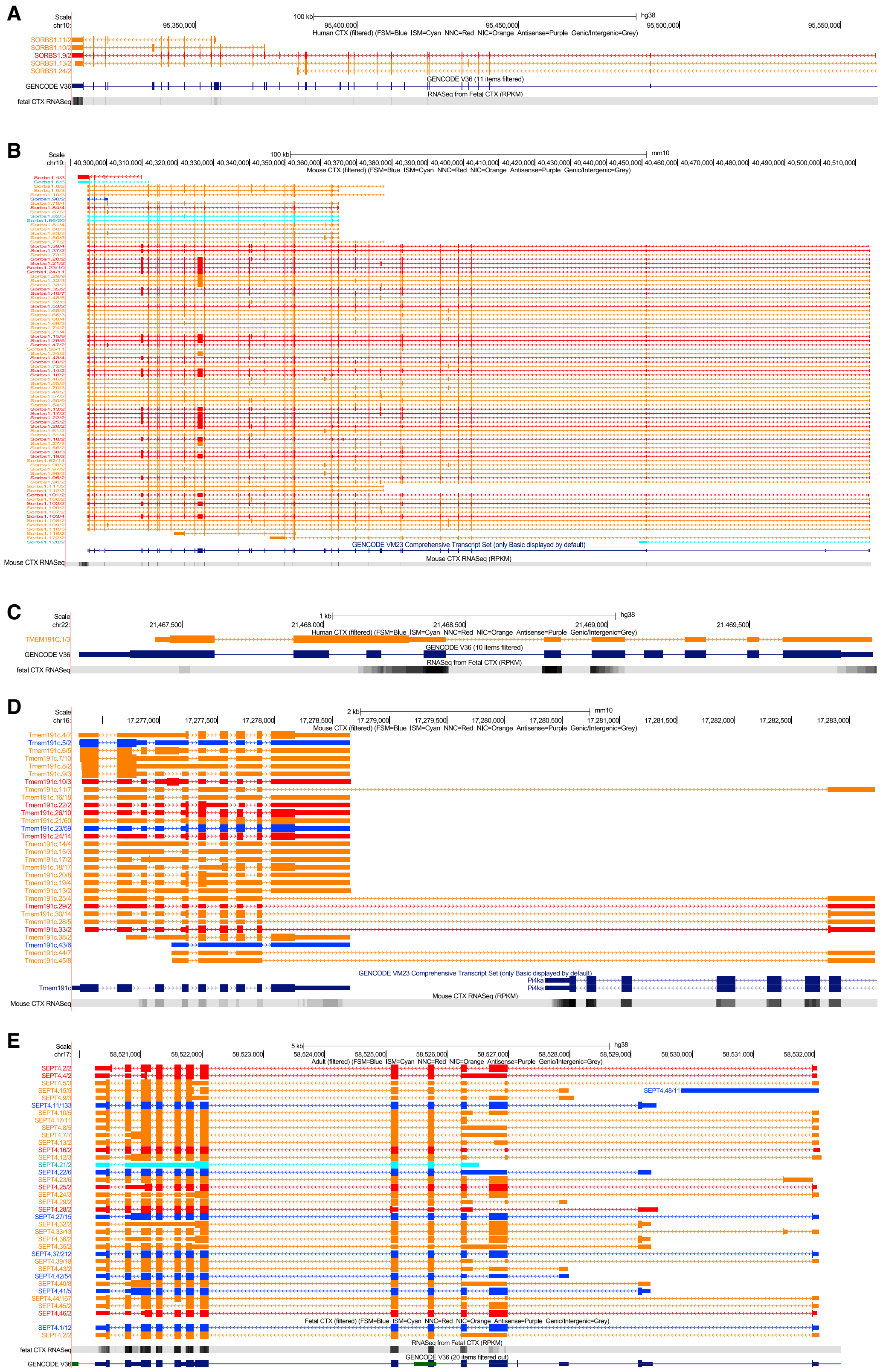

(legend on next page)

and NNC transcripts. Briefly, using the ORFs predicted from CPAT, we assembled a cortex-specific full-length protein database and searched the results against a bottom-up proteomics dataset generated from adult and fetal human brain cortex samples. We found examples of novel peptides, each mapping uniquely to one or more novel transcript(s), providing evidence for the stable translation of these isoforms in the cortex (Table S8); Figure 2E shows a peptide assigned to a novel transcript of *VTI1A*—a gene encoding a soluble N-ethylmaleimide-sensitive factor attachment protein receptor with neuron-specific functions (Tang, 2020)—providing evidence for translation of a protein isoform with a novel exon.

Overall patterns of transcript diversity are similar between human and mouse cortex

We generated a parallel Iso-Seq dataset on mouse cortex, obtaining 5.66 M CCS reads with similar size profiles (mean length = 2.57 kb; Figure 1A; Figure S3B) to those seen in human cortex. These reads mapped to 14,482 annotated genes (Table 1), with the 500 most abundantly expressed genes being primarily enriched for "cerebral cortex" genes in the Mouse Gene Atlas database (Kuleshov et al., 2016) (odds ratio = 6.07, adjusted $p = 6.8 \times 10^{-17}$; Table S4). We identified 46,626 unique transcripts (mean length = 3.18 kb, SD = 1.68 kb, range = 0.083–15.9 kb) in the mouse cortex (Table 1), which were again enriched near CAGE peaks (median distance from CAGE peak = −1 bp, 35,262 [75.6%] transcripts located within 50 bp of a CAGE peak; Figure 1B). A wide range in the number of multi-exonic RNA isoforms was also identified per gene (1 to 86) (Table S5), with a similar distribution to that observed in the human cortex (n = 9,266 genes [63.1%] with more than one isoform, n = 466 [3.17%] with more than ten isoforms) (Figure 1E). The number of detected RNA isoforms was also correlated with both gene length (corr = 0.25, $p = 1.33 \times 10^{-197}$; Figure S5B) and exon number (corr = 0.25, $p = 4.02 \times 10^{-193}$; Figure S5F), with a stronger relationship observed among "highly expressed" genes (Figures S5D and S5H). As in the human cortex, we identified a large proportion of novel transcripts associated with known genes (n = 22,873 [49.3%], mean size = 3.28 kb, SD = 1.61 kb, range = 0.182–15.0 kb, mean number of exons = 12.4), with the vast majority identified as either NIC (n = 13,763 [60.2%]) or NNC (n = 8,751 [38.3%]) (Figure S6A). They were also less abundant ($W = 3.66 \times 10^8$, $p < 2.23 \times 10^{-308}$), longer ($W = 2.37 \times 10^8$, $p = 2.13 \times 10^{-42}$), and had more exons ($W = 1.94 \times 10^8$, $p < 2.23 \times 10^{-308}$) than already known transcripts (Figures S6C–S6H), with the majority (n = 17,252 [75.4%]) mapping to within 50 bp of an annotated CAGE peak (Figure S4A). Finally, predicted coding potential across different transcript categories reflected those observed in human cortex (Figures S7A–S7D).

A subset of genes is characterized by major differences in transcript diversity between human and mouse cortex

Although previous studies have highlighted evidence of major splicing diversity between human and mouse (Ule and Blencowe, 2019), we found that among multi-exonic genes, for which transcripts were detected in both human and mouse cortex (n = 10,202 genes; Figure S8A), the number of isoforms detected for each gene was significantly correlated between species (corr = 0.51, $p < 2.23 \times 10^{-308}$; Figure S8C). There was a stronger relationship among highly expressed genes (> 2.5 Log_{10} TPM in both species, corr = 0.64, $p = 1.21 \times 10^{-25}$; Figure S8E), a possible reflection of a deeper sequencing coverage of these genes. Despite the overall stability in cortical RNA isoform diversity between human and mouse, there were striking exceptions for specific genes (Table S5). *SORBS1*(Figures 3A and 3B) and *ARPP21* (Figures S9A and S9B) had the largest absolute difference in numbers of isoforms detected between human and mouse. *LPAR2* had the highest *relative* number of isoforms detected in human cortex (n = 12 isoforms; Figure S9C) compared to mouse cortex (1 isoform; Figure S9D) Figures S9, whereas *Tmem191c* had the highest *relative* number of isoforms in mouse cortex (n = 30 isoforms; Figure 3C) compared to human cortex (1 isoform; Figure 3D).

Comparisons with short-read RNA-seq data and nanopore sequencing confirms the accuracy and sensitivity of Iso-Seq

Although Iso-Seq is accurate at characterizing RNA diversity (Wang et al., 2019), its sensitivity for quantifying gene expression has not been systematically explored. We generated highly parallel RNA-seq data on a subset of samples (Table S9), finding a strong correlation between gene-level expression quantified using the two methods in both datasets (human fetal cortex: n = 9,221 genes, corr = 0.54, $p < 2.23 \times 10^{-308}$; mouse cortex: n = 13,923 genes, corr = 0.71, $p < 2.23 \times 10^{-308}$; Figures S10A and S10C). To further assess the quantitative accuracy of Iso-Seq, we included External RNA Controls Consortium (ERCC) spike-in control molecules into our mouse cDNA libraries. Among the detected ERCC transcripts, we found a near-perfect correlation between full-length Iso-Seq reads and the actual amount of control used (corr = 0.98, $p = 1.42 \times 10^{-41}$; Figure S10F), highlighting the power of Iso-Seq to accurately quantify the abundance of highly expressed transcripts. The vast majority of unique splice junctions identified in our Iso-Seq data were supported by RNA-seq in both human (n = 89,975 [99.4%] junctions) and mouse (n = 152,872 [98.1%] junctions). For transcripts that could be recapitulated in the matched RNA-seq data, there was a significant correlation between transcript expression levels quantified using both sequencing

Figure 3. A subset of genes are characterized by dramatic differences in cortical transcript diversity between species (human and mouse) and between developmental stages (fetal and adult)

(A–E) UCSC genome browser tracks showing transcripts detected for (A) *SORBS1* in human cortex (n = 5 transcripts); (B) *Sorbs1* in mouse cortex (n = 86 transcripts); (C) *TMEM191C* in human cortex (n = 1 transcript); (D) *Tmem191c* in mouse cortex (n = 30 transcripts); and (E) *SEPT4* in human adult cortex (n = 34 transcripts) and human fetal cortex (n = 2 transcripts).

Additional examples of genes with considerable differences in the number of transcripts between human and mouse cortex are shown in Figures S9A–S9D. Additional examples of genes with considerable differences in the number of transcripts between fetal and adult cortex are shown in Figures S16A and S16B. For each gene, RNA-seq data tracks from human cortex (n = 3 samples) and mouse cortex (n = 12 samples) are also displayed. Transcripts are colored based on *SQANTI2* classification categories (blue = FSM; cyan = ISM; red = NNC; orange = NIC).

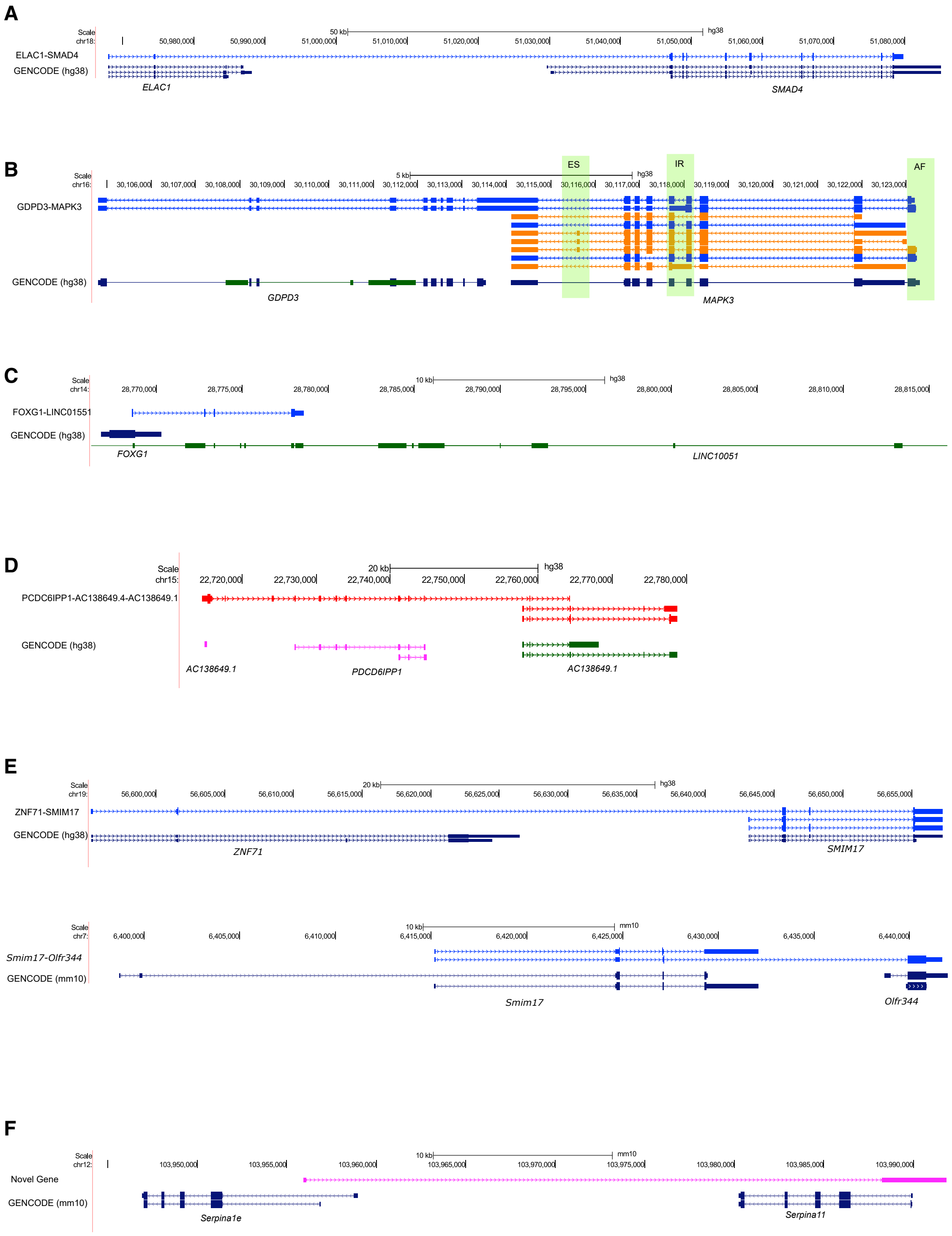

Figure 4. Examples of fusion transcripts in the cortex

(A) A fusion transcript incorporating exons from *ELAC1* and *SMAD4* in the human cortex.

(B) Two read-through transcripts incorporating exons from *MAPK3* and *GDPD3* in the human cortex. Of note, one of the fusion transcripts is characterized by intron retention, as observed in another novel isoform of *MAPK3*.

(C) A fusion transcript incorporating exons from *FOXG1* and *LINC01551* in the human cortex.

(D) A fusion transcript incorporating exons across three pseudogenes in the human cortex.

(legend continued on next page)

approaches (human cortex: n = 17,583 transcripts, corr = 0.40, $p < 2.23 \times 10^{-308}$; mouse cortex: n = 41,488 transcripts, corr = 0.48, $p < 2.23 \times 10^{-308}$; Figures S10B and S10D), further highlighting that transcript abundance can be reliably quantified using Iso-Seq.

Using our Iso-Seq data as a scaffold, we generated a reference-guided transcriptome assembly from our mouse cortex RNA-seq data using *Stringtie* (Pertea et al., 2015). Many of the isoforms reconstructed from RNA-seq reads appeared to represent incomplete fragments of full-length transcripts identified in Iso-Seq. Overall, isoforms assembled using RNA-seq reads had a significantly shorter mean length (RNA-seq: 2.31 kb versus Iso-Seq: 3.18 kb, t = 71.9, $p < 2.2 \times 10^{-16}$), lower average number of exons (RNA-seq: 7.30 versus Iso-Seq: 10.8, t = 76.7, $p < 2.2 \times 10^{-16}$), and were less likely to be located within a CAGE peak (RNA-seq: 34.0% versus Iso-Seq: 71.9%, Fisher's exact test = $p < 2.2 \times 10^{-16}$, odds ratio = 4.97) (Figures S11A and S11B). Importantly, more than 50% of isoforms robustly detected using Iso-Seq could not be readily recapitulated using standard RNA-seq, highlighting the advantage of long-read sequencing for characterizing isoform diversity (Figure S11C). Finally, a large proportion of novel transcripts identified using Iso-Seq (n = 6,417 [53.78%]) were also detected with ONT nanopore sequencing (40.7 M reads) from a subset of samples.

Several cortex-expressed transcripts represent fusion events between neighboring genes

Transcriptional read-through between two or more adjacent genes can produce "fusion transcripts" that represent an important class of mutation in several types of cancer (McCartney et al., 2019). Although fusion events are thought to be rare (Akiva et al., 2006), we found evidence of fusion transcripts in both the human (n = 136 fusion transcripts [0.41% of all transcripts] associated with 108 genes [0.83% of total genes]); mouse cortex (n = 297 fusion transcripts [0.64% of all transcripts] associated with 218 genes [1.48% of total genes]) (Figure 4A–4E). A number of these genes were associated with more than one fusion transcript (human: n = 22 genes [20.3% of fusion genes]; mouse: n = 53 genes [24.3% of fusion genes]), and we identified examples of fusion transcripts encompassing more than two genes, e.g., a fusion transcript incorporating exons from three adjacent pseudogenes in the human cortex *AC138649.4-AC138649.1-PDCD6IPP1* (Figure 4D). The vast majority of the fusion transcripts identified were supported by RNA-seq data generated on both mouse (n = 282 [95%] transcripts) and human fetal (n = 51 [100%] transcripts) cortex. We also confirmed a significant proportion (n = 46 [33.8%] transcripts) of the human cortex fusion events using our ONT nanopore data. Several of the fusion transcripts identified in the human (n = 4 [2.94% of all fusion transcripts]) and mouse cortex (n = 11 [3.7% of all fusion transcripts]) were predicted as potential "conjoined genes" in the *ConjoinG* database (Prakash et al., 2010). Although the majority of fusion events were specific to the human or mouse datasets, we found evidence of potential protein-coding fusion transcripts incorporating exons from *SMIM17* (*Smim17*) in both species (Figure 4E; Figures S12A–S12D).

Identification of novel cortex-expressed genes using long-read sequencing

Although the vast majority of transcripts identified in both the human and mouse cortex were assigned to annotated genes (human: 99.8% of total transcripts; mouse: 99.5% of total transcripts), a small number represent expression from potentially novel genes (human: n = 57 novel transcripts mapping to 54 novel genes; mouse: n = 223 novel transcripts mapping to 202 novel genes) (Figure 4F; Table S10). These novel genes were either intergenic or antisense to existing annotated genes and were all multi-exonic (human: mean length = 2.09 kb, SD = 1.01 kb, range = 0.254–4.9kb, mean number of exons = 2.9; mouse: mean length = 1.75 kb, SD = 1.21 kb, range = 0.098–6.86 kb, mean number of exons = 2.5). Most transcripts from these novel genes were predicted to be non-coding (human: n = 34 [59.7%] transcripts; mouse: n = 143 [64.1%] transcripts), were generally shorter (human: $W = 1.18 \times 10^{6}$, $p = 7.71 \times 10^{-5}$; mouse: $W = 7.79 \times 10^{6}$, $p = 5.22 \times 10^{-45}$), and less abundant than transcripts of annotated genes (human: $W = 5.28 \times 10^{5}$, $p = 1.72 \times 10^{-19}$; mouse: $W = 2.29 \times 10^{6}$, $p = 1.5 \times 10^{-73}$). Although the majority of these novel genes did not show high sequence homology with other genomic regions, BLAST analysis revealed that 18 (31.6%) of the human cortex novel-gene transcripts and 31 (13.9%) of the mouse cortex novel-gene transcripts showed relatively high similarity (greater than 500 bp, more than 90% identity) to other genomic regions (Table S11). Of the 57 novel-gene transcripts identified in the human cortex, 27 (47.4%) demonstrated evidence of transcription in data from the GTEx consortium (CHESS v2.2 annotation) (Pertea et al., 2018). Further evidence of transcription from a large proportion of the human novel-gene transcripts (n = 28 [49.1%]) was provided by our ONT nanopore sequencing dataset. We used the FANTOM5 CAGE dataset to show that around a quarter of the novel-gene transcripts (human: n = 14 [24.6%]; mouse: n = 58 [26.0%]) were located within 50 bp of a CAGE peak (Table S10). There was an enrichment of antisense transcripts among those mapping to novel genes (human cortex: n = 31 transcripts [54.4%] mapping to 28 novel genes; mouse cortex: n = 119 transcripts [53.4%] mapping to 97 novel genes) (Table S10). The majority of these antisense novel genes were found within an annotated gene (human: n = 25 [89.2% of antisense novel genes], mouse: n = 95 [97.9% of antisense novel genes]), with a relatively large proportion of these sharing exonic regions (human: n = 12 [48%], mouse: n = 72 [74.2%]) reflecting sense-antisense (SAS) pairs (Galante et al., 2007). Finally, there were several striking examples of antisense novel genes overlapping two known genes in the mouse cortex (Figure 4F).

(E) Fusion transcripts with exons from *SMIM17*/*Smim17* were identified in both human and mouse cortex. Additional examples of overlapping fusion transcripts between human and mouse cortex are shown in Figures S12A–S12D.

(F) An example of a novel antisense transcript spanning *Serpina1e* and *Serpina11* in the mouse cortex. Transcripts are colored based on *SQANTI2* classification categories (blue = FSM; cyan = ISM; red = NNC; orange = NIC).

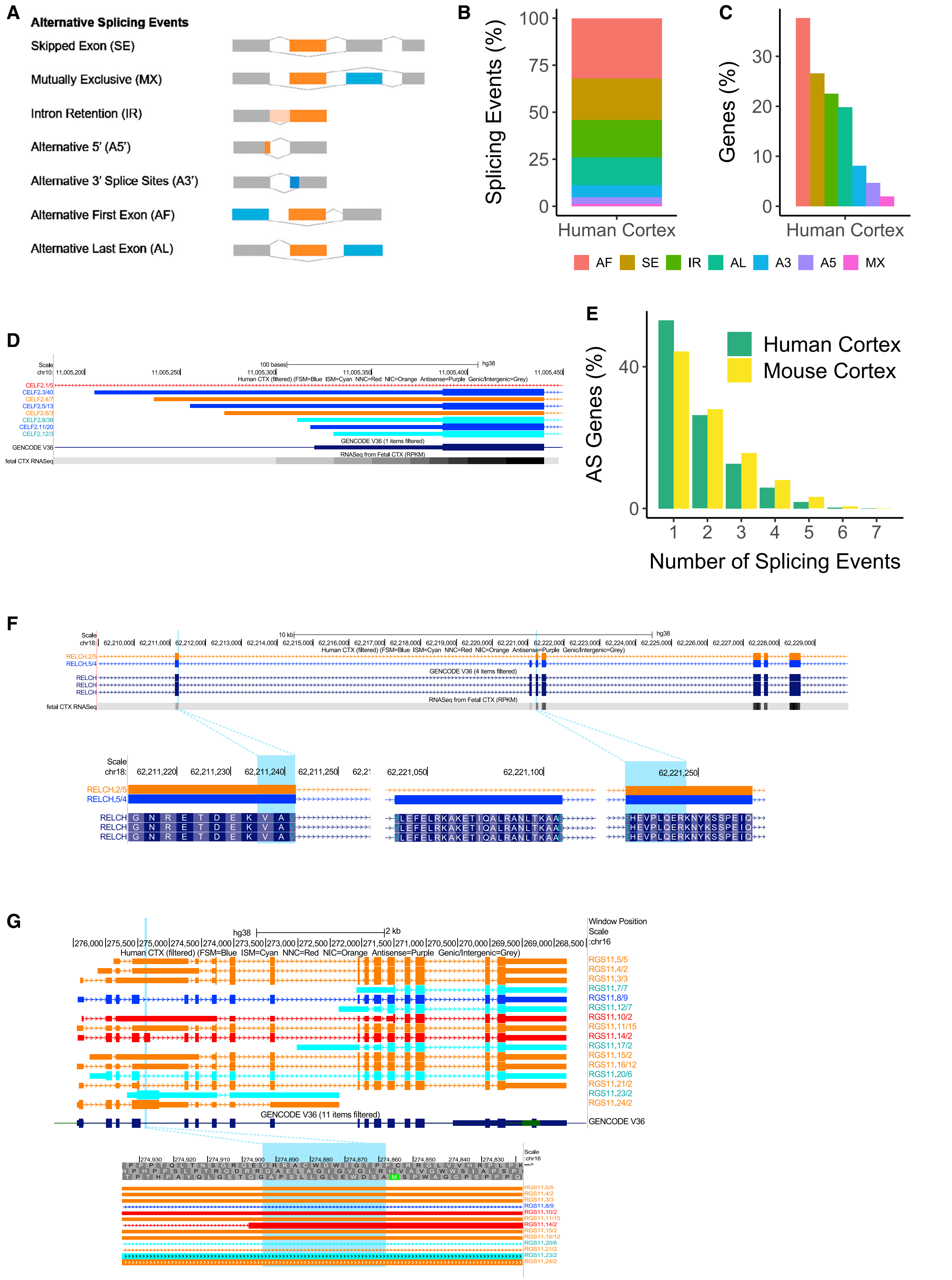

(legend on next page)

Many transcripts map to lncRNA genes with a subset containing predicted ORFs

Although the majority of transcripts were classified as protein-coding by the presence of an ORF, a relatively large number of transcripts were annotated as encoding lncRNA (human: n = 1,197 transcripts associated with 792 genes; mouse: n = 1,141 transcripts associated with 734 genes). These lncRNA transcripts were shorter than non-lncRNA transcripts (human: mean length of lncRNA transcripts = 2.32 kb [SD = 1.14 kb, range = 0.104–7.78 kb], mean length of non-lncRNA transcripts = 2.78 kb [SD = 1.29 kb, range = 0.107–11.8 kb], $W = 2.28 \times 10^{7}$, $p = 3.22 \times 10^{-34}$; mouse: mean length of lncRNA transcripts = 2.22 kb [SD = 1.36 kb, range = 0.148–.49 kb], mean length of non-lncRNA transcripts = 3.21 kb [SD = 1.68 kb, range = 0.083–15.9 kb], $W = 3.52 \times 10^{7}$, $p = 8.24 \times 10^{-98}$). As reported previously they alsocontained fewer exons (Statello et al., 2021) (human: $W = 3.31 \times 10^{7}$, $p < 2.23 \times 10^{-308}$; mouse: $W = 4.56 \times 10^{7}$, $p < 2.23 \times 10^{-308}$), with a dramatic enrichment of monoexonic molecules (Kuo et al., 2017) (human: n = 348 [29.1%]; mouse: n = 273 [23.9%]) compared to non-lncRNA transcripts (human: n = 583 [1.85%]; mouse: n = 914 [2.02%]) (Figures S13A–S13D). They were also characterized by lower transcript expression than non-lncRNA transcripts (Statello et al., 2021; Liu et al., 2016) (human: $W = 2.27 \times 10^{7}$, $p = 9.44 \times 10^{-35}$; mouse: $W = 3.16 \times 10^{7}$, $p = 5.67 \times 10^{-40}$), with fewer isoforms identified per lncRNA gene compared to non-lncRNA genes (human: mean n = 1.51 versus 2.6, $W = 6.63 \times 10^{6}$, $p = 1.21 \times 10^{-80}$; mouse: mean n = 1.55 versus 3.29, $W = 7.40 \times 10^{6}$, $p = 5.76 \times 10^{-107}$) (Figures S13E–S13H). A small proportion of these annotated lncRNA transcripts contained a putative ORF (human: n = 235 [19.6%]; mouse: n = 153 [13.4%]), supporting recent observations that some lncRNA have potential protein coding capacity (Kageyama, Kondo and Hashimoto, 2011), although the majority of such ORFs are unlikely to code for proteins (Guttman et al., 2013); of note, these ORFs were shorter than those identified in non-lncRNA transcripts (human: mean length = 133 bp versus 441 bp, $W = 1.41 \times 10^{7}$, $p = 3.12 \times 10^{-221}$; mouse: mean length = 139 bp versus 519 bp, $W = 1.75 \times 10^{7}$, $p = 8.33 \times 10^{-195}$).

AS events make a major contribution to RNA isoform diversity in the cortex

AS, the process by which different combinations of splice sites within a mRNA precursor are selected to produce variably spliced mRNAs, is the primary mechanism underlying transcript diversity in eukaryotes (Park et al., 2018) and a major source of transcriptional diversity in the CNS (Raj and Blencowe, 2015). Numerous types of AS have been described (Figure 5A), and we used both *SUPPA2* (Trincado et al., 2018) and custom analysis scripts to identify transcripts associated with (1) SE, (2) MX, (3) AF and AL exons, (4) A3′ and A5′ splice sites, and (5) IR in our cortical Iso-Seq data. The overall frequency of these specific AS events was similar in human and mouse cortex, with AF and SE being the most prevalent AS events in both species (human: AF = 8,546 [32.2%] events associated with 4,879 [37.6%] genes, SE: 5,776 [22.0%] events associated with 3,446 [26.6%] genes; mouse: AF = 12,853 [31.9%] events associated with 6,476 [44.1%] genes, SE = 8,686 [21.6%] events associated with 4,570 [31.1%] genes) (Figures 5B and 5C; Figure S14A; Table S12). Using publicly available human brain proteomic data, we found evidence of translated isoforms with novel SE events (Table S8); for example, we identified a novel peptide that was annotated to *RELCH* that spanned across exons 2 and 4 but skipped exon 3 (Figure 5F).

IR is a relatively common form of AS in the cortex that is associated with reduced expression and nonsense-mediated mRNA decay (NMD)

IR, the process by which specific introns remain unspliced in polyadenylated transcripts, is the least understood AS mechanism but is hypothesized to be an important mechanism of transcriptional control in the brain (Jacob and Smith, 2017; Ameur et al., 2011). We found evidence for IR in a relatively large proportion of genes (IR-genes) in both the human (n = 5,231 IR-transcripts associated with 2,566 [19.8%] detected genes) and mouse cortex (n = 6,803 IR transcripts associated with 3,375 [23.0%] genes) (Table S13), with IR-genes themselves enriched for biological processes related to mRNA splicing in human cortex (odds ratio = 3.24, adjusted $p = 3.28 \times 10^{-12}$) and mRNA processing in mouse cortex (odds ratio = 2.97, p = adjusted 7.74×10^{-13}, Table S4). The majority of IR-transcripts were supported by matched short-read RNA-seq data from both human (n = 2,713 [97.5%] IR-transcripts) and mouse cortex samples (n = 6,454 [94.9%] IR-transcripts). Most IR-genes were found to express more than one IR-transcript (human cortex: n = 1,463 [72%] IR-genes; mouse cortex: n = 1,872 [72.4%] IR-genes), with *MEG3* having the largest number of IR-transcripts in human cortex (30 isoforms [75% of *MEG3* isoforms]; Figure 1F) and *Entr1* having the largest number of IR-transcripts in mouse cortex (31 isoforms [91.2% of *Entr1* isoforms]). A small number of genes were found to *only* express transcripts characterized by IR (Table S14) (human: n = 197 [7.68% of genes with IR-transcripts, 1.52% of total detected genes]; mouse: n = 150 [4.44% of genes with IR-transcripts, 1.02% of total detected genes]). Overall, there was considerable overlap in the list of IR-genes detected between human and mouse cortex (Figure S15A), with 1,078 homologous genes showing evidence of IR in both the

Figure 5. Alternative splicing (AS) events make a major contribution to transcript diversity in the cortex

(A) An overview of the different types of AS considered in our analysis.

(B) Alternative first (AF) exon use is the most prevalent AS event in both the human cortex and mouse cortex (Figure S14A).

(C) The majority of human cortex-expressed genes are predominantly characterized by AF and SE.

(D) AF events are supported by RNA-seq data. The differing lengths of first exon of *CELF2* in human cortex correspond to differing RNA-seq coverage.

(E) A large proportion of AS genes in human and mouse cortex are characterized by more than one type of splicing event.

(F) Shown is a UCSC genome browser track of *RELCH* with a novel peptide (VAEHEVPLQER, highlighted blue) spanning across exons 2 and 4 of *RELCH* while skipping exon 3, confirming exon skipping in a novel transcript.

(G) A novel peptide (GAELAGIGVGLR, highlighted blue) confirms translation of a retained intronic region observed in a transcript of *RGS11*.

human (48.4% of IR-genes) and mouse (35.3% of IR-genes). Importantly, a larger proportion of lowly expressed genes showed evidence for IR than highly expressed genes in both human (< 2.5 Log_{10} TPM, n = 2,269 [88.4%] genes; > 2.5 Log_{10} TPM, n = 297 [11.6%] genes) and mouse (< 2.5 Log_{10} TPM, n = 3,039 [90.04%] genes; > 2.5 Log_{10} TPM, n = 336 [9.96%] genes; Figure S15G) cortex, corroborating previous analyses suggesting that IR is associated with reduced transcript abundance (Braunschweig et al., 2014). Although most IR-containing transcripts are associated with reduced protein expression, IR-transcripts can produce a stable protein, especially if the intron is relatively short and does not disrupt the translational frame (Grabski et al., 2021). For example, we found evidence for a novel translated IR event involving the 4th intron in *RGS11* in our analysis of MS-based human brain proteomic data (Figure 5G).

NMD acts to reduce transcriptional errors by degrading transcripts containing premature stop codons (Hug, Longman and Cáceres, 2015) and is one mechanism by which IR can influence gene expression (Pan et al., 2006). Overall, >10% of transcripts mapping to annotated genes were predicted to undergo NMD (NMD-transcripts), characterized by the presence of an ORF and a coding sequence (CDS) end motif before the last junction (human cortex: n = 4,370 [13.4%] transcripts associated with 2,323 [18%] of annotated genes; mouse cortex: n = 6,014 [13.0%] transcripts associated with 2,945 [20.3%] of annotated genes). These NMD-transcripts were found to be less abundant than non-NMD-transcripts (human: mean expression of NMD-transcripts = 15.2 TPM, SD = 63.0 TPM, mean expression of non-NMD-transcripts = 33.1 TPM, SD = 261 TPM, W = 4.40×10^7, p = 3.59×10^{-114}; mouse: mean expression of NMD-transcripts = 11.2 TPM, SD = 85.0 TPM, mean expression of non-NMD-transcripts = 23.1 TPM, SD = 143.1 TPM, W = 8.72×10^7, p = 6.15×10^{-156}).

NMD was found to be particularly enriched among IR-transcripts that were predicted to be protein-coding (human: n = 1,930 [38.7%] IR-transcripts associated with 1,104 [8.55%] genes; mouse: n = 2,341 [36.2%] IR-transcripts associated with 1,380 [9.53%] genes), and transcripts with both IR and predicted NMD were particularly lowly expressed (human: W = 4.77×10^6, p = 3.81×10^{-12}; mouse: W = 7.50×10^6, p = 1.67×10^{-42}). Only a small number of genes were associated with transcripts where IR and NMD were mutually exclusive (human: n = 163 [1.26%] genes; mouse: n = 277 [1.91%] genes; Figures S15C–S14F), providing additional support for the hypothesized relationship between these two transcriptional control mechanisms (Ge and Porse, 2014).

Developmental changes in cortical RNA isoform abundance

Our human cortical Iso-Seq dataset included samples derived from both fetal and adult donors, and as expected, there was considerable overlap in the set of genes detected in each (total overlap = 8,111 [84.0% of fetal annotated genes, 73.8% of adult annotated genes]; Figure S8B). Using the Human Gene Atlas database (Kuleshov et al., 2016), we found that the 500 most abundant genes in the fetal cortex dataset were most significantly enriched for "fetal brain" (odds ratio = 6.98, adjusted p = 6.75×10^{-20}), and those in the adult cortex were most significantly enriched for "prefrontal cortex" genes (odds ratio = 6.75, adjusted p = 1.27×10^{-28}; Table S4). In total, we detected 18,592 transcripts mapping to 9,660 annotated genes in the fetal cortex (mean length = 2.90 kb, SD = 1.30 kb, range = 0.132–11.8 kb) and 22,013 transcripts mapping to 10,987 annotated genes in the adult cortex (mean length = 2.53 kb, SD = 1.18 kb, range = 0.104–10.0 kb) (Table S6). Overall patterns of RNA isoform diversity were similar between fetal and adult cortex with a similar number of genes characterized by more than one isoform (fetal: n = 4,200 [43.5%]; adult: 5,003 [45.5%]). A strong correlation was observed between the number of isoforms detected in fetal and in adult human cortex datasets (corr = 0.53, p < 2.23×10^{-308}), which was stronger among highly expressed genes (> 2.5 Log_{10} TPM in both fetal and adult cortex, corr = 0.72, p = 2.54×10^{-42}; Figures S8D and S8F). Despite these similarities, there were some notable exceptions with certain genes characterized by large differences in isoform number between fetal and adult cortex; *SEPT4* had the highest relative number of isoforms detected in adult cortex compared to fetal cortex (34 versus 2 isoforms) (Figure 3E), whereas *CELF3* had the highest relative number of isoforms in fetal cortex compared to adult cortex (11 versus 1 isoforms) (Table S15). *SEPT4*, *RAP1GAP* (adult cortex: n = 25 isoforms; fetal cortex, n = 3 isoforms), and *RUNX1T1* (adult cortex: n = 5 isoforms; fetal cortex: n = 21 isoforms) had the largest absolute difference in isoform numbers detected between human fetal and adult cortex (Figure S16). A similar proportion of novel transcripts were detected in both fetal (n = 5,415 [29.1%] transcripts associated with 3,027 [31.3%] annotated genes) and adult cortex (n = 6,354 [28.9%] associated with 3,468 [31.6%] annotated genes), with 1,670 genes characterized by novel transcripts in both fetal cortex (55.2% of genes with novel transcripts) and adult (48.1% of genes with novel transcripts) cortex. Characterization of ORFs using CPAT revealed a similar distribution of predicted coding potential across different transcript categories between adult and fetal cortex (Figure S7).

We identified 206 transcripts (associated with 189 genes) that were classified as "fetal-specific" and not detected in the adult cortex, and 185 transcripts (associated with 174 genes) that were classified as "adult-specific." We also identified examples of significant differential transcript usage—a switch of dominant isoform expression—between fetal and adult cortex (Table S16). *RTN4*, which encodes a neurite outgrowth inhibitor specific to the CNS (GrandPré et al., 2000), was characterized by the largest expression difference in dominant transcripts between adult- and fetal-specific isoforms (Figure S17A).

A similar frequency of AS events was observed in the human adult and fetal cortex (adult: 4,963 unique AS genes with 14,793 AS events; fetal: 4,231 unique AS genes associated with 11,955 AS events) (Figure S14B; Table S12), with considerable overlap between both datasets (2,812 annotated genes [56.6% of AS genes in adult cortex, 66.5% of AS genes in fetal cortex]). IR was significantly more prevalent in the fetal cortex (2,783 transcripts associated with 1,589 genes [16.4% of annotated genes]) than adult cortex (2,383 transcripts associated with 1,422 genes [12.9% of annotated genes]; odds ratio = 1.45, p = 1.06×10^{-35}, Fisher's exact test), corroborating previous studies suggesting that IR plays a role in the developmental regulation of

gene transcription in the brain (Nellore et al., 2016). Furthermore, although genes with IR-transcripts were generally more lowly expressed, they were more highly expressed in the fetal than the adult cortex ($W = 1.01 \times 10^6$, $p = 7.71 \times 10^{-7}$).

Differential transcript usage across human fetal brain regions

We next generated Iso-Seq data on two additional fetal brain regions (hippocampus and striatum) from matched donors (Table S1). Although the sequencing depth for these additional brain regions was lower than that of the fetal cortex (Table S17), we were able to explore fetal transcriptional differences across fetal hippocampus, striatum, and cortex using a merged dataset (incorporating 24,989 transcripts annotated to 11,072 genes). As expected, there was considerable overlap in genes detected across the three fetal brain regions (2,312 transcripts associated with 2,096 genes with TPM > 20), although a notable subset of transcripts was uniquely expressed in each brain region (cortex: n = 122; hippocampus: n = 25; striatum: n = 58 with TPM > 20). We further identified robust evidence for differential transcript usage across brain regions for a subset of genes (cortex and hippocampus: n = 9 genes; cortex and striatum: n = 10 genes; striatum and hippocampus n = 18 genes) (Table S18). For example, *APLP1* was found to express different isoforms in the cortex and hippocampus; a ~2.0 kb transcript consisting of 16 exons (ENST00000586861.5) was detected in the hippocampus, whereas a ~2.3 kb novel transcript also consisting of 16 exons was detected in the cortex (Figure S17B).

Widespread isoform diversity in genes associated with brain disease

AS has been increasingly implicated in health and disease and is recognized to play a prominent role in brain disorders hypothesized to involve the cerebral cortex including autism, SZ, and AD. There has been considerable progress in identifying genes associated with these disorders using genome sequencing and genome-wide association study (GWAS) approaches (Tam et al., 2019). However, the full repertoire of RNA isoforms transcribed from these genes in the cortex has not been systematically characterized. First, we used the human GWAS catalog database (Kuleshov et al., 2016) to interrogate the most transcriptionally diverse genes in the human cerebral cortex, finding them to be enriched for genes implicated in relevant GWAS datasets (“AD (late onset)”: odds ratio = 10.06, p = 0.004; “autism spectrum disorder or SZ”: odds ratio = 1.94, p = 0.083: “SZ”: odds ratio = 2.70, p = 0.005; Table S4). Second, we assessed RNA isoform diversity in genes robustly associated with AD (three familial AD genes [Bekris et al., 2010] and 59 genes nominated from a recent GWAS meta-analysis [Andrews et al., 2020; Sims et al., 2020]), autism (393 genes nominated as being category 1 [high confidence] and category 2 [strong candidate] from the SFARI Gene database, https://gene.sfari.org/), and SZ (339 genes nominated from the a recent GWAS meta-analysis [Pardiñas et al., 2018]). Among disease-associated genes detected in the cortex, we found evidence for considerable isoform diversity (human cortex: 2,016 transcripts were mapped to 610 disease-associated genes; mouse cortex: 3,218 transcripts were mapped to 670 disease-associated genes; Table S19). The vast majority of disease-associated genes detected in the cortex were characterized by more than one RNA isoform in both the human (n = 420 [68.9%] genes) and mouse (n = 538 [80.3%] genes) cortex. *TCF4* (autism- and SZ-associated) was the most “isoformic” disease gene in both human (n = 33 isoforms) and mouse (n = 57 isoforms) cortex; of note, both genes have been shown to be key members of transcriptional networks associated with neuropsychiatric disease (Li et al., 2018). Importantly, a large number of the transcripts mapping to disease-associated genes had not been previously annotated in existing databases in human (n = 790 [39.2%] isoforms) and mouse (n = 1,825 [56.7%] isoforms) cortex, identifying novel transcripts that may have potential relevance to understanding neurodegenerative and neuropsychiatric disorders. Interestingly, transcripts from disease-associated genes were characterized by a relatively high level of IR in the human cortex (AD: n = 9 [27.3%]; autism: n = 62 [19.6%]; SZ: n = 75 [26.0%]), with a large proportion of these annotated IR-transcripts being predicted for NMD (AD: n = 4 [44.4% of IR-genes]; autism: n = 24 [38.7% of IR-genes]; SZ: n = 29 [38.6%]; Table S20). There are known links between fusion transcripts and disease (Oliver et al., 2019), and a number of disease-associated genes were involved in fusion events (autism: n = 8, e.g., *ELAC1-SMAD4*; Figure 4A; SZ: n = 5, e.g., *GDPD3-MAPK3*; Figure 4B; autism- and SZ-associated: n = 1, e.g., *FOXG1-LINC01551*; Figure 4C). Given the hypothesized role of neurodevelopment and aging in autism, SZ, and AD, it is notable that we found large differences in isoform diversity between human adult and human fetal cortex for many disease-associated genes (Table S20).

DISCUSSION

We used long-read Iso-Seq to characterize full-length cDNA sequences and generate detailed maps of AS in the human and mouse cortex. We identify considerable RNA isoform diversity among expressed genes in the cortex across both species, including many novel transcripts not present in existing genome annotations. The majority of these isoforms have high coding potential, with the analysis of cortical proteomic data confirming the translation of several novel transcripts. Of note, we detect full-length transcripts from several previously unannotated genes in both the human and mouse cortex and many examples of fusion transcripts incorporating exons from multiple genes. Although global patterns of isoform diversity appear to be similar between both species, we identified some notable exceptions, with certain genes showing species-specific transcriptional complexity. Furthermore, we identify some striking developmental changes in transcript diversity, with certain genes characterized by differential transcript usage between fetal and adult cortex. Importantly, we show that genes associated with autism, SZ, and AD are characterized by considerable RNA isoform diversity, identifying novel transcripts that might play a role in pathology. Our data confirm the importance of AS in the cortex and highlight its role as an important mechanism underpinning gene regulation in the brain.

Our findings highlight the power of long-read sequencing approaches for transcriptional profiling. By generating reads spanning entire transcripts, it is possible to systematically

characterize the repertoire of expressed RNA isoforms and fully assess the prevalence of AS. To our knowledge, our analysis represents the most comprehensive characterization of full-length transcripts and isoform diversity in the cerebral cortex yet undertaken. Several findings are particularly notable. First, we highlight that existing gene annotations are incomplete and that novel transcripts are likely to exist for a large proportion of expressed genes. Our data show examples of novel exons and even entire genes not currently annotated in existing databases. Importantly, it has been shown that such incomplete annotation has a disproportionate impact on our understanding of Mendelian and complex neurogenetic disorders (Zhang et al., 2020). Our resource enhances our understanding of the repertoire of expressed transcripts in the cerebral cortex. Second, we show that read-through transcripts (or gene fusion transcripts)—formed when exons from two genes fuse together—occur at detectable levels in the cortex. Although many of these fusion transcripts appear to be associated with NMD, some have the potential to be translated into proteins or may have a regulatory effect at the RNA level. Despite gene-fusion transcripts having a well-documented role in several human cancers (Futreal et al., 2004), the systematic analysis of gene fusion and read-through transcripts has been limited to date given the limitations of existing short-read sequencing technologies (Haas et al., 2019). Our data support recent data suggesting that read-through transcripts occur naturally (Mehani et al., 2020) and suggest that some fusion transcripts may have protein-coding potential, with important implications for brain disease. Third, we are able to highlight the significant extent to which AS events contribute to isoform diversity in the cortex. In particular, we show that IR is a relatively common form of AS in the cortex that is associated with reduced expression and NMD. Importantly, IR was more prevalent in the human fetal cortex than adult cortex, supporting previous studies that implicate a role of IR in the developmental regulation of gene transcription in the brain (Ameur et al., 2011). Finally, we highlight major developmental changes in cortical isoform abundance in the human brain. In particular, we identify striking examples of transcript usage between fetal and adult cortex and also significant differences in isoform expression between different regions of the human brain.

Our results should be interpreted in the context of several limitations. First, we profiled tissue from a relatively small number of human and mouse donors. Although we found highly consistent patterns of AS across these biological replicates and rarefaction curves confirmed our sequencing dataset was close to saturation, we were unable to explore inter-individual variation in AS. Recent studies have highlighted considerable evidence for genetic influences on isoform diversity in the human cortex, with splicing quantitative trait loci (sQTL) widely implicated in health and disease (Takata, Matsumoto and Kato, 2017). Future work will aim to extend our analyses to larger numbers of samples to explore population-level variation in transcript abundance in the cerebral cortex and differences associated with pathology. Second, despite the advantages of long-read sequencing approaches for the characterization of novel full-length transcripts, these methods are often assumed to be less quantitative than traditional short-read RNA sequencing methods (Zhao et al., 2019). We implemented a stringent QC pipeline and undertook considerable filtering of our data, finding high consistency across biological replicates and validating our findings using complementary approaches (i.e., nanopore sequencing, RNA-seq, and by comparison to existing genomic databases). We show that transcriptional profiles generated using Iso-Seq reflect those expected from the tissues we assessed (i.e., the cerebral cortex), and we found a strong correlation with both gene- and transcript-level expressions measured using short-read RNA-seq on the same samples. We also observed a strong correlation between expected and detected levels of ERCC spike-in control molecules, highlighting the power of Iso-Seq to accurately quantify the abundance of highly expressed transcripts. Given that we have adopted stringent QC approaches, many true transcripts from our final dataset—particularly lowly-expressed transcripts—are likely to have been filtered out. Our analyses therefore probably underestimate the extent of RNA isoform diversity in the cerebral cortex so we also provide a less conservatively filtered dataset for download from our online track hub. Third, our analyses were performed on "bulk" cortex tissue containing a heterogeneous mix of neurons, oligodendrocytes, and other glial cell types. It is likely that these different cell types express a specific repertoire of RNA isoforms, and we are not able to explore these differences in our data. Of note, novel approaches for using long-read sequencing approaches in single cells will enable a more granular approach to exploring transcript diversity in the cortex. Although such approaches are currently limited by technological and analytical constraints, a recent study used long-read transcriptome sequencing to identify cell-type-specific transcript diversity in the mouse hippocampus and prefrontal cortex (Joglekar et al., 2021). Finally, although we explored the extent to which novel transcripts contained ORFs, the extent to which they are actually translated and contribute to cortical proteomic diversity is not known.

In summary, our data confirm the importance of AS and AF exon usage in the cerebral cortex, dramatically increasing transcriptional diversity and representing an important mechanism underpinning gene regulation in the brain. We highlight the power of long-read sequencing for completing our understanding of human and mouse gene annotation, and our transcript annotations, isoform data, and Iso-Seq analysis pipeline are available as a resource to the research community.

STAR★METHODS

Detailed methods are provided in the online version of this paper and include the following:

- KEY RESOURCES TABLE
- RESOURCE AVAILABILITY
  - Lead contact
  - Materials availability
  - Data and code availability
- EXPERIMENTAL MODEL AND SUBJECT DETAILS
- METHOD DETAILS
  - Brain samples

CellPress
OPEN ACCESS

- ○ Whole transcriptome Iso-seq library preparation and SMRT sequencing
- ○ RNA-seq library preparation and Illumina sequencing
- ○ ONT library preparation, sequencing and data processing
- ● QUANTIFICATION AND STATISTICAL ANALYSIS
  - ○ SMRT sequencing quality control (QC) and data processing
  - ○ RNA-seq QC and data processing
  - ○ Transcriptome annotation and filtering
  - ○ Proteomic analysis of novel isoforms
  - ○ Comparison of RNA-Seq and Iso-Seq expression data
  - ○ Comparison of Iso-Seq transcripts with those identified using ONT nanopore sequencing
  - ○ Gene ontology analysis
  - ○ Comparison of human and mouse cortical transcripts
  - ○ Comparison of different human Iso-Seq datasets
  - ○ Validation of transcriptome landscape
  - ○ Generation of web resources
- ● ADDITIONAL RESOURCES

SUPPLEMENTAL INFORMATION

Supplemental information can be found online at https://doi.org/10.1016/j.celrep.2021.110022.

ACKNOWLEDGMENTS

This work was supported by a grant from the Simons Foundation for Autism Research (SFARI) (grant number 573312, awarded to J.M.), a grant from the UK Medical Research (grant MR/R005176/1, awarded to J.M.), the National Institute of Mental Health (grants R01MH121521, R01MH123922, and P50HD103557 to M.J.G.), and a SFARI Bridge to Independence Award (to M.J.G.). S.K.L. is funded by a UK Medical Research Council CASE PhD studentship. The human fetal material was provided by the Human Developmental Biology Resource (funded by a MRC/Wellcome Trust grant (099175/Z/12/Z)https://www.hdbr.org. Sequencing infrastructure was supported by a Wellcome Trust Multi User Equipment Award (WT101650MA, awarded to J.M.) and Medical Research Council (MRC) Clinical Infrastructure Funding (MR/M008924/1, awarded to J.M.). We acknowledge the help of R. Flynn from the University of Exeter Medical School for his help with screening fusion transcripts.

AUTHOR CONTRIBUTIONS

A.R.J. and S.K.L. conducted long-read sequencing experiments. A.R.J., I.C., and K.M. conducted short-read RNA-seq experiments. J.P.D. provided technical assistance with sample preparation. N.J.B. provided fetal cortex RNA samples. K.M. advised on library preparation and aspects of sequencing. Z.A. provided mouse cortex tissue. J.M., E.H., L.S., and E.L.D. obtained funding. J.M. and A.R.J. designed the study. A.R.J. and S.K.L. undertook primary data analyses and bioinformatics, with analytical and computational input from E.H., P.O., and M.J.G. G.M.S., B.T.J., and E.D.J. undertook analysis of protein isoform data. C.J. helped with development of the data visualization tool. M.J.G., S.P., L.S., E.L.D., D.A.C., and N.J.B. helped interpret the results. A.R.J., S.K.L., and J.M. drafted the manuscript. All authors read and approved the final submission.

DECLARATION OF INTERESTS

Z.A. and D.A.C. were full-time employees of Eli Lilly & Company, Ltd., and E.T. was a full-time employee of PacBio at the time this work was performed. All other authors declare no competing interests.

Received: October 14, 2020
Revised: July 30, 2021
Accepted: October 28, 2021
Published: November 16, 2021

REFERENCES

Akiva, P., Toporik, A., Edelheit, S., Peretz, Y., Diber, A., Shemesh, R., Novik, A., and Sorek, R. (2006). Transcription-mediated gene fusion in the human genome. Genome Res. *16*, 30–36.

Amarasinghe, S.L., Su, S., Dong, X., Zappia, L., Ritchie, M.E., and Gouil, Q. (2020). Opportunities and challenges in long-read sequencing data analysis. Genome Biol. *21*, 30.

Ameur, A., Zaghlool, A., Halvardson, J., Wetterbom, A., Gyllensten, U., Cavelier, L., and Feuk, L. (2011). Total RNA sequencing reveals nascent transcription and widespread co-transcriptional splicing in the human brain. Nat. Struct. Mol. Biol. *18*, 1435–1440.

Andrews, S.J., Fulton-Howard, B., and Goate, A. (2020). Interpretation of risk loci from genome-wide association studies of Alzheimer's. The Lancet *19*, 326–335.

Bekris, L.M., Yu, C.E., Bird, T.D., and Tsuang, D.W. (2010). Genetics of Alzheimer disease. J. Geriatr. Psychiatry Neurol. *23*, 213–227.

Braunschweig, U., Barbosa-Morais, N.L., Pan, Q., Nachman, E.N., Alipanahi, B., Gonatopoulos-Pournatzis, T., Frey, B., Irimia, M., and Blencowe, B.J. (2014). Widespread intron retention in mammals functionally tunes transcriptomes. Genome Res. *24*, 1774–1786.

Bray, N.L., Pimentel, H., Melsted, P., and Pachter, L. (2016). Near-optimal probabilistic RNA-seq quantification. Nat. Biotechnol. *34*, 525–527.

Castanho, I., Murray, T.K., Hannon, E., Jeffries, A., Walker, E., Laing, E., Baulf, H., Harvey, J., Bradshaw, L., Randall, A., et al. (2020). Transcriptional Signatures of Tau and Amyloid Neuropathology. Cell Rep. *30*, 2040–2054.e5.

Conesa, A., Madrigal, P., Tarazona, S., Gomez-Cabrero, D., Cervera, A., McPherson, A., Wojciech Szcześniak, M., Gaffney, D.J., Elo, L.L., Zhang, X., and Mortazavi, A. (2016). A survey of best practices for RNA-seq data analysis. Genome Biol. *17*, 13.

Cummings, B.B., Karczewski, K.J., Kosmicki, J.A., Seaby, E.G., Watts, N.A., Singer-Berk, M., Mudge, J.M., Karjalainen, J., Satterstrom, F.K., O'Donnell-Luria, A.H., et al.; Genome Aggregation Database Production Team; Genome Aggregation Database Consortium (2020). Transcript expression-aware annotation improves rare variant interpretation. Nature *581*, 452–458.

Dobin, A., Davis, C.A., Schlesinger, F., Drenkow, J., Zaleski, C., Jha, S., Batut, P., Chaisson, M., and Gingeras, T.R. (2013). STAR: ultrafast universal RNA-seq aligner. Bioinformatics *29*, 15–21.

Eksi, R., Li, H.D., Menon, R., Wen, Y., Omenn, G.S., Kretzler, M., and Guan, Y. (2013). Systematically differentiating functions for alternatively spliced isoforms through integrating RNA-seq data. PLoS Comput. Biol. *9*, e1003314.

Fu, X.D., and Ares, M. (2014). Context-dependent control of alternative splicing by RNA-binding proteins. Nat. Rev. Genet. *15*, 689–701.

Futreal, P.A., Coin, L., Marshall, M., Down, T., Hubbard, T., Wooster, R., Rahman, Z., and Stratton, M.R. (2004). A census of human cancer genes. Nat. Rev. Cancer *4*, 177–183.

Galante, P.A.F., Vidal, D.O., de Souza, J.E., Camargo, A.A., and de Souza, S.J. (2007). Sense-antisense pairs in mammals: functional and evolutionary considerations. Genome Biol. *8*, R40.

Ge, Y., and Porse, B.T. (2014). The functional consequences of intron retention: alternative splicing coupled to NMD as a regulator of gene expression. BioEssays *36*, 236–243.

Gordon, S.P., Tseng, E., Salamov, A., Zhang, J., Meng, X., Zhao, Z., Kang, D., Underwood, J., Grigoriev, I.V., Figueroa, M., et al. (2015). Widespread polycistronic transcripts in fungi revealed by single-molecule mRNA sequencing. PLoS ONE *10*, e0132628.

Grabski, D.F., Broseus, L., Kumari, B., Rekosh, D., Hammarskjold, M.-L., and Ritchie, W. (2021). Intron retention and its impact on gene expression and

protein diversity: A review and a practical guide. Wiley Interdiscip. Rev. RNA *12*, e1631.

GrandPré, T., Nakamura, F., Vartanian, T., and Strittmatter, S.M. (2000). Identification of the Nogo inhibitor of axon regeneration as a Reticulon protein. Nature *403*, 439–444.

GTEx Consortium (2015). The Genotype-Tissue Expression (GTEx) pilot analysis: Multitissue gene regulation in humans. Science *348*, 648–660.

Guttman, M., Russell, P., Ingolia, N.T., Weissman, J.S., and Lander, E.S. (2013). Ribosome profiling provides evidence that large noncoding RNAs do not encode proteins. Cell *154*, 240–251.

Haas, B.J., Dobin, A., Li, B., Stransky, N., Pochet, N., and Regev, A. (2019). Accuracy assessment of fusion transcript detection via read-mapping and de novo fusion transcript assembly-based methods. Genome Biol. *20*, 213.

Hug, N., Longman, D., and Cáceres, J.F. (2015). Mechanism and regulation of the nonsense-mediated decay pathway. Nucleic Acids Res. *44*, 1483–1495.

Jacob, A.G., and Smith, C.W.J. (2017). Intron retention as a component of regulated gene expression programs. Hum. Genet. *136*, 1043–1057.

Joglekar, A., Prjibelski, A., Mahfouz, A., Collier, P., Lin, S., Schlusche, A.K., Marrocco, J., Williams, S.R., Haase, B., Hayes, A., et al. (2021). A spatially resolved brain region- and cell type-specific isoform atlas of the postnatal mouse brain. Nat. Commun. *12*, 463.

Kageyama, Y., Kondo, T., and Hashimoto, Y. (2011). Coding vs non-coding: Translatability of short ORFs found in putative non-coding transcripts. Biochimie *93*, 1981–1986.

Kim, M.S., Pinto, S.M., Getnet, D., Nirujogi, R.S., Manda, S.S., Chaerkady, R., Madugundu, A.K., Kelkar, D.S., Isserlin, R., Jain, S., et al. (2014). A draft map of the human proteome. Nature *509*, 575–581.

Kuleshov, M.V., Jones, M.R., Rouillard, A.D., Fernandez, N.F., Duan, Q., Wang, Z., Koplev, S., Jenkins, S.L., Jagodnik, K.M., Lachmann, A., et al. (2016). Enrichr: a comprehensive gene set enrichment analysis web server 2016 update. Nucleic Acids Res. *44* (*W1*), W90–W97.

Kuo, R.I., Tseng, E., Eory, L., Paton, I.R., Archibald, A.L., and Burt, D.W. (2017). Normalized long read RNA sequencing in chicken reveals transcriptome complexity similar to human. BMC Genomics *18*, 323.

Li, H. (2018). Minimap2: pairwise alignment for nucleotide sequences. Bioinformatics *34*, 3094–3100.

Li, M., Santpere, G., Kawasawa, Y.I., Evgrafov, O.V., Gulden, F.O., Pochareddy, S., Sunkin, S.M., Li, Z., Shin, Y., Zhu, Y., et al. (2018). Integrative functional genomic analysis of human brain development and neuropsychiatric risks. Science *362*, eaat7615.

Liu, S.J., Nowakowski, T.J., Pollen, A.A., Lui, J.H., Horlbeck, M.A., Attenello, F.J., He, D., Weissman, J.S., Kriegstein, A.R., Diaz, A.A., and Lim, D.A. (2016). Single-cell analysis of long non-coding RNAs in the developing human neocortex. Genome Biol. *17*, 67.

Lizio, M., Abugessaisa, I., Noguchi, S., Kondo, A., Hasegawa, A., Hon, C.C., de Hoon, M., Severin, J., Oki, S., Hayashizaki, Y., et al. (2019). Update of the FANTOM web resource: expansion to provide additional transcriptome atlases. Nucleic Acids Res. *47* (*D1*), D752–D758.

Mazin, P., Xiong, J., Liu, X., Yan, Z., Zhang, X., Li, M., He, L., Somel, M., Yuan, Y., Phoebe Chen, Y.P., et al. (2013). Widespread splicing changes in human brain development and aging. Mol. Syst. Biol. *9*, 633.

McCartney, A.M., Hyland, E.M., Cormican, P., Moran, R.J., Webb, A.E., Lee, K.D., Hernandez-Rodriguez, J., Prado-Martinez, J., Creevey, C.J., Aspden, J.L., et al. (2019). Gene Fusions Derived by Transcriptional Readthrough are Driven by Segmental Duplication in Human. Genome Biol. Evol. *11*, 2678–2690.

Mehani, B., Narta, K., Paul, D., Raj, A., Kumar, D., Sharma, A., Kaurani, L., Nayak, S., Dash, D., Suri, A., et al. (2020). Fusion transcripts in normal human cortex increase with age and show distinct genomic features for single cells and tissues. Sci. Rep. *10*, 1368.

Mills, J.D., and Janitz, M. (2012). Alternative splicing of mRNA in the molecular pathology of neurodegenerative diseases. Neurobiol. Aging *33*, 1012.e11–1012.e24.

Nellore, A., Jaffe, A.E., Fortin, J.P., Alquicira-Hernández, J., Collado-Torres, L., Wang, S., Phillips, R.A., III, Karbhari, N., Hansen, K.D., Langmead, B., and Leek, J.T. (2016). Human splicing diversity and the extent of unannotated splice junctions across human RNA-seq samples on the Sequence Read Archive. Genome Biol. *17*, 266.

Oliver, G.R., Tang, X., Schultz-Rogers, L.E., Vidal-Folch, N., Jenkinson, W.G., Schwab, T.L., Gaonkar, K., Cousin, M.A., Nair, A., Basu, S., et al. (2019). A tailored approach to fusion transcript identification increases diagnosis of rare inherited disease. PLoS ONE *14*, e0223337.

Pan, Q., Saltzman, A.L., Kim, Y.K., Misquitta, C., Shai, O., Maquat, L.E., Frey, B.J., and Blencowe, B.J. (2006). Quantitative microarray profiling provides evidence against widespread coupling of alternative splicing with nonsense-mediated mRNA decay to control gene expression. Genes Dev. *20*, 153–158.

Pan, Q., et al. (2009). Deep surveying of alternative splicing complexity in the human transcriptome by high-throughput sequencing.Deep surveying of alternative splicing complexity in the human transcriptome by high-throughput sequencing - Sup Mat. Nat. Genet. *41*, 762.

Pardiñas, A.F., Holmans, P., Pocklington, A.J., Escott-Price, V., Ripke, S., Carrera, N., Legge, S.E., Bishop, S., Cameron, D., Hamshere, M.L., et al.; GERAD1 Consortium; CRESTAR Consortium (2018). Common schizophrenia alleles are enriched in mutation-intolerant genes and in regions under strong background selection. Nat. Genet. *50*, 381–389.

Parikshak, N.N., Swarup, V., Belgard, T.G., Irimia, M., Ramaswami, G., Gandal, M.J., Hartl, C., Leppa, V., Ubieta, L.T., Huang, J., et al. (2016). Genome-wide changes in lncRNA, splicing, and regional gene expression patterns in autism. Nature *540*, 423–427.

Park, E., Pan, Z., Zhang, Z., Lin, L., and Xing, Y. (2018). The Expanding Landscape of Alternative Splicing Variation in Human Populations. Am. J. Hum. Genet. *102*, 11–26.

Pertea, G., and Pertea, M. (2020). GFF Utilities: GffRead and GffCompare. F1000Res. *2020*, 9.

Pertea, M., Pertea, G.M., Antonescu, C.M., Chang, T.C., Mendell, J.T., and Salzberg, S.L. (2015). StringTie enables improved reconstruction of a transcriptome from RNA-seq reads. Nat. Biotechnol. *33*, 290–295.

Pertea, M., Shumate, A., Pertea, G., Varabyou, A., Breitwieser, F.P., Chang, Y.C., Madugundu, A.K., Pandey, A., and Salzberg, S.L. (2018). CHESS: a new human gene catalog curated from thousands of large-scale RNA sequencing experiments reveals extensive transcriptional noise. Genome Biol. *19*, 208.

Pine, P.S., Munro, S.A., Parsons, J.R., McDaniel, J., Lucas, A.B., Lozach, J., Myers, T.G., Su, Q., Jacobs-Helber, S.M., and Salit, M. (2016). Evaluation of the External RNA Controls Consortium (ERCC) reference material using a modified Latin square design. BMC Biotechnol. *16*, 54.

Piovesan, A., Antonaros, F., Vitale, L., Strippoli, P., Pelleri, M.C., and Caracausi, M. (2019). Human protein-coding genes and gene feature statistics in 2019. BMC Res. Notes *12*, 315.

Prakash, T., Sharma, V.K., Adati, N., Ozawa, R., Kumar, N., Nishida, Y., Fujikake, T., Takeda, T., and Taylor, T.D. (2010). Expression of conjoined genes: another mechanism for gene regulation in eukaryotes. PLoS ONE *5*, e13284.

Raj, B., and Blencowe, B.J. (2015). Alternative Splicing in the Mammalian Nervous System: Recent Insights into Mechanisms and Functional Roles. Neuron *87*, 14–27.

Raj, T., Li, Y.I., Wong, G., Humphrey, J., Wang, M., Ramdhani, S., Wang, Y.C., Ng, B., Gupta, I., Haroutunian, V., et al. (2018). Integrative transcriptome analyses of the aging brain implicate altered splicing in Alzheimer's disease susceptibility. Nat. Genet. *50*, 1584–1592.

Sims, R., Hill, M., and Williams, J. (2020). The multiplex model of the genetics of Alzheimer's disease. Nat. Neurosci. *23*, 311–322.

Solntsev, S.K., Shortreed, M.R., Frey, B.L., and Smith, L.M. (2018). Enhanced Global Post-translational Modification Discovery with MetaMorpheus. J. Proteome Res. *17*, 1844–1851.

Statello, L., Guo, C.-J., Chen, L.-L., and Huarte, M. (2021). Gene regulation by long non-coding RNAs and its biological functions. Nat. Rev. Mol. Cell Biol. *22*, 96–118.

Steijger, T., Abril, J.F., Engström, P.G., Kokocinski, F., Hubbard, T.J., Guigó, R., Harrow, J., and Bertone, P.; RGASP Consortium (2013). Assessment of transcript reconstruction methods for RNA-seq. Nat. Methods *10*, 1177–1184.

Takata, A., Matsumoto, N., and Kato, T. (2017). Genome-wide identification of splicing QTLs in the human brain and their enrichment among schizophrenia-associated loci. Nat. Commun. *8*, 14519.

Tam, V., Patel, N., Turcotte, M., Bossé, Y., Paré, G., and Meyre, D. (2019). Benefits and limitations of genome-wide association studies. Nat. Rev. Genet. *20*, 467–484.

Tan, M.C., Widagdo, J., Chau, Y.Q., Zhu, T., Wong, J.J., Cheung, A., and Anggono, V. (2017). The activity-induced long non-coding RNA Meg3 modulates AMPA receptor surface expression in primary cortical neurons. Front. Cell. Neurosci. *11*, 124.

Tang, B.L. (2020). Vesicle transport through interaction with t-SNAREs 1a (Vti1a)'s roles in neurons. Heliyon *6*, e04600.

Tardaguila, M., de la Fuente, L., Marti, C., Pereira, C., Pardo-Palacios, F.J., Del Risco, H., Ferrell, M., Mellado, M., Macchietto, M., Verheggen, K., et al. (2018). Corrigendum: SQANTI: extensive characterization of long-read transcript sequences for quality control in full-length transcriptome identification and quantification. Genome Res. *28*, 1096.

Tollervey, J.R., Wang, Z., Hortobágyi, T., Witten, J.T., Zarnack, K., Kayikci, M., Clark, T.A., Schweitzer, A.C., Rot, G., Curk, T., et al. (2011). Analysis of alternative splicing associated with aging and neurodegeneration in the human brain. Genome Res. *21*, 1572–1582.

Trincado, J.L., Entizne, J.C., Hysenaj, G., Singh, B., Skalic, M., Elliott, D.J., and Eyras, E. (2018). SUPPA2: fast, accurate, and uncertainty-aware differential splicing analysis across multiple conditions. Genome Biol. *19*, 40.

Ule, J., and Blencowe, B.J. (2019). Alternative Splicing Regulatory Networks: Functions, Mechanisms, and Evolution. Mol. Cell *76*, 329–345.

Wan, L., and Dreyfuss, G. (2017). Splicing-Correcting Therapy for SMA. Cell *170*, 5.

Wang, G.S., and Cooper, T.A. (2007). Splicing in disease: disruption of the splicing code and the decoding machinery. Nat. Rev. Genet. *8*, 749–761.

Wang, E.T., Sandberg, R., Luo, S., Khrebtukova, I., Zhang, L., Mayr, C., Kingsmore, S.F., Schroth, G.P., and Burge, C.B. (2008). Alternative isoform regulation in human tissue transcriptomes. Nature *456*, 470–476.

Wang, L., Park, H.J., Dasari, S., Wang, S., Kocher, J.P., and Li, W. (2013). CPAT: Coding-Potential Assessment Tool using an alignment-free logistic regression model. Nucleic Acids Res. *41*, e74.

Wang, B., Tseng, E., Regulski, M., Clark, T.A., Hon, T., Jiao, Y., Lu, Z., Olson, A., Stein, J.C., and Ware, D. (2016). Unveiling the complexity of the maize transcriptome by single-molecule long-read sequencing. Nat. Commun. *7*, 11708.

Wang, B., Kumar, V., Olson, A., and Ware, D. (2019). Reviving the Transcriptome Studies: An Insight Into the Emergence of Single-Molecule Transcriptome Sequencing. Front. Genet. *10*, 384.

Yang, X., Coulombe-Huntington, J., Kang, S., Sheynkman, G.M., Hao, T., Richardson, A., Sun, S., Yang, F., Shen, Y.A., Murray, R.R., et al. (2016). Widespread Expansion of Protein Interaction Capabilities by Alternative Splicing. Cell *164*, 805–817.

Zhang, D., Guelfi, S., Garcia-Ruiz, S., Costa, B., Reynolds, R.H., D'Sa, K., Liu, W., Courtin, T., Peterson, A., Jaffe, A.E., et al. (2020). Incomplete annotation has a disproportionate impact on our understanding of Mendelian and complex neurogenetic disorders. Sci. Adv. *6*, eaay8299.

Zhao, L., Zhang, X., Kohnen, M.V., Prasad, K.V.S.K., Gu, L., and Reddy, A.S.N. (2019). Analysis of transcriptome and epitranscriptome in plants using pacbio iso-seq and nanopore-based direct RNA sequencing. Front. Genet. *10*, 253.

STAR★METHODS

KEY RESOURCES TABLE

REAGENT or RESOURCE	SOURCE	IDENTIFIER
**Biological samples**		
Human brain tissue	This paper	N/A
Mouse brain tissue	This paper	N/A
**Critical commercial assays**		
TruSeq Stranded mRNA Sample Prep Kit	Illumina	Cat#20020595
SMARTer PCR cDNA Synthesis Kit	Clontech	Cat#634925
SMRTbell Template Prep Kit	Pacific Biosciences	Cat#100-222-300
Sequel Binding Kit	Pacific Biosciences	Cat#101-029-000
PCR Barcoding Kit	Oxford Nanopore Technology	Cat#SQK-PCB109
**Deposited data**		
Raw Human Iso-Seq data	This paper	SRA: PRJNA664117
Raw Mouse Iso-Seq data	This paper	SRA: PRJNA663877
Visualization of detected full-length transcripts (Genome browser track hub)	This paper	http://genome.ucsc.edu/cgi-bin/hgTracks?hubUrl=http://genome.exeter.ac.uk/hub/hub.txt
Isoform Viewer Resource	This paper	http://genome.exeter.ac.uk/build/index.html
FANTOM5 CAGE database	(Lizio et al., 2019)	https://fantom.gsc.riken.jp/5/
Intropolis junction database	Nellore et al., 2016	https://github.com/nellore/intropolis
Human reference genome Release 38, GRCh38	GENCODE	https://www.gencodegenes.org/human/
Mouse reference genome Release 22, GRCm38	GENCODE	https://www.gencodegenes.org/mouse/release_M22.html
MS-based human adult and fetal proteomic dataset	Kim et al., 2014	PXD000561
RNA-Seq Mouse	Castanho et al., 2020	N/A
RNA-Seq Fetal	This Paper	N/A
PolyA motif list	Elizabeth Tseng	https://github.com/Magdoll/SQANTI2
**Software and algorithms**		
Iso-Seq3	Wang et al., 2016	https://github.com/PacificBiosciences/IsoSeq
Minimap2	Li, 2018	https://github.com/lh3/minimap2
Cupcake	Elizabeth Tseng	https://github.com/Magdoll/cDNA_Cupcake
SQANTI2	Tardaguila et al., 2018	https://github.com/Magdoll/SQANTI2
TAMA	Kuo et al., 2017	https://github.com/GenomeRIK/tama
SUPPA2	Trincado et al., 2018	https://github.com/comprna/SUPPA
Pychopper/Pinfish	Oxford Nanopore Technologies	https://github.com/nanoporetech/pipeline-pinfish-analysis
CPAT	Wang et al., 2013	https://github.com/liguowang/cpat
MetaMorpheus	Solntsev et al., 2018	https://github.com/smith-chem-wisc/MetaMorpheus
STAR	Dobin et al., 2013	https://github.com/alexdobin/STAR
Stringtie	Pertea et al., 2015	https://github.com/gpertea/stringtie
Kallisto	Bray et al., 2016	https://github.com/pachterlab/kallisto

(*Continued on next page*)

Continued

REAGENT or RESOURCE	SOURCE	IDENTIFIER
GffCompare	Pertea and Pertea, 2020	https://ccb.jhu.edu/software/stringtie/gffcompare.shtml
EnrichR	Kuleshov et al., 2016	https://maayanlab.cloud/Enrichr/
Other		
Supporting Code	This paper	https://github.com/SziKayLeung/Whole_Transcriptome_Paper DOI: 10.5281/zenodo.5588498
Resource Website for publication	This paper	http://genome.exeter.ac.uk/BrainIsoforms.html

RESOURCE AVAILABILITY

Lead contact

Further information and requests for resources and reagents should be directed to and will be fulfilled by the Lead Contact, Professor Jonathan Mill (J.mill@exeter.ac.uk).

Materials availability

This study did not generate any new unique reagents.

Data and code availability

Raw PacBio Iso-Seq data have been deposited in the Sequence Read Archive (SRA) database (https://www.ncbi.nlm.nih.gov/sra) under accession numbers PRJNA664117 (human cortex) and PRJNA663877 (mouse cortex). UCSC genome browser tracks of our processed Iso-Seq data (filtered and unfiltered) together with a visual database of cortical isoforms are available at: http://genome.exeter.ac.uk/BrainIsoforms.html.

All original code supporting this study is available at https://github.com/SziKayLeung/Whole_Transcriptome_Paper (https://doi.org/10.5281/zenodo.5588498).

Any additional information required to reanalyze the data reported in this work paper is available from the Lead Contact upon request.

EXPERIMENTAL MODEL AND SUBJECT DETAILS

Adult human prefrontal cortex tissue (n = 4) was obtained from the MRC London Neurodegenerative Diseases Brain Bank (https://www.kcl.ac.uk/neuroscience/facilities/brain-bank). Demographic (including gender and age) data for each donor is detailed in Table S1. Subjects were approached in life for written consent for brain banking, and all tissue donations were collected and stored following legal and ethical guidelines (NHS reference number 08/MRE09/38; the UK Human Tissue Authority HTA license number 12293). Fetal human brain tissue (n = 3) from three brain regions (frontal cortex, hippocampus, and striatum) was obtained from the Human Developmental Biological Resource (HDBR) (https://www.hdbr.org). Ethical approval for the HDBR was granted by the Royal Free Hospital research ethics committee under reference 08/H0712/34 and HTA material storage license 12220. Mouse entorhinal cortex tissue was dissected from twelve female mice in accordance with the UK Animals (Scientific Procedures) Act 1986 and with approval of the local Animal Welfare and Ethical Review Board. Mice were bred and delivered to Eli Lilly and Company (Windlesham, UK) by Envigo (Loughborough, UK), where animals were housed under standard conditions (constant temperature and humidity) with a 12h light/dark cycle in individually ventilated cages (up to 5 animals per cage). All animal procedures were carried out at Eli Lilly and Company, in accordance with the UK Animals (Scientific Procedures) Act 1986 and with approval of the local Animal Welfare and Ethical Review Board.

METHOD DETAILS

Brain samples

For each human sample, ~20mg of flash frozen tissue was homogenized in Trizol (Thermo Fisher Scientific, UK) and RNA was isolated using Direct-zol columns (Zymo, USA). For each mouse sample, RNA was isolated using the AllPrep DNA/RNA Mini Kit (QIAGEN, UK) from ~5mg tissue. RNA samples were quantified using the Nanodrop 1000 spectrophotometer and RNA integrity numbers (RIN) derived using a Bioanalyzer 2100 (Agilent, UK). Additional details on mouse breeding conditions can be found in, and further details on each individual sample used in this study are provided in Table S1.

Whole transcriptome Iso-seq library preparation and SMRT sequencing

First strand cDNA synthesis was performed on ~1μg RNA using the SMARTer PCR cDNA Synthesis Kit (Clontech, UK), with the addition of External RNA Controls Consortium (ERCC) standards (Pine et al., 2016) to a subset of mouse cortex samples (n = 10), followed by PCR amplification with PrimeSTAR GXL DNA Polymerase (Clontech, UK). Optimal PCR cycle number was determined through collection of 5μl aliquots during every two cycles of a test PCR and assessment using 1% agarose gel electrophoresis. Large-scale PCR was subsequently performed using the optimal number of cycles and the resulting amplicons divided into two fractions and purified with 0.4X and 1X Ampure PB beads (PacBio, USA). Quantification and size distribution of each fraction was then determined using the Qubit DNA High sensitivity assay (Invitrogen, UK) and Bioanalyzer 2100 (Agilent, UK). The two fractions were recombined at equimolar quantities and library preparation performed using SMRTbell Template Prep Kit v1.0 (PacBio, USA). Sequencing was performed on the PacBio Sequel 1M SMRT cell. Samples were processed using either the version 3 chemistry (parameters: diffusion loading at 5pM, pre-extension 4 hours, Capture time 20 hours) or version 2.1 chemistry (parameters: magbead loading at 50pM with a 2 hour pre-extension and 10 hour capture).

RNA-seq library preparation and Illumina sequencing

RNA from a subset of human fetal (n = 3) and mouse (n = 12) cortex tissue samples was prepared using the TruSeq Stranded mRNA Sample Prep Kit (Illumina) and subjected to 125bp paired-end sequencing using a HiSeq2500 (Illumina). Briefly, cDNA libraries were prepared from ~450ng of total RNA plus ERCC spike-in synthetic RNA controls (Ambion, dilution 1:100), purified using Ampure XP magnetic beads (Beckman Coulter) and profiled using the D1000 ScreenTape System (Agilent).

ONT library preparation, sequencing and data processing

RNA from a human fetal and human adult cortex sample was profiled using the Oxford Nanopore Technologies (ONT) sequencing platform. Extracted RNA was converted to cDNA using Maxima H Minus RT (Thermo Fisher Scientific) and amplified with 15 cycles of PCR using Takara LA Taq (Clontech). Quantification and size distribution were then determined using the Qubit DNA High sensitivity assay (Invitrogen) and the Bioanalyzer 2100 (Agilent), and library preparation was performed using ONT's PCR barcoding kit (SQK-PCB109). Sequencing was then performed on the ONT PromethION platform using a FLO-PRO002 flow cell, and base-called using *Guppy* (v4.0). Resulting *fastq* files were processed through the *Pychopper/Pinfish* (https://github.com/nanoporetech/pipeline-pinfish-analysis) pipeline to produce both raw and polished transcripts sequences.

QUANTIFICATION AND STATISTICAL ANALYSIS

SMRT sequencing quality control (QC) and data processing

QC of raw reads was performed using SMRT Link Portal v7.0, with subsequent analysis using the *Iso-Seq3.1.2* pipeline (Wang et al., 2016). Briefly, CCS reads were generated from a minimum of 1 pass (*Iso-Seq3 CCS*, v3.4.1). Primers and SMRT adapters were then removed using *Lima* (v1.9) to generate full-length (FL) reads, followed by removal of artificial concatemers reads and trimming of polyA tails in *Iso-Seq3 Refine*. Full-length, non-chimeric (FLNC) reads were then collapsed, according to default parameters in *Iso-Seq3 Cluster*, to high-quality transcripts. *Cupcake's collapse_isoforms_by_sam.py* script was subsequently applied with the following parameters "-c 0.85 -i 0.95–dun-merge-5-shorter" to reduce redundancy (https://github.com/Magdoll/cDNA_Cupcake). High-quality, full-length transcripts were then mapped to the human (hg38, GENCODE v31) or mouse (mm10, GENCODE vM22) reference genome using *minimap2* (v2.17) (Li, 2018) with the following parameters "-ax splice -uf–secondary=no -C5 -O6,24 -B4."

RNA-seq QC and data processing

Raw RNA-Seq sequencing reads, with Phred (Q) ≥ 35, were trimmed (ribosomal sequence removal, quality threshold 20, minimum sequence length 35) using *fastqmcf* (v1.0), yielding a mean untrimmed read depth of ~20 million reads/sample. Subsequent filtered reads were then mapped to the human (hg38) or mouse (mm10) reference genome using *STAR* (v1.9) (Dobin et al., 2013). Gene and transcript expression were determined by aligning merged RNA-Seq reads to RNA isoforms (*Cupcake* collapsed) from Iso-Seq datasets using *Kallisto* (v0.46.0) (Bray et al., 2016) with default parameters as input to *SQANTI2* (https://github.com/Magdoll/SQANTI2). Using mouse RNA-Seq reads, a transcriptome assembly was generated using *Stringtie* (v2.1.4) (Pertea et al., 2015) with mouse reference GENCODE gtf (vM22), annotated and filtered with *SQANTI2* (v7.4) using default parameters.

Transcriptome annotation and filtering

After filtering for partial isoforms including 5′ degradation products using TAMA's script (tama_remove_fragment_models.py) with default parameters (Kuo et al., 2017), isoforms detected using SMRT sequencing were characterized and classified using *SQANTI2* (v7.4) (Tardaguila et al., 2018) in combination with GENCODE (human v31, mouse vM22) comprehensive gene annotation, FANTOM5 CAGE peaks (Lizio et al., 2019) (human – hg38, mouse – mm10), polyA motifs, Intropolis junction dataset (Nellore et al., 2016) or *STAR* output junction file, FL read counts (abundance file), and *Kallisto* counts from mouse and human fetal RNA-Seq data. An isoform was classified as FSM if it aligned with reference genome with the same splice junctions and contained the same number of exons, ISM if it contained fewer 5′ exons than reference genome, NIC if it is a novel isoform containing a combination of known donor or acceptor sites, or NNC if it is a novel isoform with at least one novel donor or acceptor site. Depictions of RNA isoform classifications can be

CellPress
OPEN ACCESS

found in Figure 2A. Potential artifacts such as reverse transcription jumps or intrapriming of intronic lariats were filtered out using the *SQANTI2* filter script with an intrapriming rate of 0.6. Identification of fusion transcripts, intron retention, polyA motifs and proximity to CAGE peaks were defined based on *SQANTI2* filtered isoforms. The occurrence of mutually exclusive exons (MX) and skipped exons (SE) were assessed using *SUPPA2* (Trincado et al., 2018) with the parameter –f *ioe*, intron retention (IR) with *SQANTI2*, and alternative first exons (AF), alternative last exons (AL), alternative 5′ splice sites (A5), and alternative 3′ splice sites (A3) using custom scripts based on splice junction coordinates. Classification of isoforms as lncRNA (long non-coding RNA) was performed by using *SQANTI2* in combination with GENCODE (human - v31, mouse - vM22) long non-coding RNA gene annotation. ORFs were predicted using the CPAT program (v3.0.2) using all default parameters and transcripts were predicted as protein-coding if the coding potential score was > = 0.364 for human and > 0.44 for mouse (Table S21).

Proteomic analysis of novel isoforms

MS-based proteomics data were previously collected on adult and fetal brain (Kim et al., 2014) (PXD accession PXD000561). In this dataset, SDS-PAGE or basic RPLC fractions were analyzed on a micro-capillary RPLC interfaced to a LTQ-Orbitrap Velos or Elite mass spectrometer. We downloaded the proteomics files (raw Thermo format) and searched the results against a protein database derived from the CPAT ORF predictions on the Iso-Seq adult brain dataset. Standard proteomic analysis of the tryptic and multi-protease datasets was performed using the free and open-source search software program MetaMorpheus (v0.0.316) (Solntsev et al., 2018). The search was conducted with a contaminants database, included in MetaMorpheus, which contains 264 common contaminant proteins frequently found in MS samples.

All spectra files were first converted to MzML format with MSConvert (centroid mode) prior to analysis with MetaMorpheus. All peptide results reported employ a 1% False Discovery Rate (FDR) threshold after target-decoy searching. The output results tables were analyzed using custom python scripts to determine if each identified peptide was present in the GENCODE reference, or, if not, was considered novel. For novel peptide analysis, we manually examined the fragmentation mass spectra (MS2 scans) to confirm the quality of the peptide identification.

Comparison of RNA-Seq and Iso-Seq expression data

Gene and transcript expression between Iso-Seq and RNA-Seq were compared for human and mouse cortex using *SQANTI2* output, with Kallisto expression file as input. Iso-Seq gene expression was determined with the summation of associated transcript FL read counts, with mono-exonic transcripts removed, and normalized to TPM (calculated from FL read counts/total transcriptome counts * 1,000,000). RNA-Seq gene and isoform expression was determined from alignment of RNA-Seq reads to Iso-Seq isoforms, generated from *Cupcake* scripts, using *Kallisto*. For more stringent investigation of the relationship between the gene length, number of exons (determined by representative longest transcript) and the number of transcripts, an Iso-Seq gene expression threshold (> 2.5 Log_{10} TPM) was applied. This threshold was selected based on the gene expression that gave the most statistically-significant correlation between human and mouse isoform number (Table S22).

Comparison of Iso-Seq transcripts with those identified using ONT nanopore sequencing

The human cortex Iso-seq dataset was subsetted to transcripts of interest using the Linux grep command. The resulting GTF file was then examined for overlap with other datasets using *Gffcompare* (Pertea and Pertea, 2020) (https://ccb.jhu.edu/software/stringtie/gffcompare.shtml). Comparisons were made to raw ONT nanopore reads generated in this study.

Gene ontology analysis

EnrichR (Kuleshov et al., 2016) based gene enrichment analysis was performed on three sets of analyses: i) the top 500 most abundantly expressed genes, ii) the top 100 most isoformic genes and iii) genes with intron-retained transcripts in human and mouse Iso-Seq cortical datasets. Iso-Seq gene expression was calculated as described above from summation of associated transcript FL read counts within the *SQANTI2* classification file. The functional categories examined were: GO_Biological_Process, GO_Cellular_Component, GO_Molecular_Function, Panther_2016, Human_Gene_Atlas, ARCHS4_Tissues and GWAS_Catalogue_2019. Mouse_Gene_Atlas was used for the mouse transcriptome.

Comparison of human and mouse cortical transcripts

For appropriate comparison of the Iso-Seq datasets, the mouse gene names were converted to the equivalent homologous human gene names according to mouse genome informatics syntenic gene list (http://www.informatics.jax.org/downloads/reports/HOM_MouseHumanSequence.rpt), considering only mouse-specific homologous genes. 17,042 genes were identified from the list as homologous, of which 267 genes from the human homologous list and 282 genes from the mouse homologous list were removed due to cross genome annotation. This gene set was then used to determine the relationship between human and mouse cortex of Iso-Seq gene expression and number of identified isoforms (Table S5). BLAST analysis of human and novel genes was performed against respective reference genomes (human: hg38, mouse: mm10).

Comparison of different human Iso-Seq datasets

All human cortex samples (human adult = 4 samples, human fetal = 3 samples) and fetal samples (cortex = 3 samples, hippocampus = 2 samples, striatum = 2 samples) were merged and subsequently processed to generate two comprehensive annotated datasets. Full-length read counts from each individual sample and associated SMRT cell were extracted from *Cupcake's* read_stat.txt file and normalized to TPM (calculated from FL read counts/total transcriptome counts * 1,000,000) * 1,000,000). Testing for differential transcript expression between human fetal and adult samples was then performed with a Wilcoxon rank sum test ($p < 0.05$). Differential transcript usage was assessed by identifying instances where at least two transcripts within a gene showed exclusive differential transcript expression in fetal or adult samples respectively, as well as a difference in TPM > 20. Testing for differential transcript usage between fetal brain regions consisted of data from only two SMRT cells per brain region, limiting the power of a Wilcoxon rank sum test. Instead, differential transcript expression and differential transcript usage was based on the following criteria: a minimum fold change of 4 in mean TPM levels and an absolute difference > 20 TPM between respective brain regions.

Validation of transcriptome landscape

The presence of CAGE peaks near novel transcripts were checked, with liftOver (https://genome.ucsc.edu/cgi-bin/hgLiftOver) performed on the mouse dataset to convert mm9 to mm10 genome coordinates.

Generation of web resources

Gene transfer format files (GTF) from *SQANTI2* were processed to a bigGenePred (https://genome.ucsc.edu/goldenPath/help/bigGenePred.html) followed by bed and bigBed format (https://genome.ucsc.edu/goldenPath/help/hubQuickStartSearch.html) to construct the hub. Further annotation enhancements were made to the *bigBed* files through an R script to extract *SQANTI2*-defined structural categories and associated gene names to relabel and color individual transcripts. Separate coloring schemes were also used to indicate the level of expression of each identified transcript.

ADDITIONAL RESOURCES

UCSC genome browser tracks of our processed Iso-Seq data (filtered and unfiltered) together with a visual database of cortical isoforms are available at http://genome.exeter.ac.uk/BrainIsoforms.html and http://genome.exeter.ac.uk/build/index.html.

Cell Reports, Volume *37*

Supplemental information

Full-length transcript sequencing of human and mouse cerebral cortex identifies widespread isoform diversity and alternative splicing

Szi Kay Leung, Aaron R. Jeffries, Isabel Castanho, Ben T. Jordan, Karen Moore, Jonathan P. Davies, Emma L. Dempster, Nicholas J. Bray, Paul O'Neill, Elizabeth Tseng, Zeshan Ahmed, David A. Collier, Erin D. Jeffery, Shyam Prabhakar, Leonard Schalkwyk, Connor Jops, Michael J. Gandal, Gloria M. Sheynkman, Eilis Hannon, and Jonathan Mill

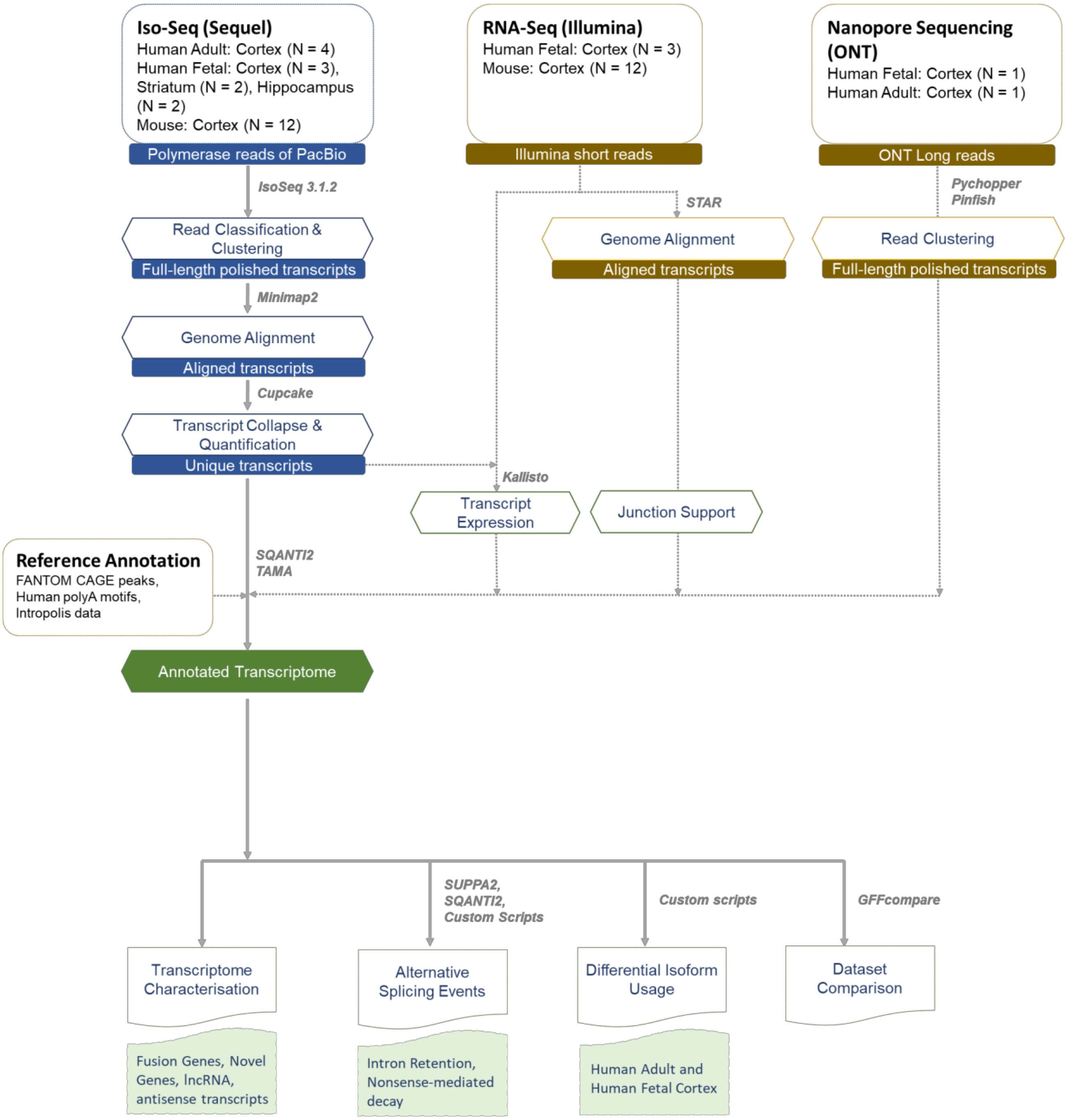

Figure S1: An overview of the analysis pipeline used to generate full-length transcript annotations in human and mouse cerebral cortex samples, related to STAR Methods. Briefly, polymerase reads from PacBio Sequel for each dataset were processed using *Iso-Seq 3.1.2* and *Cupcake* scripts to generate high quality, full-length isoforms. *SQANTI2* was used to fully annotate individual isoforms, with comparison to short-read RNA-Seq, ONT nanopore sequencing, and reference annotations. PacBio – Pacific Biosciences, ONT – Oxford Nanopore Technology

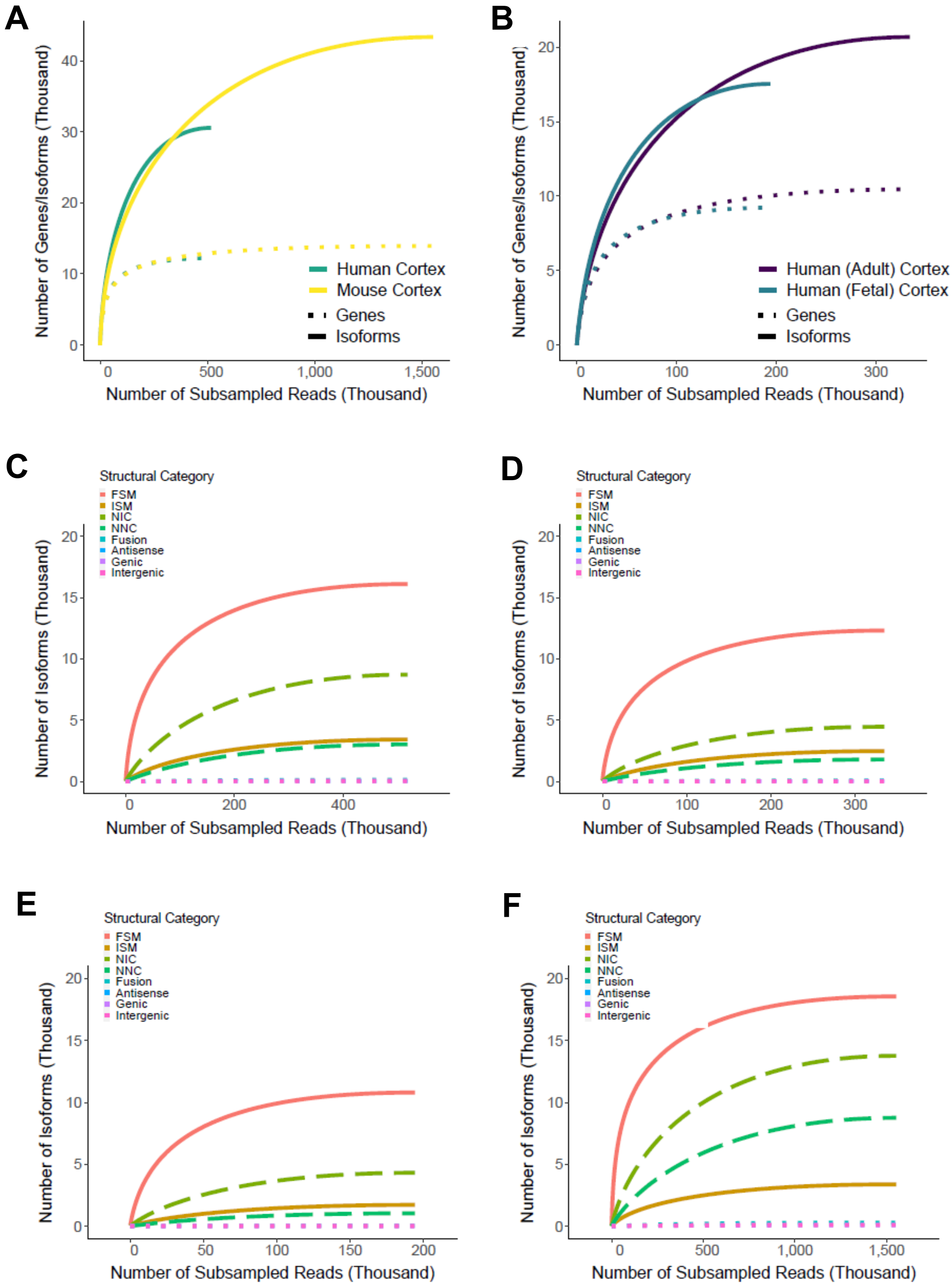

Figure S2: Saturation is reached across all Iso-Seq datasets at the gene and isoform level, related to STAR Methods and Table 1. We subsampled reads to generate rarefaction curves, using *cDNA Cupcake* scripts, at the gene and isoform level. Shown are comparisons between **A)** human (n = 7 biologically independent samples) and mouse cortex (n = 12 biologically independent samples) and **B)** human adult (n = 4 biologically independent samples) and human fetal cortex (n = 3 biologically independent samples). Also shown are rarefaction curves for each *SQANTI2* isoform category in **C)** human cortex, **D)** human adult cortex, **E)** human fetal cortex and **F)** mouse cortex. FSM – Full splice match, ISM – Incomplete Splice Match, NIC – Novel In Catalogue, NNC – Novel Not in Catalogue.

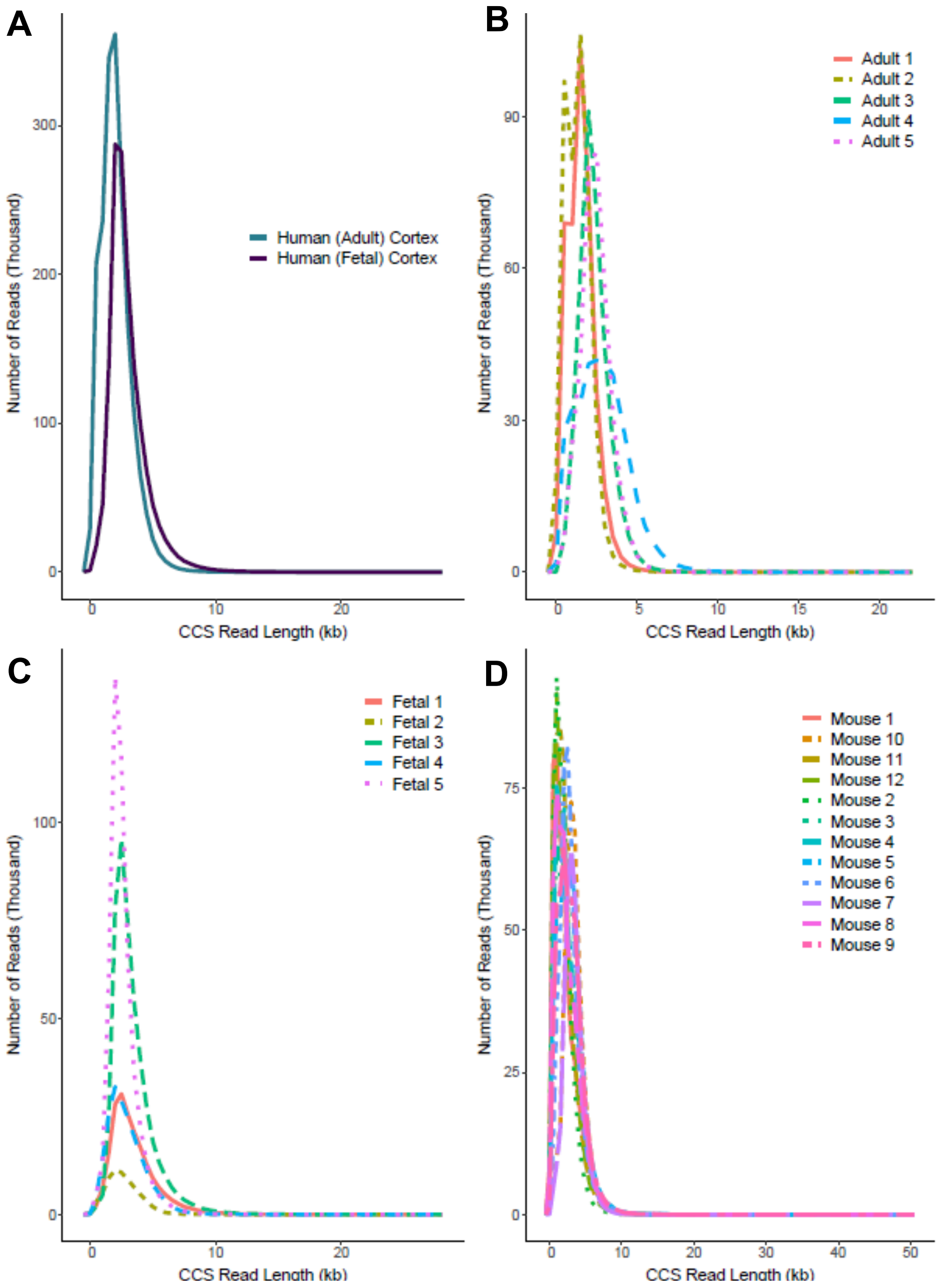

Figure S3: Consensus distribution of CCS read lengths across all cortical samples, related to Figure 1. Shown is data for CCS reads generated from **A)** merging human and adult fetal cortex, and for each sample in **B)** human adult cortex (n = 4 biologically independent samples, n = 5 SMRT cells), **C)** human fetal cortex (n = 3 biologically independent samples, n = 5 SMRT cells) and **D)** mouse cortex (n = 12 biologically independent samples, n = 12 SMRT cells). Several of the human adult and human fetal cortex samples were sequenced more than once to maximize coverage (**Table S1**). Number of CCS reads generated per SMRT cell can be found in **Table S3**. Distribution of CCS read lengths in human and mouse cortex can be found in **Figure 1A**. CCS – Circular consensus sequence. SMRT – Single-molecule real-time

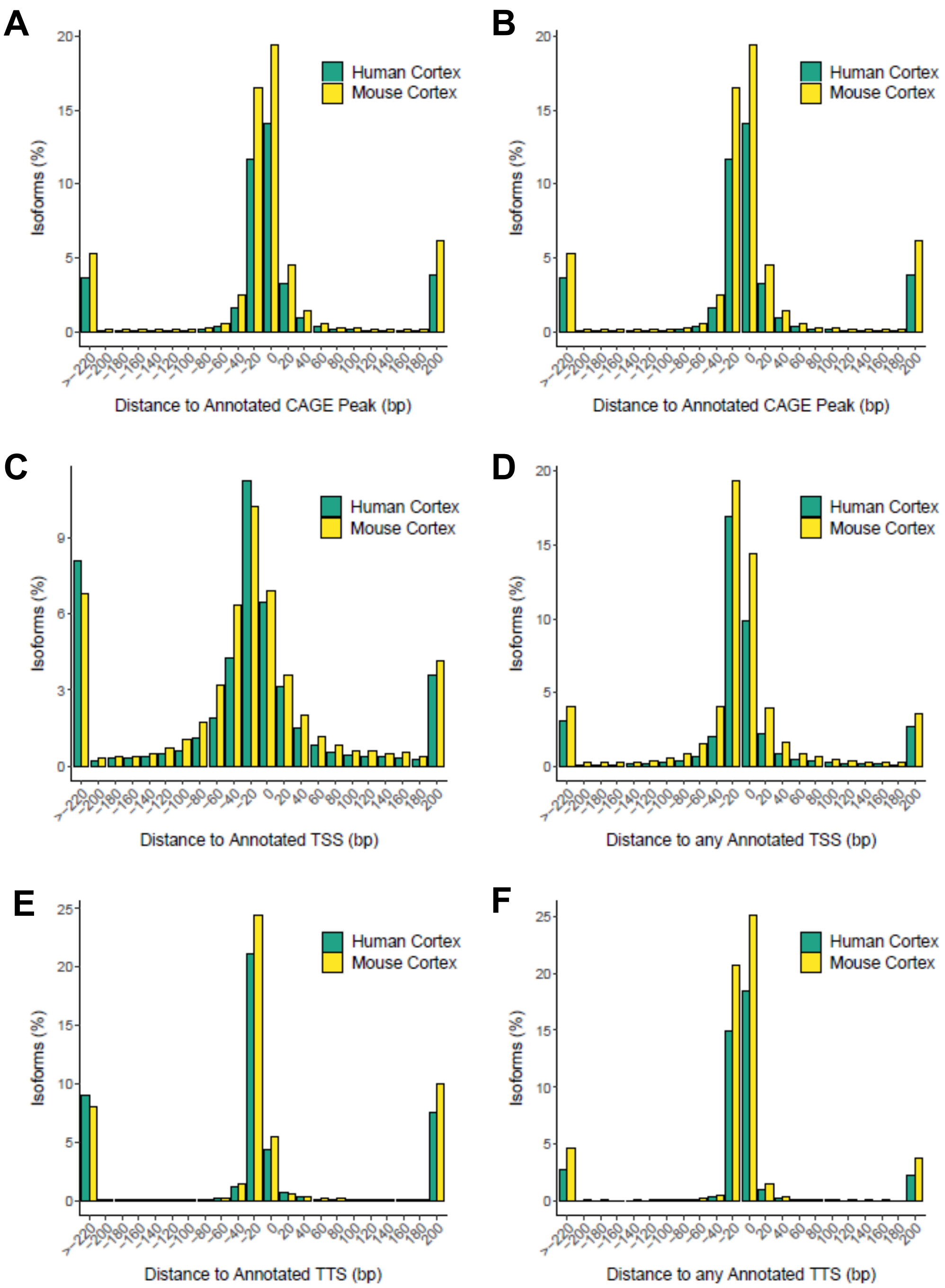

Figure S4: Transcripts identified by Iso-Seq are enriched near CAGE peaks, annotated transcription start sites (TSS) and transcript termination sites (TTS), related to Figure 1. Shown is the distance of transcripts to annotated CAGE peaks for **A)** all and **B)** novel transcripts. Shown also is the distance between the 5' end of each transcript to reference TSSs for **C)** all transcripts and **D)** novel transcripts. Finally, shown also is the distance between the 3' end of each transcript to reference TTSs for **E)** all transcripts and **F)** novel transcripts. A negative value for distance to TSS refers to a query start site downstream of reference, and a negative value for distance to TTS refers to end site upstream of reference. Novel transcripts are classified as NIC, NNC, antisense, genic/genomic, and fusion. TSS – Transcription Start Site, TTS – Transcription Termination Site.

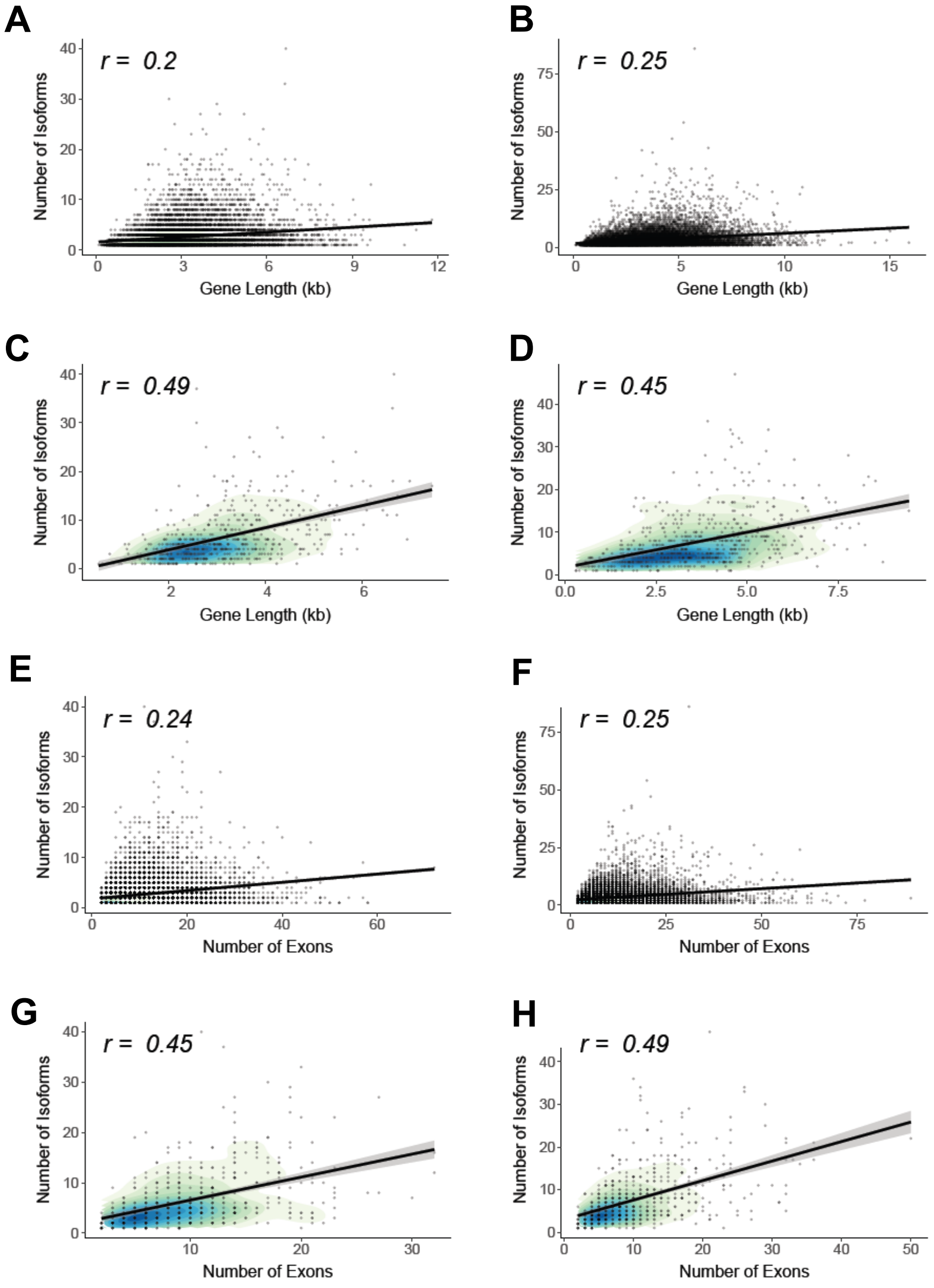

Figure S5: Longer genes and those with more exons tend to have a higher number of discrete isoforms, related to Figure 1. The number of detected multi-exonic isoforms in **A)** human cortex and **B)** mouse cortex is correlated with gene length (human cortex: Pearson's correlation = 0.19, P = 1.51 x 10^{-106}; mouse cortex: Pearson's correlation = 0.25, P = 1.33 x 10^{-197}). A stronger relationship was observed among 'highly-expressed' genes (>2.5 Log_{10} TPM) in both **C)** human cortex (Pearson's correlation = 0.49, P = 1.39 x 10^{-33}) and **D)** mouse cortex (Pearson's correlation = 0.45, P = 3.56 x 10^{-31}). The number of detected isoforms was also correlated with the number of exons in **E)** human cortex (Pearson's correlation = 0.24, P = 7.97 x 10^{-155}) and **F)** mouse cortex (Pearson's correlation = 0.24, P = 4.02 x 10^{-193}). A stronger relationship was observed among 'highly-expressed' genes (>2.5 Log_{10} TPM) in both **G)** human cortex (Pearson's correlation = 0.45, P = 7.42 x 10^{-28}) and **H)** mouse cortex (Pearson's correlation = 0.49, P = 2.16 x 10^{-38}). Gene length corresponds to the longest isoform, and density of genes is represented in increasing scale from light green to dark blue. TPM – Transcripts per Million.

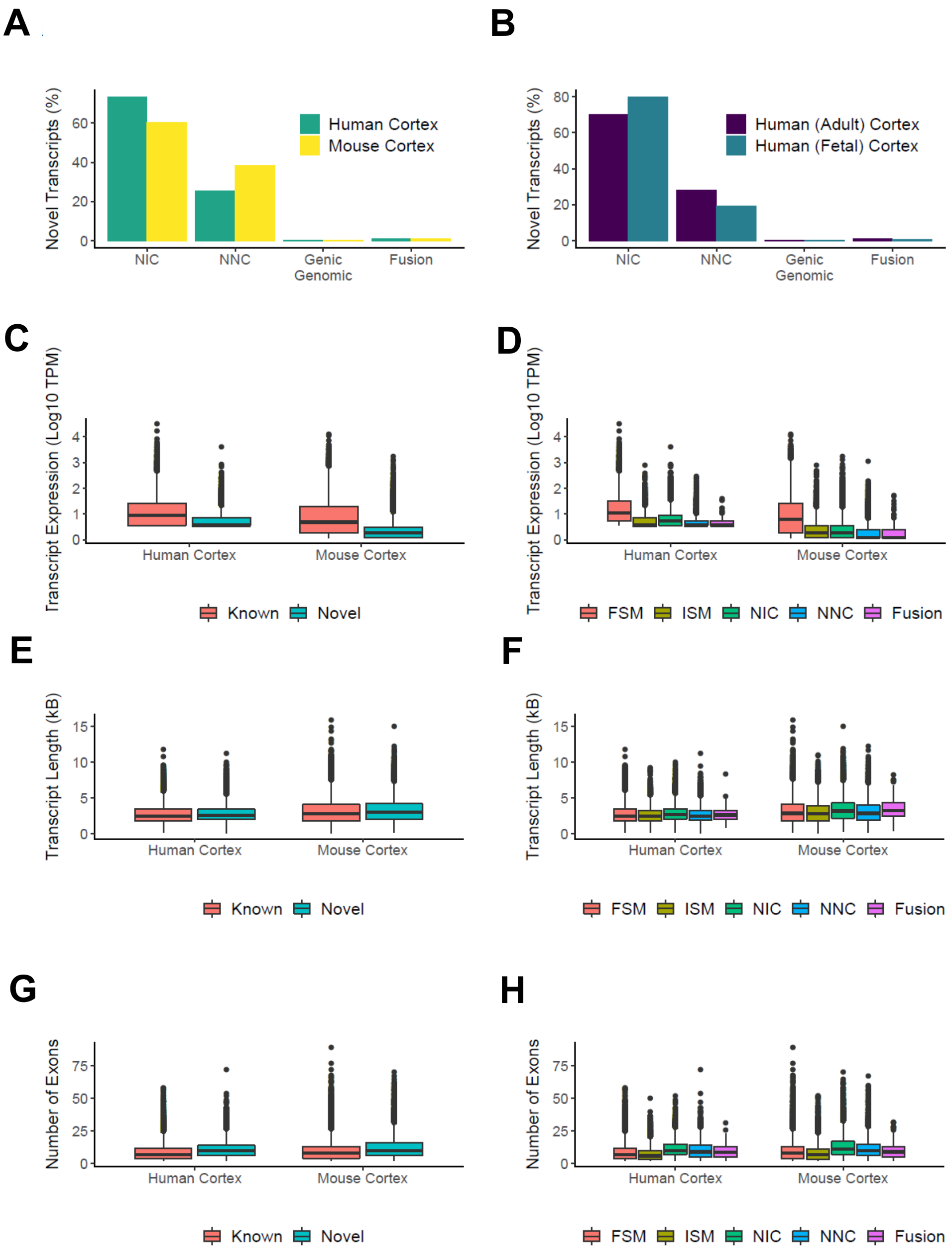

Figure S6: Differences in the abundance and size of novel and known transcripts, related to Figure 2. Shown is the proportion of novel transcripts comparing **A)** human cortex with mouse cortex and **B)** human adult cortex with human fetal cortex. Definitions of the different isoform classifications are shown in **Figure 2A**. **C)** Overall Iso-Seq transcript expression of novel and known transcripts and **D)** different RNA isoform categories. Known transcripts were more highly expressed than novel transcripts in both human (Mann-Whitney-Wilcoxon test, $W = 1.62x\ 10^{8}$, $P < 2.23 \times 10^{-308}$) and mouse cortex (Mann-Whitney-Wilcoxon test, $W = 3.66 \times 10^{8}$, $P < 2.23 \times 10^{-308}$). **E, F)** Transcript length and **G, H)** number of exons for novel and known transcripts of annotated genes, further stratified by RNA isoform category. Novel transcripts were longer (human cortex: Mann-Whitney-Wilcoxon test, $W = 1.10 \times 10^{8}$, $P = 4.04 \times 10^{-25}$; mouse cortex: Mann-Whitney-Wilcoxon test $W = 2.37x\ 10^{8}$, $P = 2.13 \times 10^{-42}$) and had more exons (human cortex: $W = 8.83 \times 10^{7}$, $P < 2.23 \times 10^{-308}$; mouse cortex: $W = 1.94 \times 10^{8}$, $P < 2.23 \times 10^{-308}$) than known transcripts.

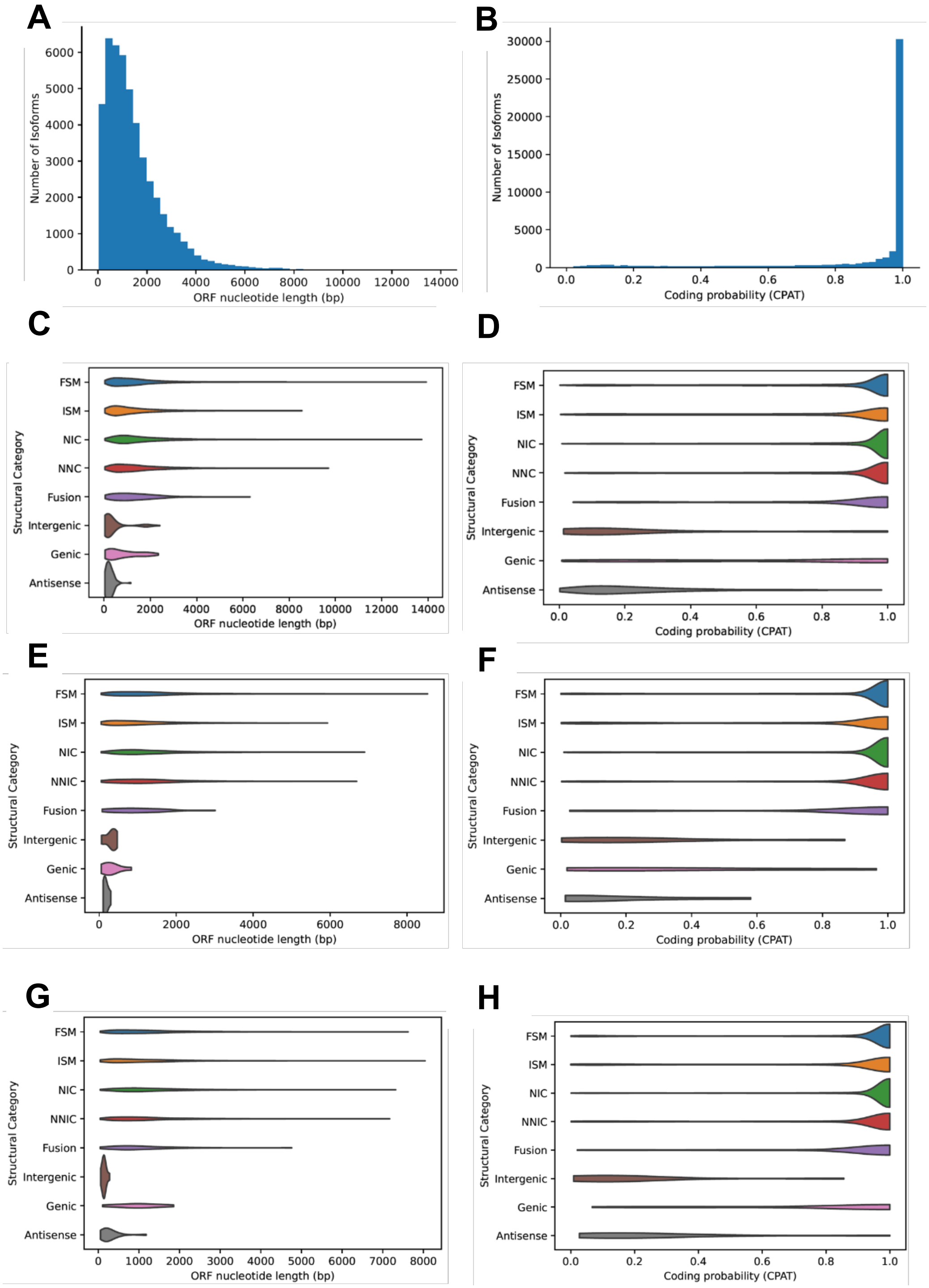

Figure S7: Characterizing open reading frames highlighted a high level of coding potential amongst identified transcripts, related to Figures 1 and 2. Shown is **A)** the distribution of ORF nucleotide length and **B)** CPAT coding probability for all isoforms in mouse cortex (n = 12 biologically independent samples). Also shown is **C)** the distribution of ORF nucleotide length and **D)** CPAT coding probability in mouse cortex stratified by transcript category. The equivalent plots of the distribution of ORF nucleotide length and coding probability are shown for human fetal cortex (**E, F**), human adult cortex (**G, H**) and for human cortex (see **Figure 1C, Figure 1D, Figure 2C, and Figure 2D**).

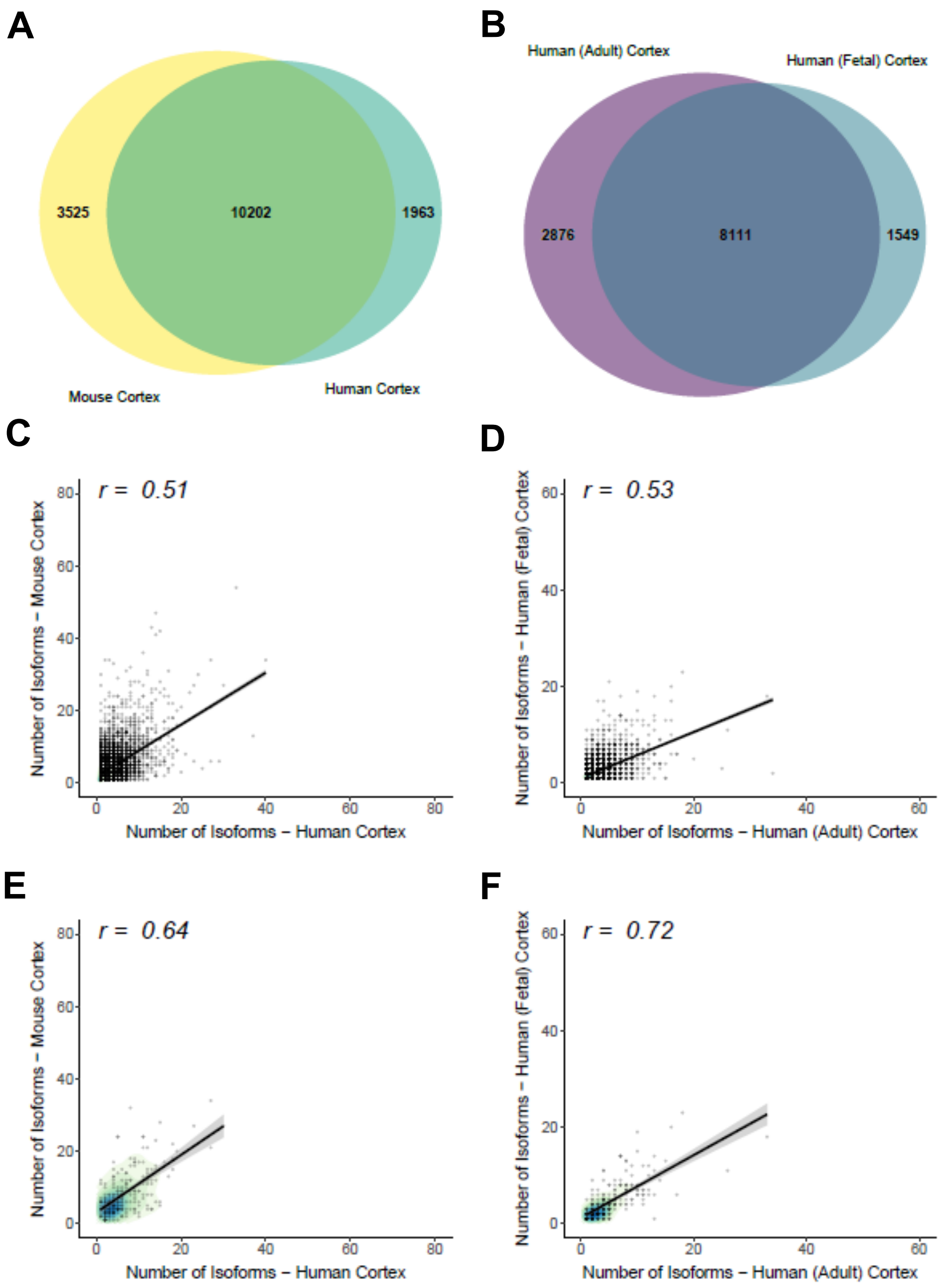

Figure S8: The number of unique isoforms for commonly expressed genes in human and mouse cortex is correlated between species, related to Figure 3. Shown is the number of common and unique annotated genes from GENCODE (human: hg38, mouse: mm10) identified in **A)** a comparison of human and mouse cortex and **B)** a comparison of human adult and human fetal cortex. Of the genes commonly identified, a strong relationship was observed between the number of multi-exonic isoforms in human and mouse cortex across **C)** all transcripts and **D**) in highly-expressed genes (>2.5 Log_{10} TPM). Similarly, a strong relationship was observed between the number of multi-exonic isoforms in human adult and human fetal cortex across **E)** all transcripts and **F)** in highly-expressed genes (>2.5 Log_{10} TPM).

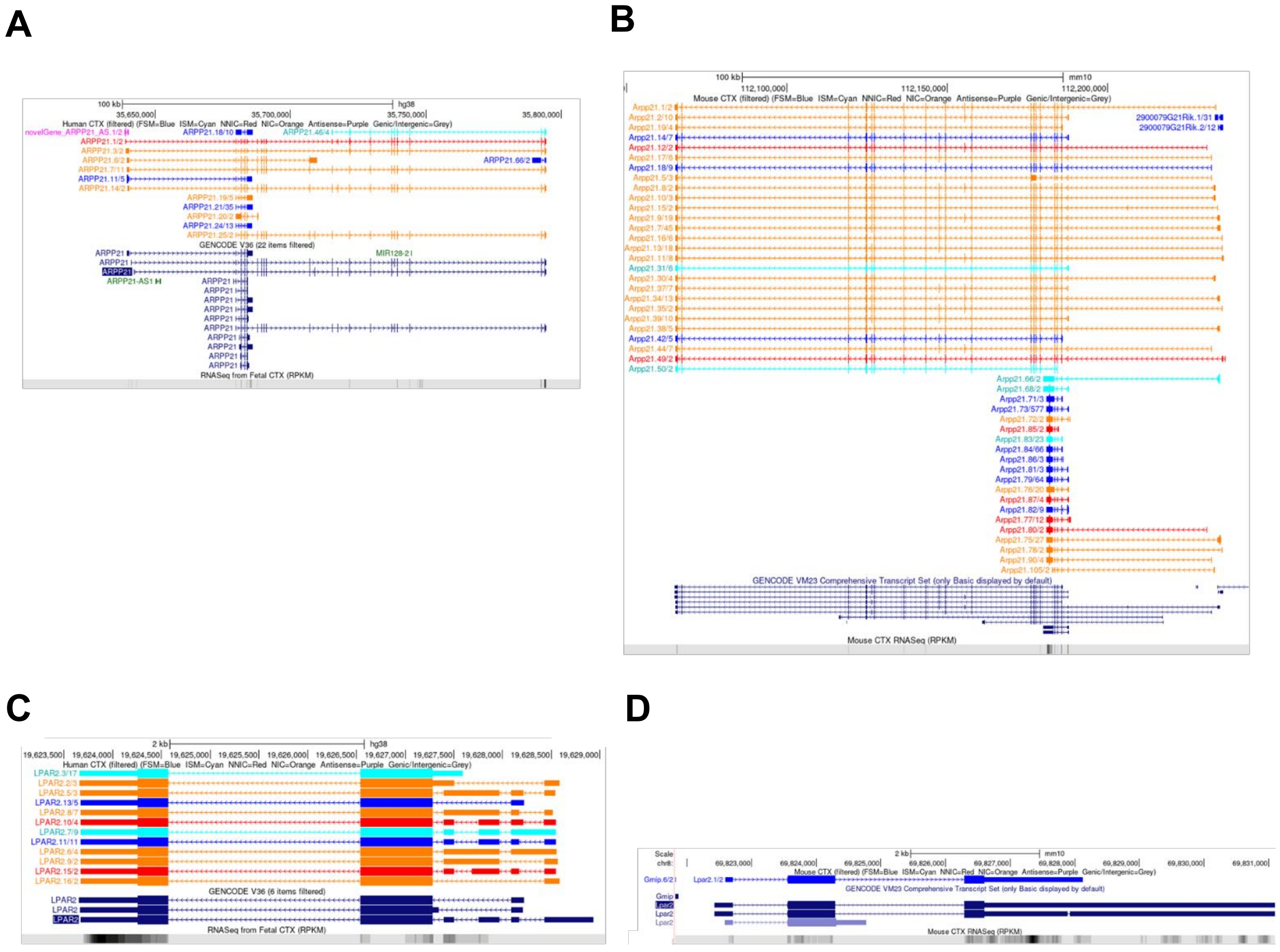

Figure S9: Notable difference in isoform number in *ARPP21* /*Arpp21* and *LPAR2* /*Lpar2* gene between the human and mouse cortex, related to Figure 3. Shown is a UCSC genome browser track of **A)** the *ARPP21* gene in human cortex (n = 14 multi-exonic isoforms) and **B)** the *Arpp21* gene in mouse cortex (n = 47 multi-exonic isoform). Also shown is a UCSC genome browser track of **C)** the *LPAR2* gene in human cortex (n = 12 multi-exonic isoforms) and **D)** the *Lpar2* gene in mouse cortex (n = 1 multi-exonic isoform). Isoforms are coloured based on *SQANTI2* classification categories (blue = FSM, cyan = ISM, red = NNC, orange = NIC). FSM – Full Splice Match, ISM – Incomplete Splice Match, NIC – Novel In Catalogue, NNC – Novel Not in Catalogue

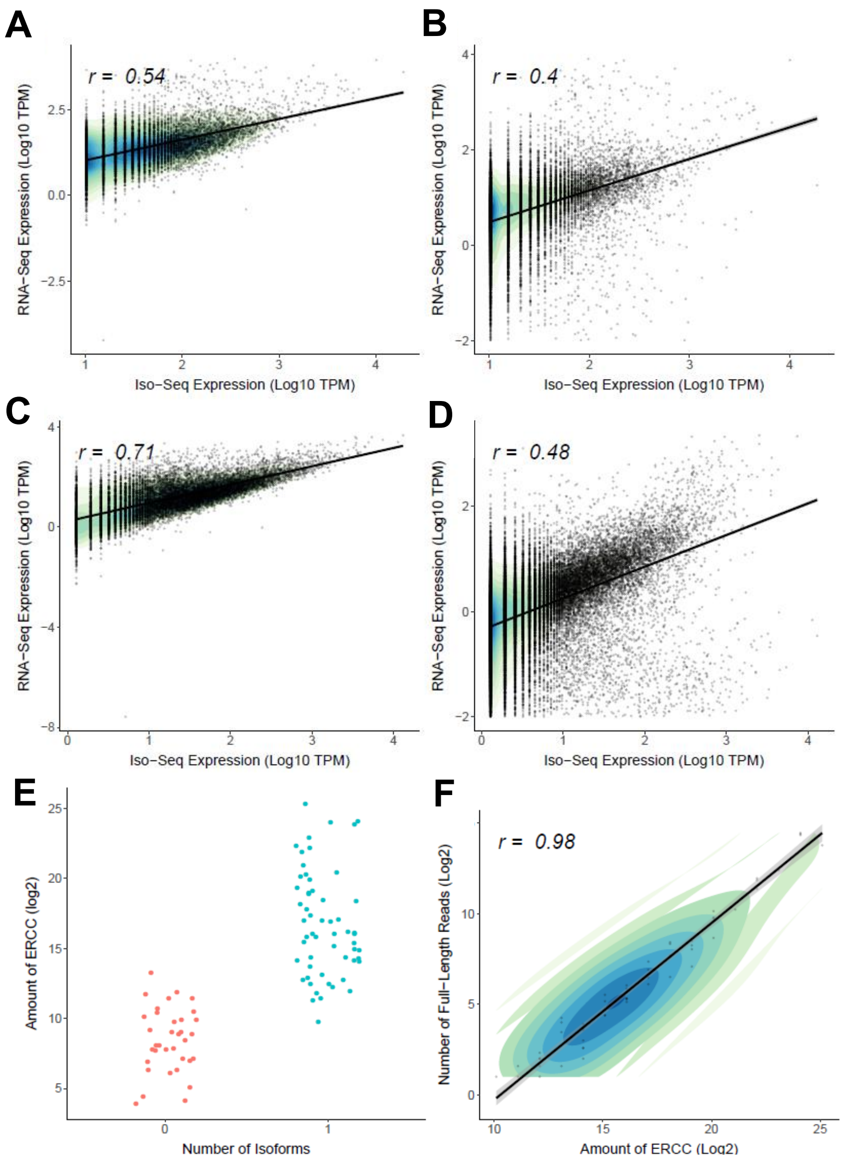

Figure S10: Long-read Iso-Seq data can be used to accurately quantify levels of gene expression in the human cortex, related to STAR Methods. Shown is the relationship between expression estimated using RNA-Seq and Iso-Seq at the **A)** gene level (n = 9,223 genes, Pearson's correlation = 0.58, $P < 2.23 \times 10^{-308}$) and **B)** transcript level (n = 17,583 transcripts, corr = 0.40, $P < 2.23 \times 10^{-308}$) in the human cortex (data derived from three biologically independent fetal samples). Also shown is the same relationship at the **C)** gene level (n = 13,923 genes, corr = 0.71; $P < 2.23 \times 10^{-308}$) and **D)** transcript level (n = 41,488 transcripts, corr = 0.48, $P < 2.23 \times 10^{-308}$) for mouse cortex (n = 12 biologically independent samples). RNA-Seq gene expression was determined after aligning short-read RNA-Seq to the Iso-Seq transcriptome. Iso-Seq gene expression was determined from the sum of full-length, multi-exonic transcript reads associated for each gene, with TPM values calculated by dividing the number of full length reads per gene by total full-length reads, multiplied by a million. The density of values is represented in increasing scale from light green to dark blue. **E)** The number of ERCC spike-in fragments detected compared to the amount used in our mouse cortex Iso-Seq libraries and **F)** the relationship between the amount of ERCC used and the number of full-length reads identified (Pearson's correlation = 0.98, $P = 1.42 \times 10^{-41}$). There is a near perfect correlation between full-length reads associated with ERCC spike-in fragments and the actual amount of control used.

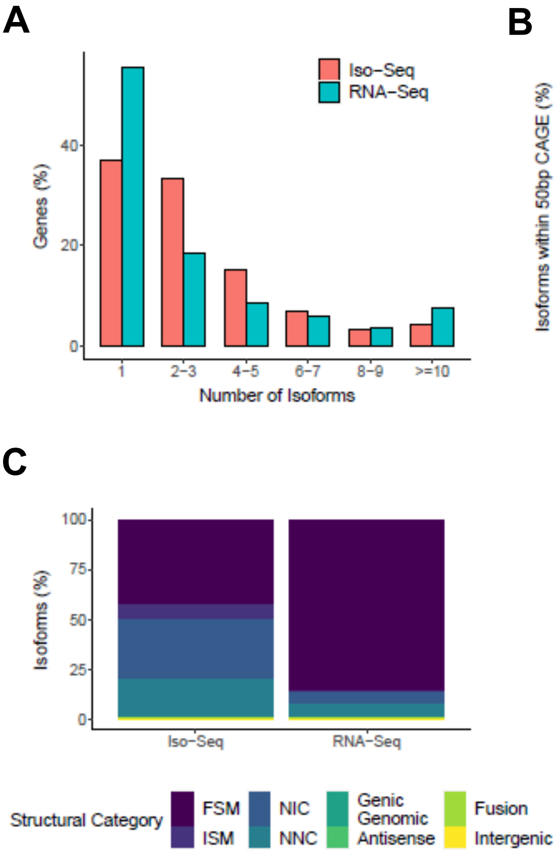

Figure S11: Transcripts from an RNA-Seq defined transcriptome were less supported by CAGE peaks, and likely represent incomplete fragments of transcripts identified using Iso-Seq reads, related to STAR Methods. Shown is a comparison of the **A)** distribution of number of isoforms associated per gene, **B)** the proportion of isoforms annotated within 50bp of a CAGE peak, and **C)** classification of the isoforms using *SQANTI* categories, between Iso-Seq defined and RNA-Seq defined transcriptomes generated on the mouse cortex. RNA-Seq defined transcriptome is generated using a reference-guided assembly of RNA-Seq reads (n = 12 biologically independent samples) using *Stringtie.*

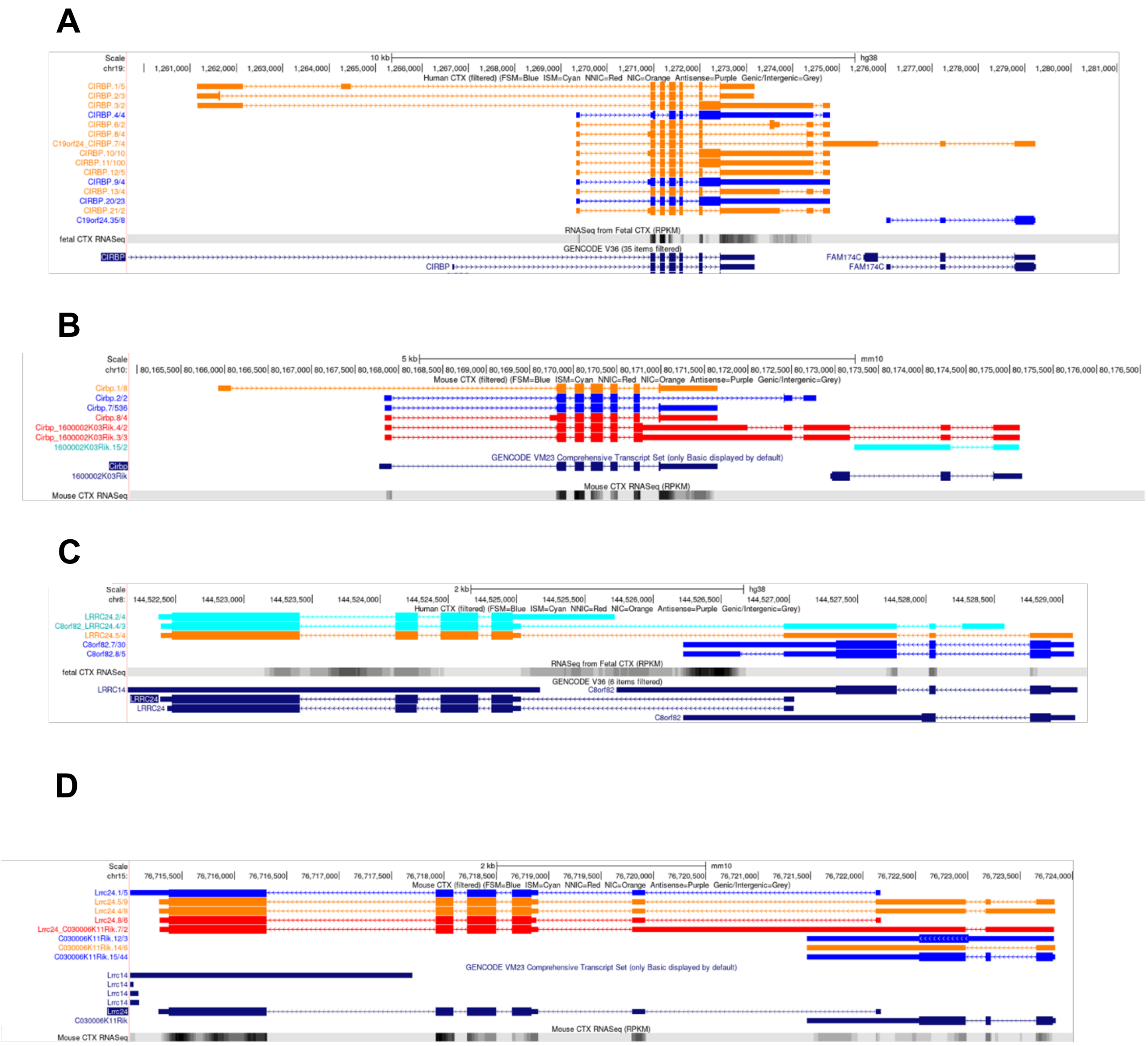

Figure S12: Additional examples of similar fusion transcripts observed in both human and mouse cortex, related to Figure 4. Shown are UCSC tracks of **A)** the *CIRBP-FAM174C* gene in the human cortex and **B)** the *Cirbp-1600002K03RIK* gene in the mouse cortex. Also shown are UCSC tracks of **C)** the *C8orf82-LRCC24* gene in the human cortex and **D)** the *Lrcc24-C030006K11Rik* gene in the mouse cortex. Isoforms are coloured based on *SQANTI2* classification categories (blue = FSM, cyan = ISM, red = NNC, orange = NIC). FSM – Full Splice Match, ISM – Incomplete Splice Match, NIC – Novel In Catalogue, NNC – Novel Not in Catalogue

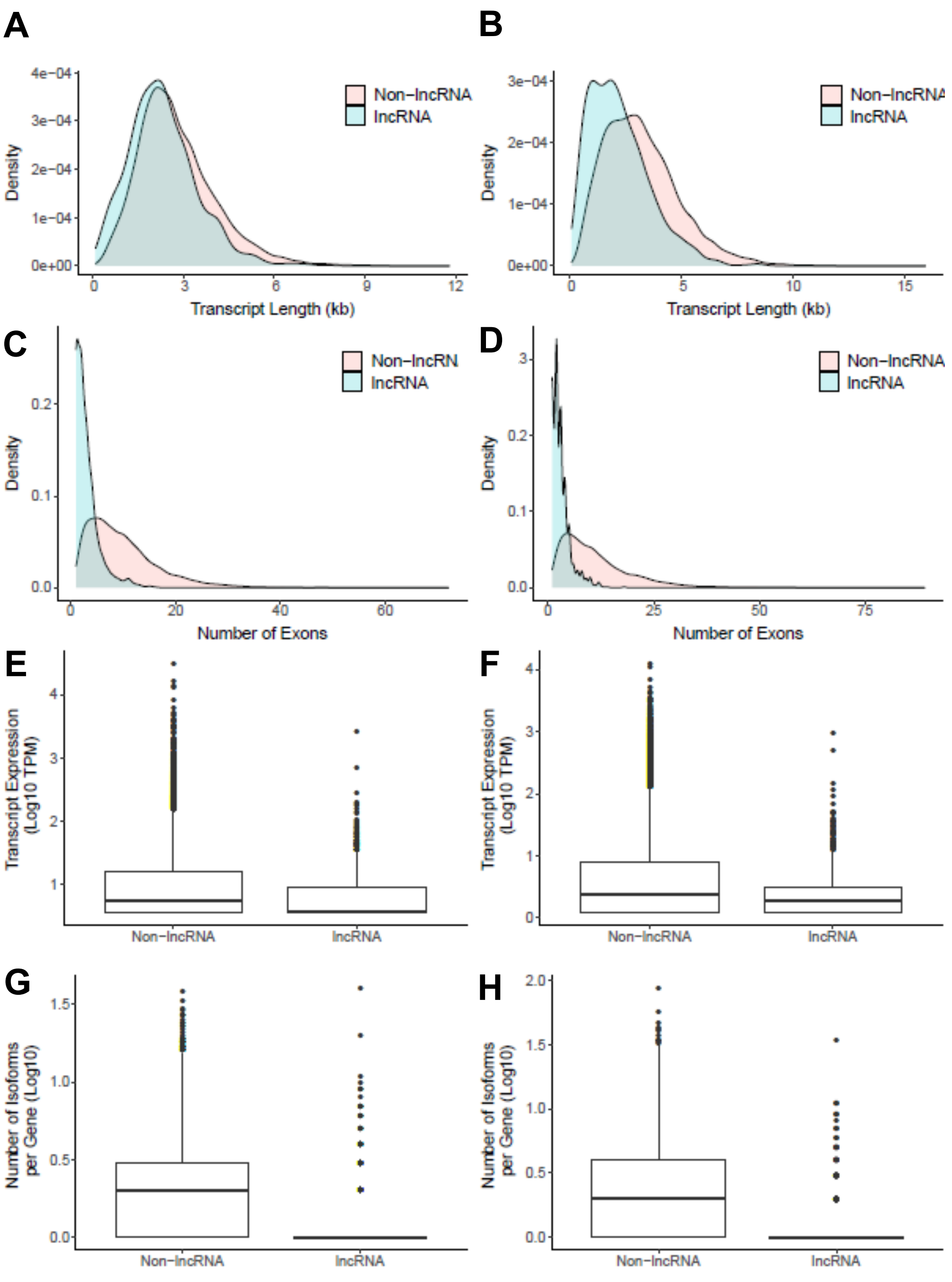

Figure S13: lncRNA transcripts are typically shorter, contain fewer exons, are less abundant and have fewer isoforms than non-lncRNA transcripts, related to Table 1. LncRNA transcripts were found to be shorter in both **A)** human cortex (Mann-Whitney-Wilcoxon, W = 2.28×10^{7}, P = 3.22×10^{-34}) and **B)** mouse cortex (Mann-Whitney-Wilcoxon test, 3.52×10^{7}, P = 8.24×10^{-98}). They also contained fewer exons in both **C)** human cortex (Mann-Whitney-Wilcoxon test, W = 3.31×10^{7}, P < 2.23×10^{-308}) and **D)** mouse cortex (Mann-Whitney-Wilcoxon test, W = W = 4.56×10^{7}, P < 2.23×10^{-308}). They were also characterized by lower transcript expression than non-lncRNA transcripts in **E)** human (Mann-Whitney-Wilcoxon test, W = 2.27×10^{7}, P = 9.44×10^{-35}) and **F)** mouse cortex (Mann-Whitney-Wilcoxon test, W = 3.16×10^{7}, P = 5.67×10^{-40}). Finally, they showed lower isoform diversity in **G)** human (Mann-Whitney-Wilcoxon test, W = 6.63×10^{6}, P = 1.21×10^{-80}) and **H)** mouse cortex (Mann-Whitney-Wilcoxon test, W = 7.40×10^{6}, P = 5.76×10^{-107}). Lnc-RNA – long non-coding RNA.

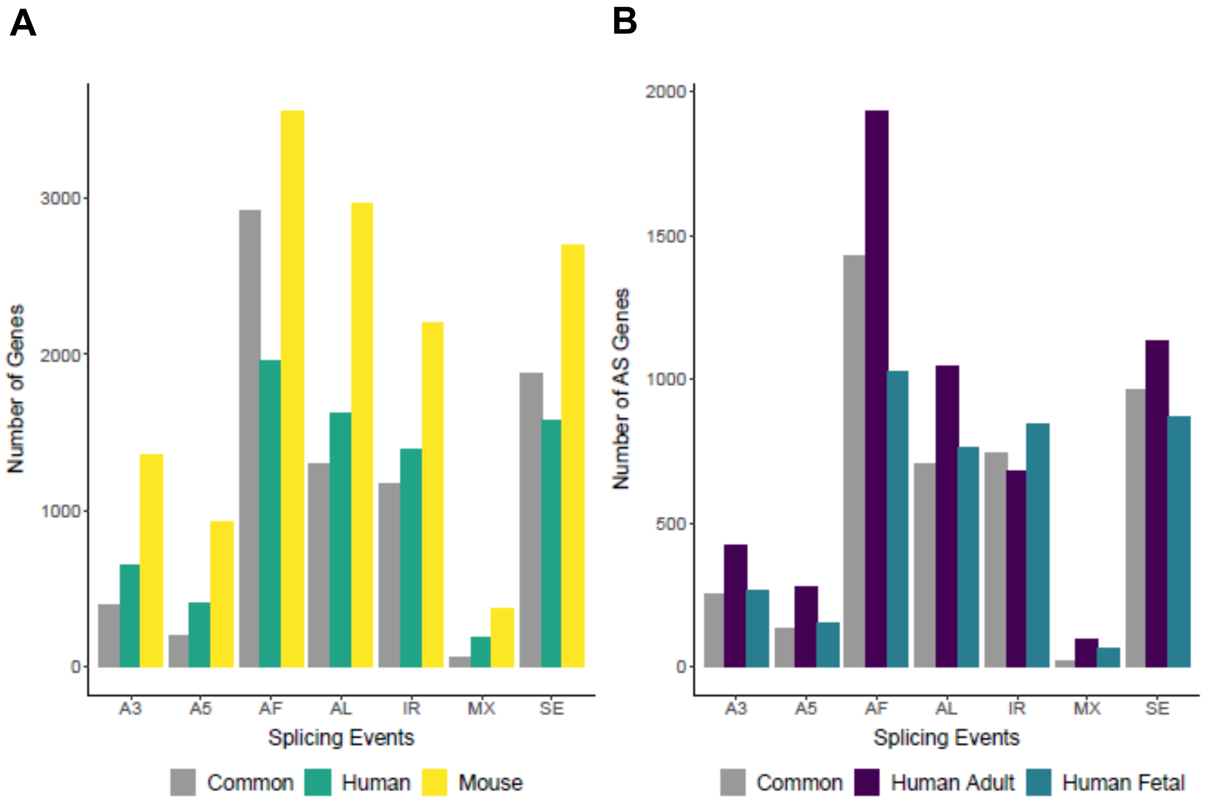

Figure S14: Alternative first exon and alternative last exon are the most prominent AS events in human and mouse cortex, related to Figure 5. There was also a large overlap of genes influenced by specific AS events in human and mouse cortex, as shown by number of genes commonly observed with the different AS events (A3, A5, AF, AL, IR, MX, and SE) between **A)** human and mouse cortex, and between **B)** human adult and human fetal cortex. MX and SE events were determined using *SUPPA2*, IR with *SQANTI2* and A3', A5', AF and AL with custom scripts. AF – Alternative First Exon, AL – Alternative Last Exon, A5' – Alternative 5' prime, A3' – Alternative 3' prime, IR – Intron Retention, MX – Mutually Exclusive, SE – Skipped Exon. Of note, genes observed with individual AS events are not mutually exclusive.

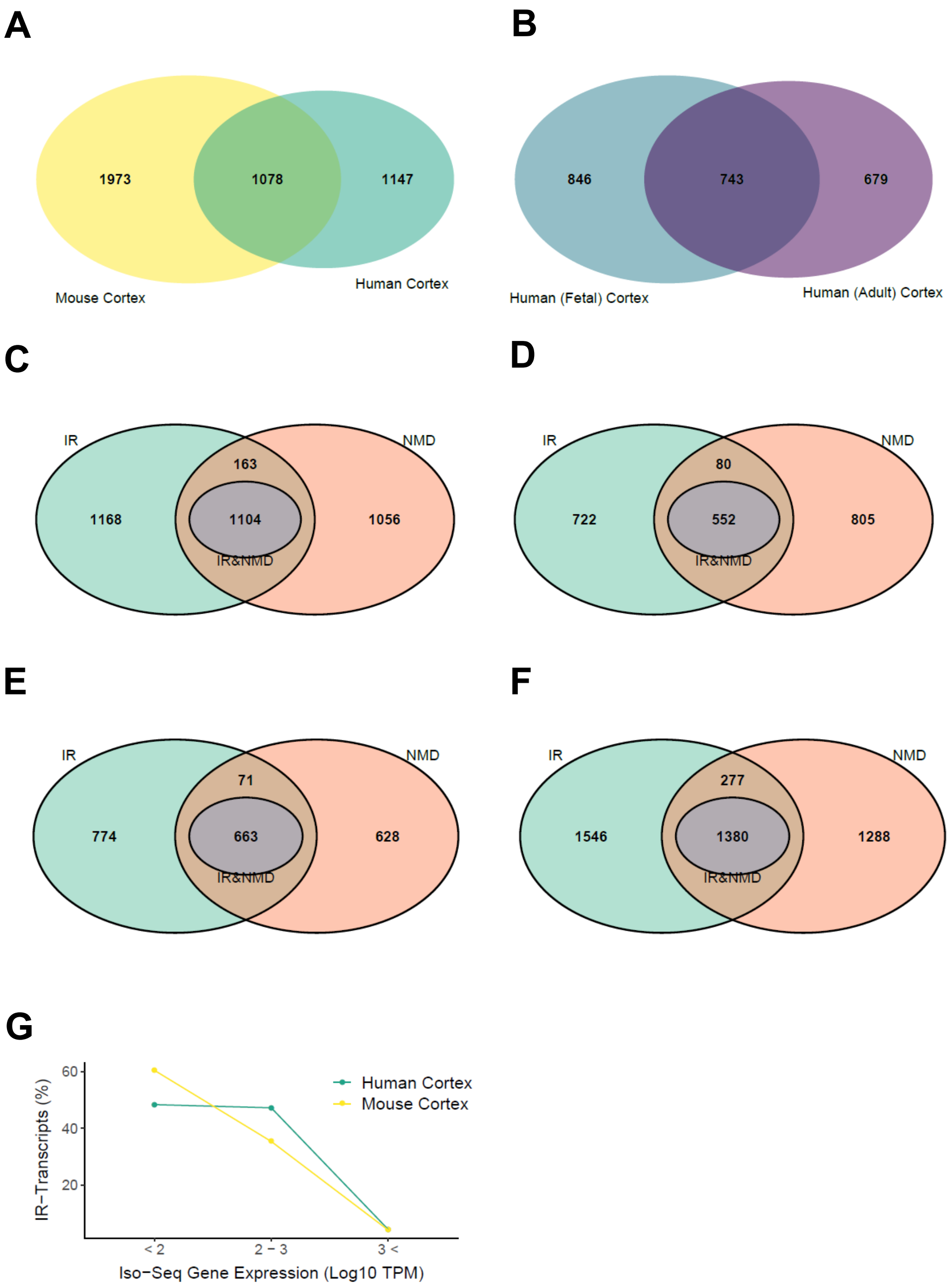

Figure S15: Intron retention and NMD in the human and mouse cortex, related to Figure 5. Shown is the number of genes with intron-retained transcripts comparing A**)** human and mouse cortex and **B)** human adult and human fetal cortex. NMD is particularly enriched amongst transcripts with intron retention**,** as shown by the overlap of genes with IR-transcripts, NMD- transcripts, and transcripts with both IR and NMD in **C)** human cortex, **D)** human adult cortex, **E)** human fetal cortex and **F)** mouse cortex. Genes containing both IR and NMD transcripts were further classified into genes that contain transcripts that were *both* IR and NMD (purple) and genes that contain transcripts where IR and NMD were mutually exclusive (dark orange). **G)** A larger proportion of lowly expressed genes showed evidence for IR than highly expressed genes in both human (< 2.5 Log_{10} TPM, n = 2,269 (88.4%) genes; > 2.5 Log_{10} TPM, n = 297 (11.6%) genes) and mouse (< 2.5 Log_{10} TPM, n = 3,039 (90.04%) genes; > 2.5 Log_{10} TPM, n = 336 (9.96%) genes. IR – Intron retention. NMD – Nonsense-mediated mRNA decay. TPM – Transcripts per Million

A

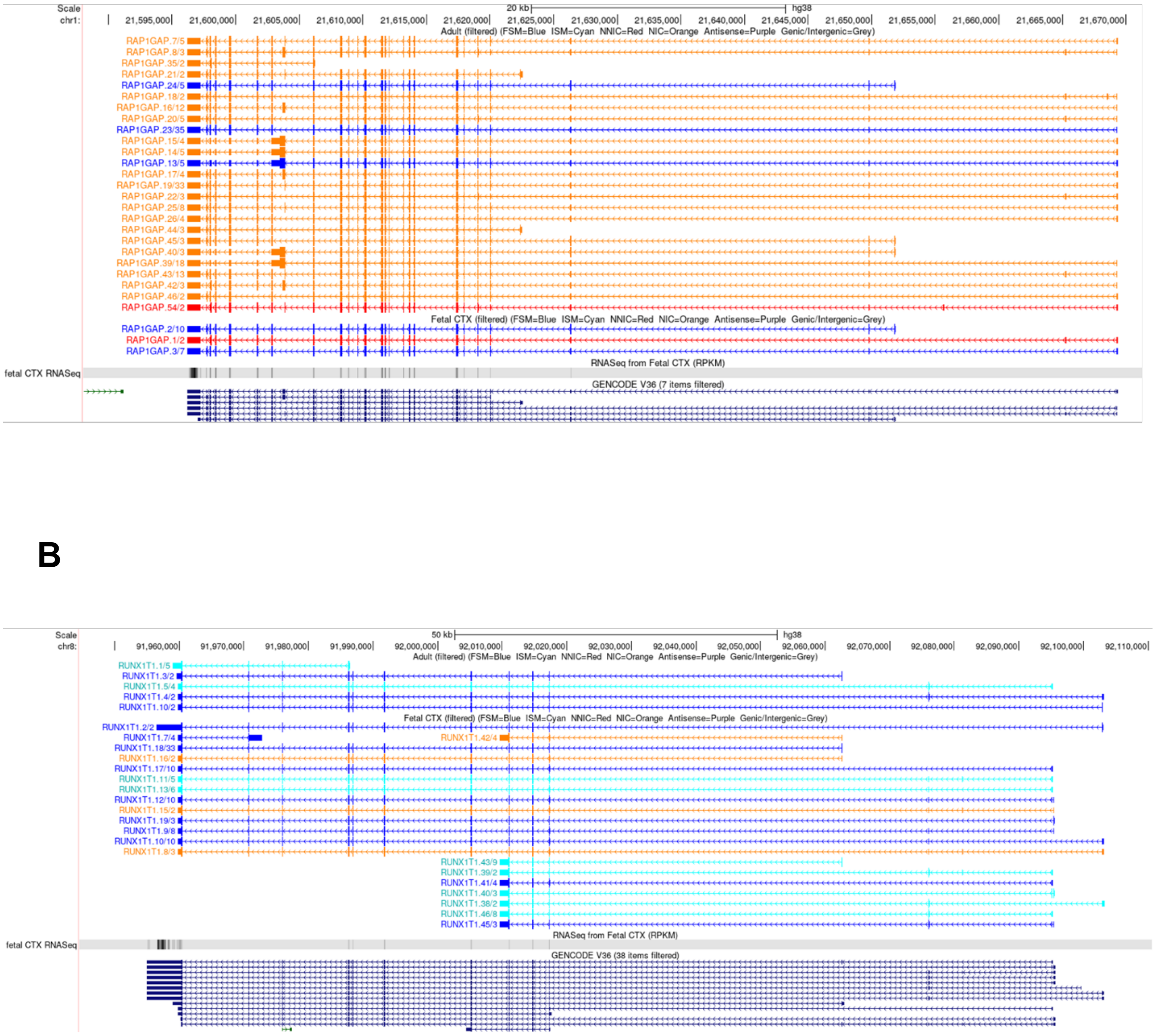

B

Figure S16: Notable differences in isoform number among transcripts of *RAP1GAP* and *RUNX1T1* between human adult and fetal cortex, related to Figure 3. Shown is a UCSC genome browser track of **A)** *RAP1GAP* and **B)** *RUNX1T1* in human adult and human fetal cortex. These genes are characterized by a large absolute difference in isoform numbers detected between human fetal and human adult cortex: *RAP1GAP* (adult cortex: n = 25 isoforms; fetal cortex, n = 3 isoforms) and *RUNX1T1* (adult cortex: n = 5 isoforms; fetal cortex: n = 21 isoforms). Isoforms are coloured based on *SQANTI2* classification categories (blue = FSM, cyan = ISM, red = NNC, orange = NIC). FSM – Full Splice Match, ISM – Incomplete Splice Match, NIC – Novel In Catalogue, NNC – Novel Not in Catalogue

A

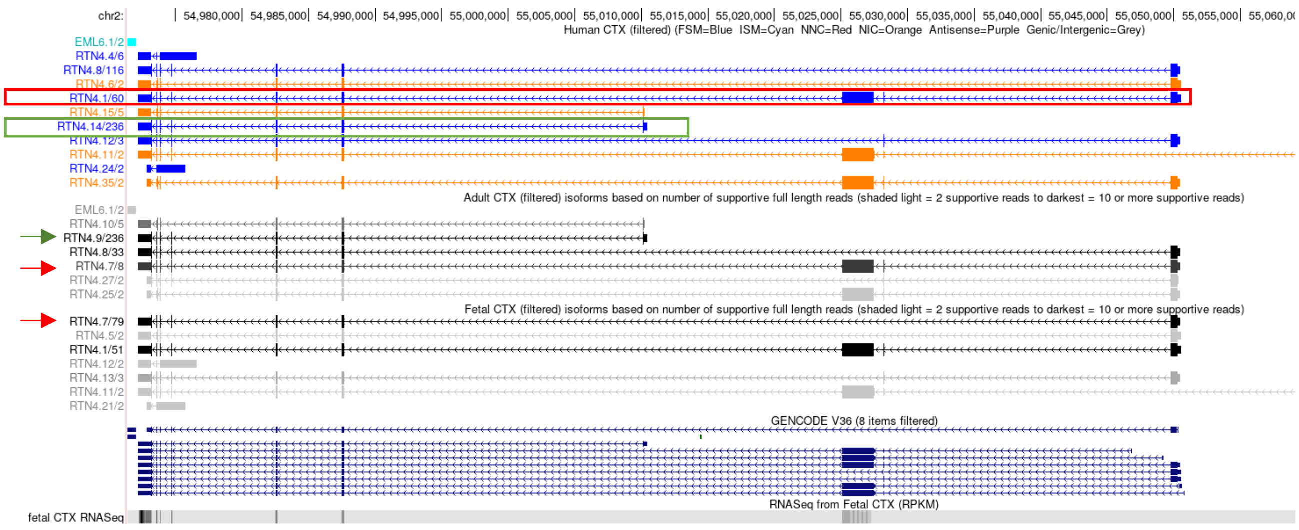

B

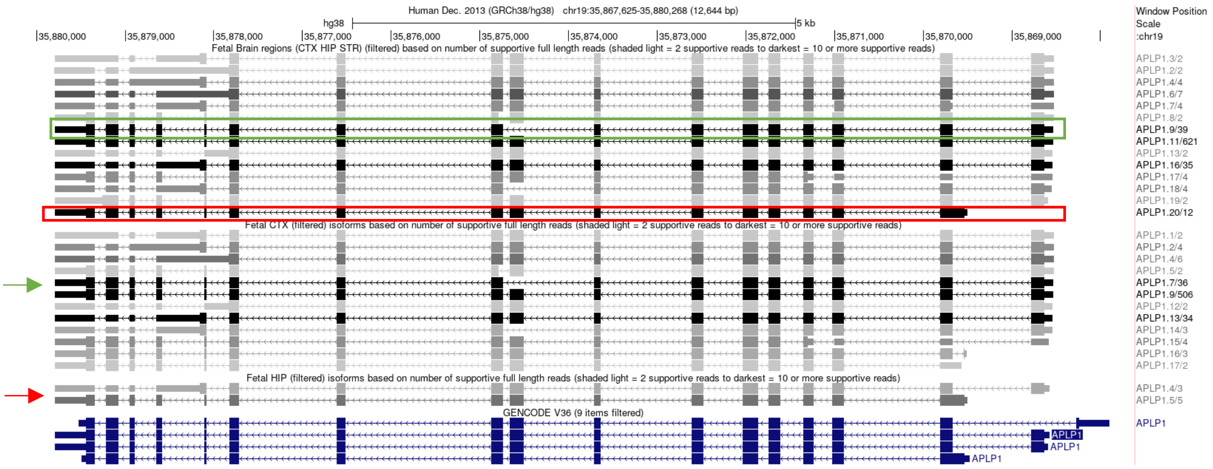

Figure S17: Differential transcript usage between human adult and fetal cortex for *RTN4* and *APLP1,* related to Figures 3 and 5. Shown are UCSC genome browser tracks for **A)** *RTN4* transcripts in human cortex, coloured by transcript classification, and **B)** *APLP1* transcripts in human fetal brain regions, shaded by the number of full-length reads. Differential transcript usage was observed in *RTN4*, with one isoform (boxed in red – RTN4.1, red arrow) strongly expressed in fetal cortex while downregulated in adult cortex, and another isoform (boxed in green – RTN4.14, green arrow) strongly expressed only in adult cortex. Differential transcript usage was observed in *APLP1* with one transcript (boxed in red – APLP1.20, ENST00000586861.5) strongly expressed in fetal hippocampus (red arrow) while not detected in fetal cortex, and another novel isoform (boxed in green – APLP1.9) strongly expressed in fetal cortex (green arrow).

Sample ID	Number of SMRT Cells	Species	Tissue	Sex	Age	RIN	RNA-Seq (Illumina)	Nanopore sequencing (ONT)
Adult A	1	Human	Cortex	F	89years	4.1	No	No
Adult B	1	Human	Cortex	F	89years	6.3	No	No
Adult C	2	Human	Cortex	M	24years	8	No	Yes
Adult D	1	Human	Cortex	M	45years	7	No	No
Fetal A	1	Human	Cortex	M	17wpc	8.3	Yes	Yes
Fetal B	2	Human	Cortex	F	17wpc	7.2	Yes	No
Fetal C	2	Human	Cortex	F	14wpc	6.1	Yes	No
Fetal D	1	Human	Hippocampus	F	17wpc	7.5	No	No
Fetal E	1	Human	Hippocampus	F	14wpc	7.1	No	No
Fetal F	1	Human	Striatum	F	17wpc	8.1	No	No
Fetal G	1	Human	Striatum	F	14wpc	6.9	No	No
Mouse A	1	Mouse	Cortex	F	2months	9.2	Yes	No
Mouse B	1	Mouse	Cortex	F	2months	8.8	Yes	No
Mouse C	1	Mouse	Cortex	F	8months	9.1	Yes	No
Mouse D	1	Mouse	Cortex	F	8months	9.2	Yes	No
Mouse E	1	Mouse	Cortex	F	8months	8.7	Yes	No
Mouse F	1	Mouse	Cortex	F	2months	9.2	Yes	No
Mouse G	1	Mouse	Cortex	F	2months	8.9	Yes	No
Mouse H	1	Mouse	Cortex	F	8months	9	Yes	No
Mouse I	1	Mouse	Cortex	F	8months	8.6	Yes	No
Mouse J	1	Mouse	Cortex	F	2months	9.2	Yes	No
Mouse K	1	Mouse	Cortex	F	2months	8.9	Yes	No
Mouse L	1	Mouse	Cortex	F	8months	9.1	Yes	No

Table S1: Description of the individual samples included in this study, related to STAR Methods. Of note fetal hippocampus and striatum samples were derived from the same donor as the fetal cortex samples. SMRT – Single-molecule real-time, ONT – Oxford Nanopore Technology, RIN – RNA Integrity Number, wpc – weeks post-conception

Run ID	Sample ID	Species	Tissue	Number of CCS generated
Adult 1	Adult A	Human	Cortex	388960
Adult 2	Adult B	Human	Cortex	420057
Adult 3	Adult C	Human	Cortex	352900
Adult 4	Adult C	Human	Cortex	351182
Adult 5	Adult D	Human	Cortex	356361
Fetal 1	Fetal A	Human	Cortex	196123
Fetal 2	Fetal B	Human	Cortex	69247
Fetal 3	Fetal B	Human	Cortex	471115
Fetal 4	Fetal C	Human	Cortex	199170
Fetal 5	Fetal C	Human	Cortex	493077
Fetal 6	Fetal D	Human	Hippocampus	214602
Fetal 7	Fetal E	Human	Hippocampus	206790
Fetal 8	Fetal F	Human	Striatum	268602
Fetal 9	Fetal G	Human	Striatum	202211
Mouse 1	Mouse A	Mouse	Cortex	470079
Mouse 2	Mouse B	Mouse	Cortex	464504
Mouse 3	Mouse C	Mouse	Cortex	503595
Mouse 4	Mouse D	Mouse	Cortex	509175
Mouse 5	Mouse E	Mouse	Cortex	511698
Mouse 6	Mouse F	Mouse	Cortex	461026
Mouse 7	Mouse G	Mouse	Cortex	353481
Mouse 8	Mouse H	Mouse	Cortex	493532
Mouse 9	Mouse I	Mouse	Cortex	498732
Mouse 10	Mouse J	Mouse	Cortex	409403
Mouse 11	Mouse K	Mouse	Cortex	486693
Mouse 12	Mouse L	Mouse	Cortex	495762

Table S3: Number of successful CCS reads generated across samples included in this study, related to Figure 1. Distribution of CCS read lengths across all cortical samples can be found in **Figures S3A-S3D.** CCS - Circular consensus sequence

		Human Cortex	Human Cortex		Mouse Cortex
			Adult	Fetal	
	Unique Genes	12964	11021	9679	14684
	Annotated Genes	12910 (99.58%)	10987 (99.69%)	9660 (99.8%)	14482 (98.62%)
	Novel Genes	54 (0.42%)	34 (0.31%)	19 (0.2%)	202 (1.38%)
Annotated Genes	Transcripts	32745	22013	18592	46403
	Protein-coding Transcripts	30388(92.8%)	20525(93.24%)	17456(93.89%)	43450(93.64%)
	Genes associated with coding transcripts	12087	10315	9198	13710
	Non-lncRNA Transcripts	31548	21293	18054	45262
	lncRNA Transcripts	1197	720	538	1141
	Mono-exonic non lncRNA	583(1.85%)	440(2.07%)	365(2.02%)	914(2.02%)
	Mono-exonic lncRNA Transcripts	348(29.07%)	232(32.22%)	174(32.34%)	273(23.93%)
	Genes associated with lncRNA Transcripts	792	535	365	734
	Protein-coding lncRNA Transcripts	521(43.53%)	310(43.06%)	233(43.31%)	448(39.26%)
	Annotated Transcripts (FSM, ISM)	20832 (63.62%)	15659 (71.14%)	13177 (70.87%)	23530 (50.71%)
	Novel Transcripts	11913 (36.38%)	6354 (28.86%)	5415 (29.13%)	22873 (49.29%)
	FSM	17080 (52.16%)	13007 (59.09%)	11346 (61.03%)	19803 (42.68%)
	ISM	3752 (11.46%)	2652 (12.05%)	1831 (9.85%)	3727 (8.03%)
	NIC	8721 (26.63%)	4464 (20.28%)	4315 (23.21%)	13763 (29.66%)
	NNC	3021 (9.23%)	1796 (8.16%)	1041 (5.6%)	8751 (18.86%)
	Genic Genomic	35 (0.11%)	20 (0.09%)	8 (0.04%)	62 (0.13%)
	Antisense	0 (0%)	0 (0%)	0 (0%)	0 (0%)
	Fusion	136 (0.42%)	74 (0.34%)	51 (0.27%)	297 (0.64%)
	Intergenic	0 (0%)	0 (0%)	0 (0%)	0 (0%)
	Genic Intron	0 (0%)	0 (0%)	0 (0%)	0 (0%)
	Genes associated with Novel Transcripts	5327 (41.26%)	3468 (31.56%)	3027 (31.34%)	8054 (55.61%)
	Genes associated with Annotated Transcripts	12137 (94.01%)	10350 (94.2%)	9079 (93.99%)	13551 (93.57%)
	NMD Transcripts	4370 (13.35%)	2337 (10.62%)	2222 (11.95%)	6014 (12.96%)
	Genes with NMD transcripts	2323 (17.99%)	1437 (13.08%)	1362 (14.1%)	2945 (20.34%)

Fusion Genes	108 (0.84%)	45 (0.41%)	58 (0.6%)	218 (1.51%)
Transcripts of Fusion Genes	136 (0.42%)	51 (0.23%)	74 (0.4%)	297 (0.64%)
Fusion genes with more than one transcript	22 (20.37%)	5 (11.11%)	10 (17.24%)	53 (24.31%)
Transcripts with Intron Retention	5231 (15.95%)	2383 (10.81%)	2783 (14.95%)	6803 (14.59%)
Genes with Intron Retention	2566 (19.79%)	1422 (12.9%)	1589 (16.42%)	3375 (22.98%)
Protein-coding, IR-transcripts	4983 (95.26%)	2281 (95.72%)	2649 (95.19%)	6453 (94.86%)
IR-transcripts with canonical splice junctions	5231 (100%)	2383 (100%)	2760 (99.17%)	6766 (99.46%)

Table S6: Overview of the human and mouse cortical transcriptomes characterized using Iso-Seq, related to Table 1.
lncRNA – long non-coding RNA, FSM – Full Splice Match, ISM – Incomplete Splice Match, NIC – Novel In Catalogue, NNC – Novel Not in Catalogue, NMD – Nonsense-mediated mRNA decay

Gene	Iso-Seq Dataset	Peptide	PB.ID	Event
RGS11	Adult Cortex	GAELAGIGVGLR	PB.7368.23	Intron Retention
ARHGEF11	Adult Cortex	SSSQSIKPGNVR	PB.1416.6	Exon Skipping
VTI1A	Fetal Cortex	NELLGDDGNSSENQ LIK	PB.2147.4	Novel Exon Inclusion
RELCH	Fetal Cortex	VAEHEVPLQER	PB.7004.2	Exon Skipping
EPB41L1	Fetal Cortex	SLSPTVK	PB.9417.2	Exon Skipping

Table S8: Examples of proteomic support for novel transcripts identified using Iso-Seq, related to Figures 2 and 5. We identified five novel peptides, each mapping uniquely to a single novel transcript, providing evidence for the stable translation of novel isoforms in the human cortex. PB.ID refers to the specific Iso-Seq transcript supported. Peptide support for novel exon inclusion in *VTI1A* is illustrated in **Figure 2E**, intron retention in *RGS11* in **Figure 5F**, and novel exon skipping in *RELCH* in **Figure 5G**.

A)

Splicing event	Number and proportion of splicing events			
	**Human Cortex**	**Human (Adult) Cortex**	**Human (Fetal) Cortex**	**Mouse Cortex**
A3	1707 (6.49%)	1057 (7.15%)	737 (6.16%)	3217 (7.99%)
A5	915 (3.48%)	561 (3.79%)	380 (3.18%)	1883 (4.68%)
AF	8456 (32.16%)	5364 (36.26%)	3504 (29.31%)	12853 (31.93%)
AL	3883 (14.77%)	2163 (14.62%)	1800 (15.06%)	6182 (15.36%)
IR	5231 (19.89%)	2383 (16.11%)	2783 (23.28%)	6803 (16.9%)
MX	328 (1.25%)	142 (0.96%)	109 (0.91%)	625 (1.55%)
SE	5776 (21.97%)	3123 (21.11%)	2642 (22.1%)	8686 (21.58%)

B)

Splicing event	Number and proportion of genes with splicing events			
	**Human Cortex**	**Human (Adult) Cortex**	**Human (Fetal) Cortex**	**Mouse Cortex**
A3	1045 (8.06%)	677 (6.14%)	521 (5.38%)	1754 (11.94%)
A5	604 (4.66%)	408 (3.7%)	281 (2.9%)	1134 (7.72%)
AF	4879 (37.63%)	3357 (30.46%)	2451 (25.32%)	6476 (44.1%)
AL	2920 (22.52%)	1749 (15.87%)	1469 (15.18%)	4262 (29.02%)
IR	2566 (19.79%)	1422 (12.9%)	1589 (16.42%)	3375 (22.98%)
MX	256 (1.97%)	116 (1.05%)	88 (0.91%)	434 (2.96%)
SE	3446 (26.58%)	2093 (18.99%)	1830 (18.91%)	4570 (31.12%)

Table S12: Alternative splicing events observed in human and mouse cortex, related to Figure 5. Tabulated are the **A)** number of splicing events and B**)** number of genes observed with those splicing event, in human cortex (n = 7 biologically independent samples), human adult cortex (n = 4 biologically independent samples), human fetal cortex (n = 3 biologically independent samples), and mouse cortex (n = 12 biologically independent samples). Of note, a single gene can be characterised by multiple splicing events and can thus appear more than once in **B)**. The percentage refer to the proportion of total number of detected genes. A combination of the *SUPPA2* package and custom analysis scripts were used to identify transcripts associated with i) exon skipping (SE), ii) mutually exclusive exon use (MX), iii) alternative first (AF) and last (AL) exons, iv) alternative 3’ and 5’ splice sites, and v) intron retention (IR).

Description	Fetal Hippocampus	Fetal Striatum
Unique Genes	5568	5974
Annotated Genes	5566 (99.96%)	5969 (99.92%)
Novel Genes	2 (0.04%)	5 (0.08%)
Transcripts	7253	8150
Annotated Transcripts	6250 (86.17%)	6873 (84.33%)
Novel Transcripts	1003 (13.83%)	1277 (15.67%)
FSM	5774 (79.61%)	6226 (76.39%)
ISM	476 (6.56%)	647 (7.94%)
NIC	839 (11.57%)	1040 (12.76%)
NNC	155 (2.14%)	218 (2.67%)
Genic Genomic	0 (0%)	6 (0.07%)
Antisense	1 (0.01%)	2 (0.02%)
Fusion	7 (0.1%)	8 (0.1%)
Intergenic	1 (0.01%)	3 (0.04%)
Genic Intron	5568	5974

Table S17: Summary of the fetal hippocampus and fetal striatum Iso-Seq datasets, related to Table 1. FSM – Full Splice Match, ISM – Incomplete Splice Match, NIC – Novel In Catalogue, NNC – Novel Not in Catalogue

Description	Human Cortex			Mouse Cortex		
	AD	SZ	Autism	AD	SZ	Autism
Disease-associated genes	62	339	393	62	339	393
Detected disease-associated genes ("Detected")	33	288	317	41	318	342
Total Number of Transcripts	128	967	1042	201	1462	1782
Number and % of Annotated Transcripts	72 (56.25%)	558 (57.7%)	669 (64.2%)	93 (46.27%)	641 (43.84%)	763 (42.82%)
Number and % of Novel Transcripts	56 (43.75%)	409 (42.3%)	373 (35.8%)	108 (53.73%)	821 (56.16%)	1019 (57.18%)
FSM	50	424	412	71	492	485
ISM	22	134	257	22	149	278
NIC	43	313	288	61	523	670
NNC	13	96	85	47	298	349
Genic Genomic	0	0	0	0	0	0
Antisense	0	0	0	0	0	0
Fusion	0	0	0	0	0	0
Intergenic	0	0	0	0	0	0
Genic Intron	0	0	0	0	0	0
IR Genes (% of all IR Genes)	9(0.35%)	75(2.92%)	62(2.42%)	9(0.27%)	103(3.05%)	98(2.9%)
IR Genes (% of Detected)	9(27.27%)	75(26.04%)	62(19.56%)	9(21.95%)	103(32.39%)	98(28.65%)
NMD Genes (% of Detected)	8(24.24%)	58(20.14%)	47(14.83%)	9(21.95%)	78(24.53%)	75(21.93%)
IR and NMD genes (% of Detected)	4(12.12%)	29(10.07%)	24(7.57%)	4(9.76%)	44(13.84%)	36(10.53%)
Fusion Genes	1	5	8	1	10	10
Number of Detected Genes with >1 isoform	23(69.7%)	194(67.36%)	221(69.72%)	34(82.93%)	251(78.93%)	281(82.16%)

Table S19: Summary of transcripts mapping to disease-associated genes in human and mouse cortex, related to Figure 4. Isoform diversity was assessed in genes robustly associated with autism (393 genes nominated as being category 1 (high confidence) and category 2 (strong candidate) from the SFARI Gene database https://gene.sfari.org/), Alzheimer's disease (three familial AD genes and 59 genes nominated from the most recent GWAS meta-analysis) and schizophrenia (SZ) (339 genes nominated from the most recent GWAS meta-analysis). AD – Alzheimer's disease, SZ – Schizophrenia. IR – Intron retention, NMD – Nonsense-mediated mRNA decay, FSM – Full Splice Match, ISM – Incomplete Splice Match, NIC – Novel In Catalogue, NNC – Novel Not in Catalogue

**Iso-Seq Gene Expression threshold (Log_{10}TPM)**	**Human-Mouse correlation of number of isoforms**	**P-value**	**Number of genes surpassing expression threshold**
0	0.506	0	20404
0.5	0.502	0	19531
1	0.495	0	15023
1.5	0.509	0	9382
2	0.53	7.50×10^{-296}	4094
**2.5**	**0.543**	**4.91×10^{-87}**	**1123**
3	0.635	2.51×10^{-25}	212
3.5	0.751	1.33×10^{-07}	36
4	-0.091	NA	4

Table S22: Determining a common high gene expression threshold between human and mouse, related to STAR Methods. A high gene expression threshold was applied to a few analyses to further understand the relationship between isoform number and gene length (**Figure S5**), isoform number and gene exon number (**Figure S5C,S5D,S5G and S5H**), and to investigate whether there was a difference in intron retention rate between highly-expressed and lowly-expressed genes (**Figure S15G**). A gene expression cut-off was sequentially applied to both human and mouse cortex Iso-Seq dataset, and the number of isoforms of the filtered genes were then correlated. Subsequently, the gene expression threshold was determined by the gene expression at which number of isoforms for commonly expressed genes was most correlated between human and mouse with still a significant number surpassing the threshold – in this case, 2.5 Log_{10}TPM. Of note, the genes filtered could have an expression surpassing threshold in mouse but not in human, and vice versa. TPM – Transcripts per Million
